# Natural and directed antigenic drift of the H1 influenza virus hemagglutinin stalk domain

**DOI:** 10.1038/s41598-017-14931-7

**Published:** 2017-11-03

**Authors:** Christopher S. Anderson, Sandra Ortega, Francisco A. Chaves, Amelia M. Clark, Hongmei Yang, David J. Topham, Marta L. DeDiego

**Affiliations:** 1 David H. Smith Center for Vaccine Biology and Immunology, and Department of Microbiology and Immunology, Rochester, NY United States; 2 Department of Biostatistics and Computational Biology, University of Rochester Medical Center, Rochester, NY 14642 United States

**Keywords:** Antibodies, Viral infection

## Abstract

The induction of antibodies specific for the influenza HA protein stalk domain is being pursued as a universal strategy against influenza virus infections. However, little work has been done looking at natural or induced antigenic variability in this domain and the effects on viral fitness. We analyzed human H1 HA head and stalk domain sequences and found substantial variability in both, although variability was highest in the head region. Furthermore, using human immune sera from pandemic A/California/04/2009 immune subjects and mAbs specific for the stalk domain, viruses were selected *in vitro* containing mutations in both domains that partially contributed to immune evasion. Recombinant viruses encoding amino acid changes in the HA stalk domain replicated well *in vitro*, and viruses incorporating two of the stalk mutations retained pathogenicity *in vivo*. These findings demonstrate that the HA protein stalk domain can undergo limited drift under immune pressure and the viruses can retain fitness and virulence *in vivo*, findings which are important to consider in the context of vaccination targeting this domain.

## Introduction

Influenza virus infections cause seasonal epidemics and occasional pandemics, when novel viruses are introduced into humans^1,2^. One of these pandemics occurred in 2009, when an outbreak of a swine-origin novel H1N1 influenza A virus (pH1N1) began in Mexico, and was rapidly disseminated worldwide^3,4^. Despite efforts to vaccinate, the WHO estimates that influenza virus result in 1 billion infections, 3–5 million cases of severe disease and 300,000–500,000 deaths annually^5^. IAVs are classified in subtypes, according to the major two surface glycoproteins hemagglutinin (HA), and neuraminidase (NA). In humans, the most frequent seasonal subtypes of IAVs are the H3N2 and the H1N1, and two lineages of IBVs. Accordingly, the seasonal influenza vaccines include 3 or 4 viral strains (H1N1, H3N2, and one or two influenza B viruses)^6,7^.

The HA receptor is a trimer consisting of HA1 and HA2 subunits. HA is synthesized as an immature polypeptide chain (HA0), which is activated upon cleavage by host proteases to yield two subunits, HA1 and HA2. HA1 forms the globular head domain containing the receptor-binding site (RBS), and is the least conserved segment of influenza virus. HA2 together with the N and C terminal HA1 residues forms a stalk domain, which includes the transmembrane region, and is relatively conserved^8–10^. Functionally, the stalk domain mediates the fusion of viral and endosomal membranes once the virus is taken up into the cell.

Seasonal influenza vaccination mainly induces largely strain-specific antibodies directed to the HA head region, which inhibit HA attachment to sialic acid-bearing receptors on the cell surface^11,12^. However, antibodies directed against the stalk domain, inhibiting HA-mediated fusion of viral and endosomal membranes necessary to release the virus genome in the cytoplasm^13^ have been detected in humans^9,13–15^, likely induced after natural infection^16,17^. In contrast to these findings, infection and even vaccination with the pH1N1 2009 virus induced high levels of antibodies specific for the stalk domain^17–21^. The Ab response to the seasonal vaccine reflects activation of abundant memory B cells that recognize immunodominant epitopes in the HA head. In this case, the number of memory B cells specific for conserved epitopes in the HA stalk are outcompeted by a high number of cells specific for head epitopes. In the exposure to a novel HA such as in the case of initial pH1N1 infection or vaccination, the activation of memory B cells that recognize conserved head HA epitopes is diminished because the HA epitopes in the pH1N1 virus are substantially novel. Therefore, the number of memory B cells specific for conserved epitopes in the HA stalk are not outcompeted^8,19,20^. According to this hypothesis, it has been shown that HA stalk-reactive antibodies are boosted following sequential infection with seasonal and pH1N1 viruses in mice^22^. Following pH1N1 vaccination, the amounts of induced head reactive antibodies were generally higher in older subjects (likely exposed to antigenically similar pH1N1 viruses), whereas the amounts of HA stalk-specific antibodies were generally higher in the youngest cohort (likely not exposed to similar pH1N1 viruses)^23^. The HA protein incorporates mutations through a process called antigenic drift, decreasing the efficacy of the vaccine^24^, requiring periodic updates to the seasonal vaccine to try to maintain a good match with circulating viruses^25–27^.

Because the antibodies against the dominant HA head domain are usually strain specific, and the HA head domain presents a high plasticity leading to antigenic drift, antibodies against the more conserved HA stalk domain are currently being discussed as promising therapeutic targets^8^. The “universal” vaccines based on stalk-specific antibodies may present several advantages over the current vaccines such as conferring protection against drifted influenza virus strains, abolishing the need for annual reformulation of the vaccine strains, and conferring increased protection against newly emerging influenza viruses with pandemic potential^8,28^. However, few studies have addressed variability in the HA stalk domain^29^ and whereas some studies have described the selection of escape mutants using stalk-specific mAbs^30–33^, no studies have analyzed whether under immune pressure using human sera, the stalk domain could eventually drift, decreasing the efficacy of HA stalk domain-based vaccines.

The potential advantages of stalk-based universal influenza vaccination strategies are based on a number of assumptions. One is that the stalk domain is relatively conserved compared to the head domain and contains epitopes common to many strains of influenza. This notion is supported by studies showing broad cross-reactivity of stalk-specific monoclonal antibodies. In contrast, Su *et al*. used a statistical approach to demonstrate that amino acid sites in the HA stalk were positively selected in the 2009 pandemic-like strains, demonstrating that amino acid changes in the stalk region occur both during and after host adaption, consistent with antigenic drift of the stalk region^34^. Another assumption is that stalk-specific antibodies are rare in the human population and, therefore, inefficiently drive selection of antigenic variation. A third not well supported assumption widely circulated in the field is that selection of HA stalk mutants by immune sera leads to escape mutants that are relatively unfit compared to their unmutated progenitors. Here we provide evidence that all three of those assumptions are not necessarily true in all circumstances. We show that HA protein stalk domain presents variability, suggesting it could also be subjected to antigenic drift. Sequence analysis showed positive selection and a non-random path of evolution over time in the HA stalk domain. Culturing pH1N1 virus in the presence of human immune sera and stalk-specific mAbs selected mutant viruses whose growth was less inhibited in the presence of antibodies and that were fit in terms of replication and pathogenesis. Collectively, the data suggest that natural and immune directed selection of antigenic variants in the HA stalk domain can eventually occur, though likely at a slower rate than changes to the head domain.

## Results

### HA stalk and head domains present sequence variability

**Figure 1:**
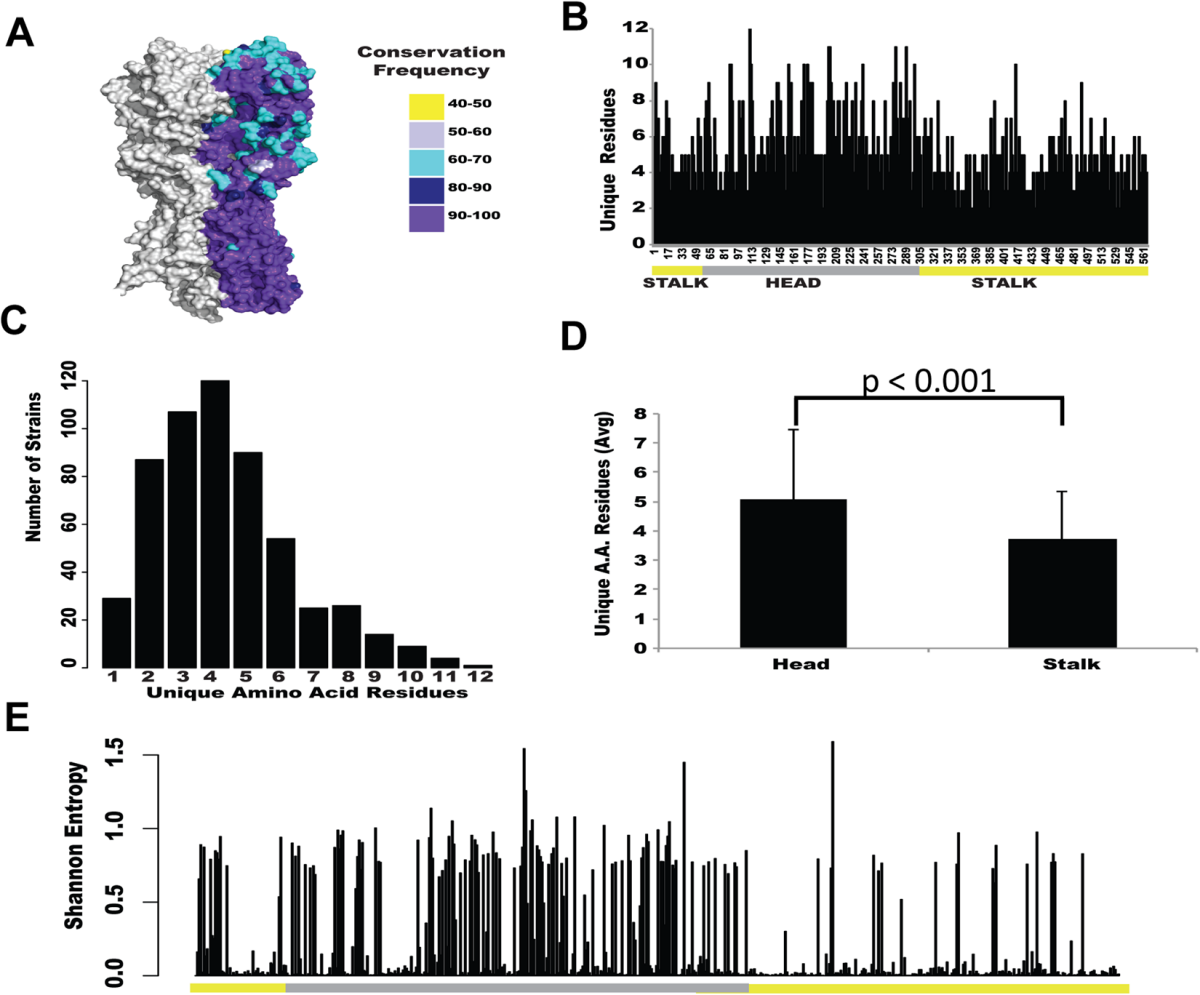
Amino acid usage across influenza H1N1 HA proteins. Analysis of HA protein sequence variability among the 4827 H1N1 strains publically available in Influenza Resources Database. (**A**) 3D structure of A/California/04/2009 hemagglutinin protein. Each aa is colored by its conservation frequency, meaning the percent of viruses that have the dominant (most common) amino acid at that position. (**B**) Number of unique aa found at each position of the HA protein (566 residues). HA head region is indicated in gray, stalk in yellow. (**C**) Histogram of the number of strains for the range of unique aa found in (**B**). (**D**) Average number of aa used at each position for head or stalk region. P-value <0.001 using a Welch’s t-test. (**E**) Shannon entropy (unpredictability) at each aa position across HA protein.

In order to address the ability of the stalk region to incorporate aa substitutions, publically available influenza H1N1 HA protein sequences were obtained from viruses that circulated since 1918. The percentage of sequences that contain the most common aa at a given position, was higher in the stalk region compared to the head (Fig. 1A). Multiple amino acid substitutions were found at aa positions in both the head and stalk regions (aa 1–566, being aa 1 the initial Met, Fig. 1B). The number of different aa at one particular position ranged from one to twelve with the majority of positions having four unique aa (Fig. 1C). The greatest number of aa usage (12) was at a single residue at HA position 111 in the head region. Head positions averaged 5.065 unique aa, whereas stalk positions averaged 3.71 (Fig. 1D). Shannon entropy is a measure of aa conservation^35,36^. Values can range from 0 (constant amino acid) to 4.32 (all amino acids represented equally), with values greater than 2 considered being highly variable^35,36^. All HA protein aa had a Shannon Entropy (SE) value of less than 2.0, showing that the influenza HA is generally conserved (Fig. 1E). The HA head region contained 11 aa with SE values above 1.0 and the stalk region contained one. Generally, SE varied across HA with the head region having greater SE values compared to the stalk, although values above 0.5 were found in both regions (Fig. 1E). Similar results were found when the rate of non-synonymous substitutions (dN) was calculated (Supplementary Figure 1). Taken together, these results suggest that the ability to incorporate aa changes is lower for the stalk regions than for the head region, probably due to differences in immune pressure, and/or that the stalk domain is less tolerant to mutation than the head domain^37,38^.

**Figure 2:**
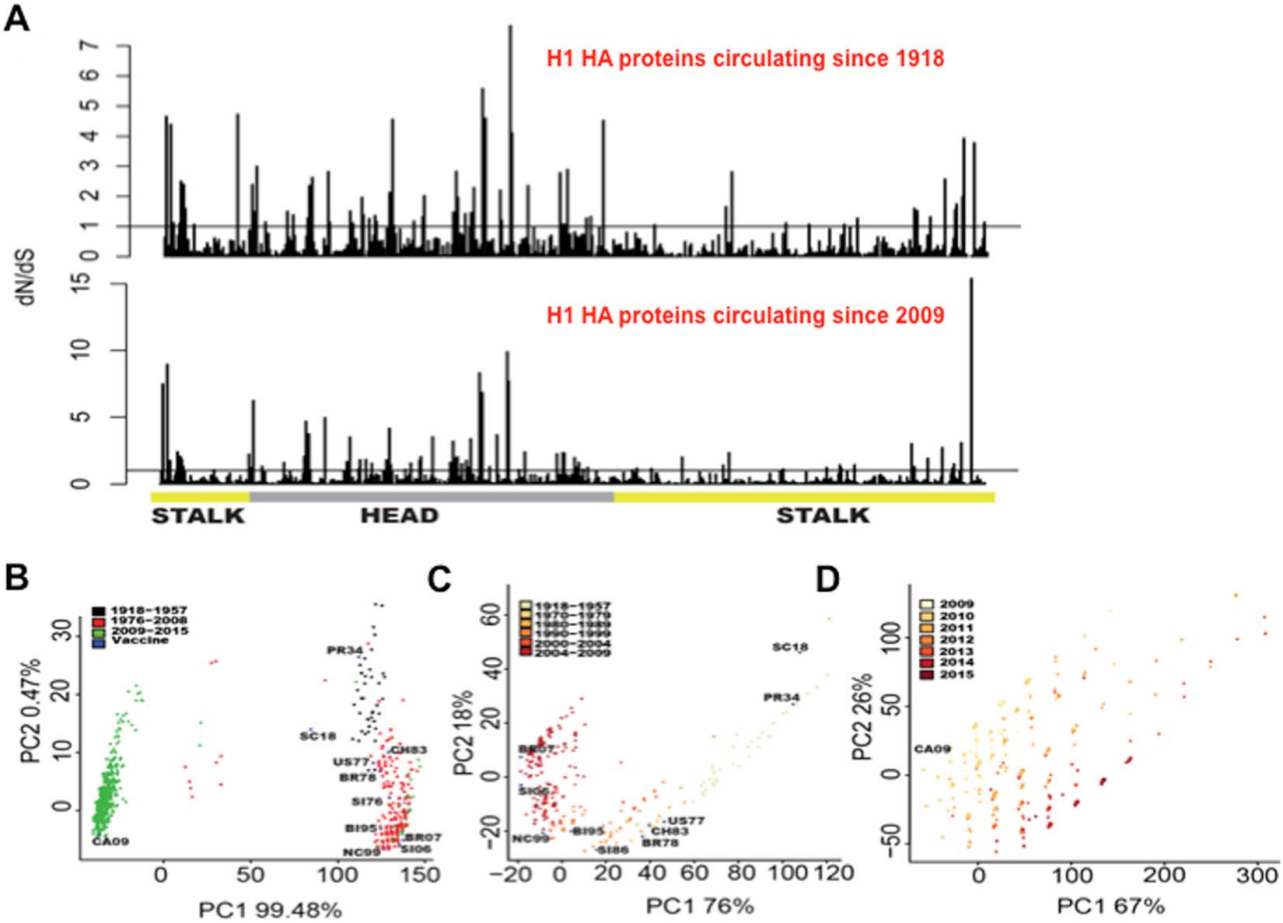
Variability of influenza H1N1 HA protein. (**A**) dN/dS analysis showing positive selection of codons across the H1 HA proteins of viruses that circulated since 1918 (top) and those that circulated since the 2009 pandemic (bottom). Values greater than one (bar) are considered positively selected (see Supplementary Tables 1 and 2 for exact values). (**B**) Principal component analysis (PCA) of protein amino acid changes between the stalk regions of influenza strains. HA proteins from viruses that circulated from 1918–2015, colored as indicated in legend. (**C**) PCA of only 1918-pandemic lineage viruses; viruses that circulated from 1918–2009. (**D**) PCA of 2009-pandemic lineage viruses that circulated from 2009–2015.

To further explore these genetic changes in HA, selection analysis (dN/dS or Ka/Ks ratio)^39^ was performed on 11,535 H1 DNA sequences from viruses circulating since 1918 (Fig. 2A, top panel). Most codons were negatively selected over time (387 sites) with positive selection occurring at 30% of the sites (175) and 4 neutral selection sites. Positive selected sites occurred with a frequency of 48.44% in head (109 residues out of 225 residues), and with a frequency of 19.35% in stalk (66 residues out of 341 residues) regions (Fig. 2A, top panel, and Supplementary Table 1), showing that overall, positive selection occurred across the HA protein with a decreased frequency in the stalk region compared to the head. Next, dN/dS ratio was calculated on post 2009 pandemic viruses HA proteins (6604 H1 DNA sequences, Fig. 2A, lower panel, and Supplementary Table 2). Overall results were similar to the analysis using all H1 sequences, showing positive selection in both head and stalk domains (Fig. 2A, lower panel).

HA protein sequence data has been used to estimate antigenic differences between strains^40,41^. Unlike the HA head, epitope locations in the stalk are not well defined. Therefore, we used the sequence for the complete stalk region (amino acids 1–59 & 292–567) in a sequence variation analysis, similar to what has been described for H5 sequences^40^. Sequence variation was determined using an information theory based approach where the number of positions that differ between aligned protein sequences are counted. Principal component analysis (PCA) was performed in order to reduce the dimensionality of the sequence variation data to identify trends and clustering among the set of observations (virus strains) using a set of input variables (HA sequence difference counts). By using the number of amino acid differences between strains as input variables, PCA can be used to identify trends in sequence variation. The results can be plotted in two dimensions with each dimension accounting for a proportion of the variation in sequence^40,42^. Additionally, since PCA finds the greatest variation in the dataset, even if only a single sequence is very different from all others, this difference will be observed in the PC1 axis^42^. Therefore, this analysis is not highly sensitive to differences in the number of sequences obtained each year. Given the limited HA sequence data available from early 20^th^ century viruses, PCA is an appropriate method. Two large clusters were found, one containing the 1918 pandemic and swine lineage strains (1918–1957 and 1976–2008) and the other containing the 2009 pandemic strains (Fig. 2B, points colored by year of isolation). Interestingly, the stalk region of the 1918 pandemic strain (SC18), whose head epitopes are similar to the 2009 pandemic strain^43^, did not cluster with 2009-pandemic related strains. To better assess protein drift among these sequence clusters, 1918-lineage or 2009-lineage strains were analyzed separately (Fig. 2C,D, respectively). 1918-lineage strains form a linear trend starting with the 1918 pandemic strain (SC18) and ending with the 2007 vaccine-like strain (BR07; Fig. 2C) and PC1 was significantly associated with year of isolation (ANOVA p<0.001). Similarly, 2009-pandemic strain lineage viruses showed a linear trend in PC1 that was also significantly associated with year of isolation (ANOVA p<0.001; Fig. 2D). Together with the positive selection analysis, these results are consistent with the accumulation of mutations over time probably due to antibody pressure from an increasingly immune population and support that protein drift can occur in the stalk region and that these substitutions are not random but directional over time.

### Changes in antigenicity in H1N1 HA stalk domain

**Figure 3:**
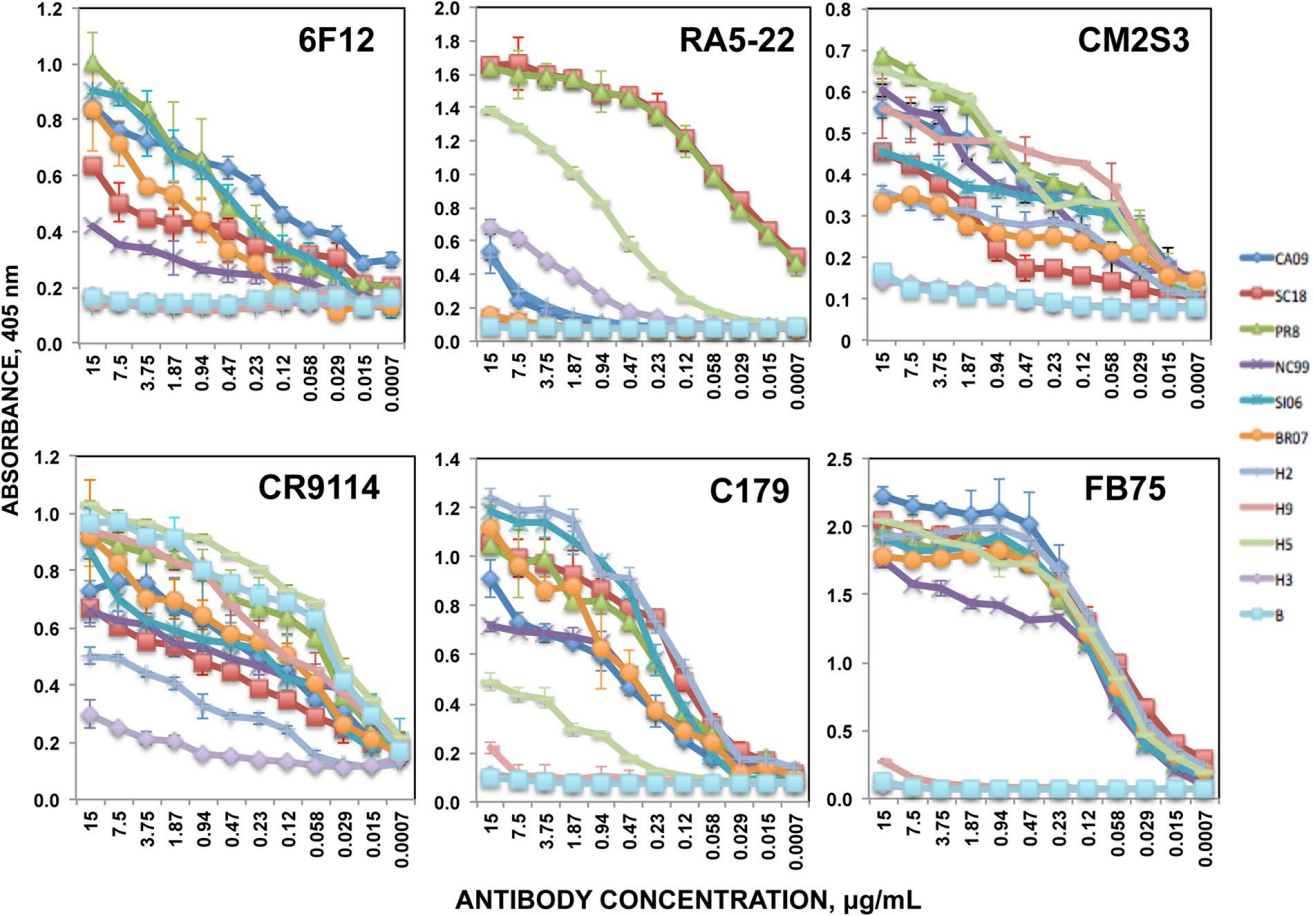
Antigenic variation in the stalk region of historical influenza viruses. ELISA titers were measured using seven monoclonal antibodies reactive to the stalk region of HA protein (6F12, RA5-22, CM2S3, CR9114, C179, and FB75) against recombinant HA from 6 H1N1 viruses and 5 other subtypes (H2, H9, H5, H3, and influenza **B**). The assays were performed in duplicates, twice, and the averages are shown. Purified HA proteins from the following strains were used: H1N1 strains A/California/04/2009 (CA09), A/South Carolina/11/1918 (SC18), A/Puerto Rico/8/1934 (PR8), A/New Caledonia/20/1999 (NC99), A/Solomon Islands/3/2006 (SI06), A/Brisbane/59/2007 (BR07), and H2N2 A/Singapore/1/1957 (H2), H9N2 A/Hong Kong/33982/2009 (H9), A/Indonesia/05/2005 (H5), H3N2 A/Brisbane/10/2007 (H3), and B/Brisbane/60/2008 (B).

To analyze whether the HA stalk domains from different strains are antigenically distinct, ELISA were performed using 8 mAbs specific to the HA stalk region (6F12, RA5-22, CM2S3, CR9114, C179, FB75, F49, and B198M) at 12 different 2-fold dilutions and 12 historical influenza strains including 6 H1N1 strains, H2, H9, H5, H3 and influenza B strains (Fig. 3 and Supplementary Figure 2). mAb 6F12 bound all the HA proteins from H1N1, but it did not bind the HAs from H2, H3, H5 and B strains, similarly to what was shown in previous reports^31^. Interestingly, consistent differences in binding with this mAb 6F12 could be observed among H1N1 strains, being the HA proteins from A/South Carolina/11/1918 and A/New Caledonia/20/1999 strains, the ones showing the lowest binding (Fig. 3). mAb RA5-22 bound HAs from strains A/South Carolina/11/1918, A/Puerto Rico/8/1934, and to a lower extent A/California/04/2009, H2, and H3, but it did not bind the other H1N1 strains and the H5, H9, and B strains. However, contrary to the other mAbs used in this study, the mAb RA5-22 mainly recognizes a linear epitope exposed in the denatured form of the protein^44^, therefore, although the ELISA data with this mAb probably account for antigenic variability within H1N1 strains, the biological significance of these results could be limited. mAb CM2S3 bound all the H1N1, H2, H5 and H9, but not to H3, and B, similar to previous results showing that this antibody does not bind H3 and B strains^45^. Consistently, differences in binding with the mAb CM2S3 could be observed among H1N1 strains, with the HA proteins from A/Solomon Islands/3/2006 (H1N1) and A/Brisbane/59/2007 strains showing the lowest binding (Fig. 3). CR9114 bound all the HA proteins from the different strains to different extents (Fig. 3). Particularly, HAs from H3 and H2 strains showed decreased binding (Fig. 3). Similarly, using transfected cells expressing different HA proteins in binding experiments, and whole viruses in microneutralization experiments, dissociation constants (Kd) and *in vitro* neutralizing activity (IC_50_), respectively, were not the same for all the H1N1 strains tested^46^, further confirming that although this antibody binds all HA strains tested, differences in binding for the different strains are detected. mAb C179 bound H1N1 strains, H2 and H5 strains, however, it did not bind HAs from H3, H9 and B (Fig. 3), as previously described for H3 and B^47^. Using this mAb, there were slight differences in binding, being the HAs from the strains A/New Caledonia/20/1999 and A/California/04/2009 the ones showing decreased binding (Fig. 3). Similarly, staining mAb titers using infected cells were not the same for all H1N1 and H2N2 strains tested^48^. mAb FB75 bound HA proteins from all influenza A strains, except H9 and H3 (Fig. 3), as previously described^47^. However, among the HA proteins from H1N1 strains, the HA protein from A/New Caledonia/20/1999 showed decreased binding (Fig. 3). Similarly, in previous reports the EC_50_ measured by ELISA were not the same for all the H1N1 strains tested^47^. mAb F49, and B198M only bound H3, and influenza B (Supplementary Figure 2), respectively, according to previous results showing that F49 mAb does bind influenza A H3N2 strains, but it does not bind H1N1, H2N2 and influenza B strains^48^. These results demonstrate that even with a limited number of stalk specific mAbs, differences in binding among subtypes, and more importantly, among H1N1 strains, can be detected, indicating that even within the H1N1 strains, the stalk domain is not antigenically identical.

### Selection of HA variants by growing influenza virus under immune pressure

To better determine if antigenic changes can occur in the stalk region, A/California/04/2009/E3^49^ was passaged in the presence of five human sera from subjects either infected or vaccinated with pH1N1 viruses (Supplementary Table 3), and in the presence of two different human and mouse mAbs recognizing the stalk domain^31,46^. HAI using the virus A/California/04/2009/E3, showed antibody titers ≥40 for sera from subjects FAM195, FAM196, FAM297, FAM298 and FAM300 (Supplementary Table 3). Moreover, specific signals in ELISA assays were obtained for the full length H1 protein (Supplementary Figure 3A), and, interestingly, also for the cH5/H1 and cH6/H1 proteins (Supplementary Figure 3B and C). These data suggested that the sera from the different subjects contained antibodies specific for the HA head and stalk domains.

**Figure 4:**
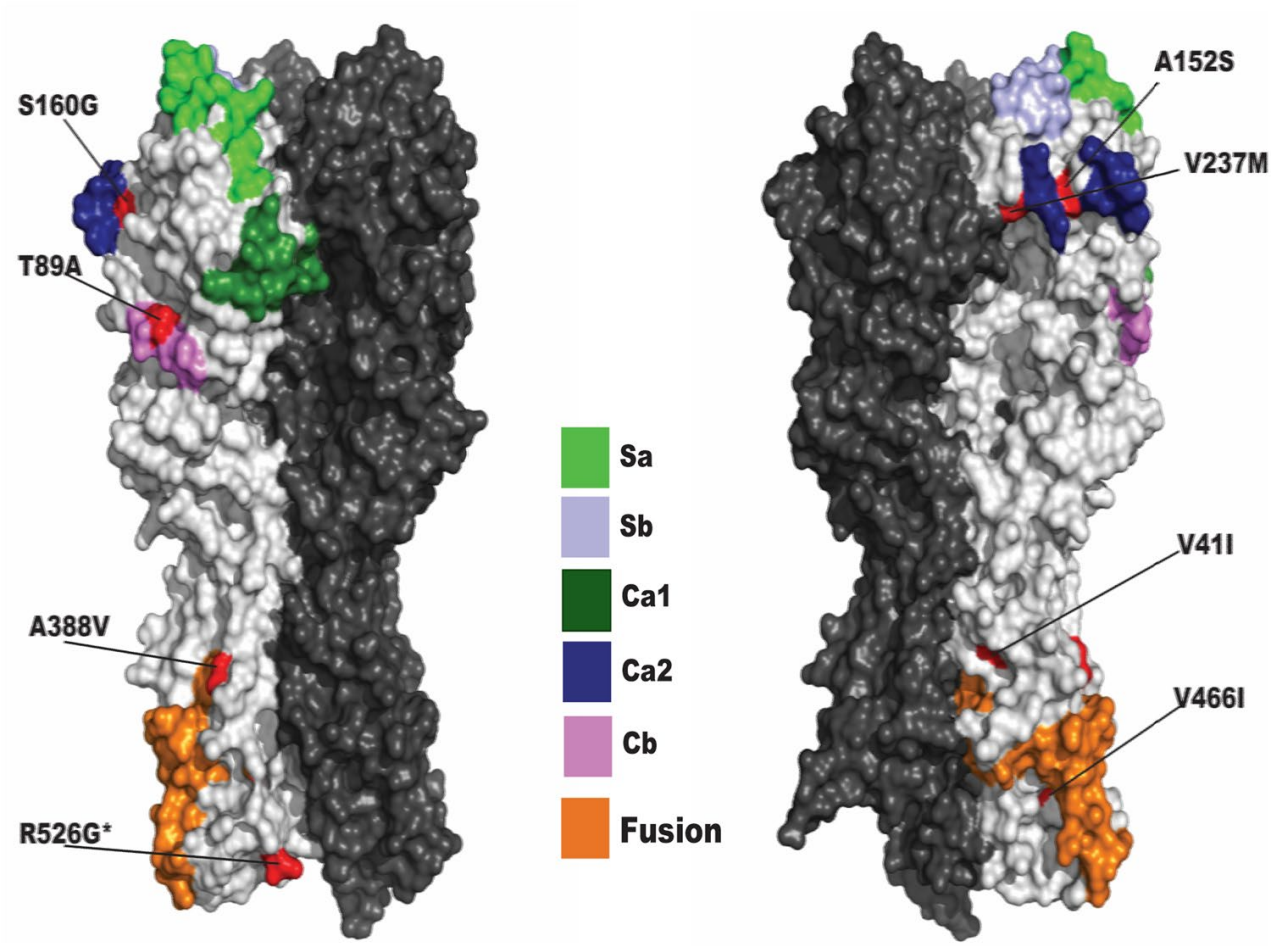
Amino acid locations of escape mutations. 3D structure simulation of the HA protein reconstructed using the 2009-pandemic virus protein sequence. Canonical B cell epitopes are colored as indicated in legend, mutations are colored in red. The fusion peptide in the HA2 domain is colored in orange. The antigenic sites are colored differently (see legend). *Indicates this is not the specific position for the aa 526, since it is in the conformationally unstable linker domain (the crystal structures of HA all end at residue 510)^60^.

After passaging A/California/04/2009/E3 virus 16 times in MDCK cells in the presence of the different human sera and mAbs, mutations in the HA protein head and stalk domains were found, although at different passages (Table 1). Specifically, serum from subject FAM195 selected a mutation in the head domain (V237M), in antigenic site Ca2; sera from subjects FAM196 and FAM297 selected a mutation in the head domain (A152S); sera from subject FAM298 selected a mutation in the stalk domain (V41I), and the mutation A152S; sera from subject FAM300 selected two mutations in the head domain (T89A, and S160G), in the described antigenic sites Cb, and Ca2, respectively^50^; and the two mAbs selected 3 mutations in the stalk domain (the CR9114 mAb selected mutations V466I, and R526G, and the 6F12 mAb selected the mutation A388V) (Table 1 and Fig. 4). As controls, the virus was passaged 16 times in the presence of two human sera (from patients FAM203 and FAM256) showing low HAI titers (<10, Supplementary Table 3), in the presence of human and mouse IgG isotype controls, and in the presence of no sera/antibodies (Table 1). None of the mutations selected under immune pressure were selected in these cases. We found no mutations in the stalk domain, and only two mutations in the head domain (Table 1). However, these two mutations in the head domain were not in previously described antigenic sites^50^. These data suggested that under immune pressure, mutations in the HA stalk and head domains can occur and are not randomly selected.

**Table 1:** Mutations found in the HA protein after growing the virus A/California/04/2009/E3 in the presence of human sera and mAbs.

SERA	MUTATIONS (ANTIGENIC SITE)	FIRST PASSAGE SHOWING THE MUTATION
FAM195	V237M (Ca2)	6
FAM196	A152S	4
FAM297	A152S	4
FAM298	V41I (stalk)	11
	A152S	4
FAM300	T89A (Cb)	13
	S160G (Ca2)	4
CR9114	V466I (stalk)	16
	R526G (stalk)	16
6F12	A388V (stalk)	8
**controls**		
none	—	—
FAM203	N146S	7
FAM256	A214T	6
human IgG	—	—
mouse IgG	—	—

### Relevance of the HA head domain mutations in evading the antibody immune response

To test whether the variants selected in the head domain of the HA protein contribute to evasion of the antibody immune response, HAI assays were performed with the sera used for selecting each mutation, comparing the reactivity against the starting virus (A/California/04/2009/E3), and the selected variants (Table 2). Interestingly, HAI titers for the viruses incorporating the mutations V237M, A152S, and V41I/A152S, were 2-3 fold lower and HAI titers for the virus incorporating the mutations T89A/S160G, were 4-fold lower that those observed for the original virus (Table 2). To confirm these results using another approach, MN assays were performed. Interestingly, MN titers for the viruses incorporating the mutations V237M, A152S, and T89A/S160G, were around 2-fold lower than titers specific for the original virus. In addition, MN titers for the virus incorporating the mutations V41I/A152S were 5-fold lower that those observed for the original virus (Table 2). Importantly, MN antibody titers using an HA stalk specific antibody (CR9114) were the same for all the viruses (data not shown), strongly suggesting that the mutations selected specifically affect the binding to anti-head antibodies, not affecting global sensitivity to any antibody. The NA gene from all the viruses encoded the same aa sequence (data not shown), indicating that the differences observed in the MN assays are due to antibodies specific for the HA, and not for the NA. Altogether, data from both assays indicated that under immune pressure, mutations in the HA protein head domain that contribute to evasion of the antibody response, are selected.

**Table 2:** Subject sera HAI titers specific for A/California/04/2009/E3 (original), and the viruses passaged 16 times in MDCK cells under immune pressure.

SERA	virus/mutation(s)	HAI TITER*	MN TITER*
		mean ± stdev	mean ± stdev
FAM195	195-p16 (V237M)	1066±330	11093±5033
	original	2773±1258	22186±10067
FAM196	196-p16 (A152S)	173±78	2133±660
	original	426±165	5546±2516
FAM195	298-p16 (V41I/ A152S)	533±165	5546±2516
	original	1493±522	28773±4180
FAM300	300-p16 (T89A/S160G)	86±39	1493±522
	original	346±157	2346±522

*Experiments were repeated three times in duplicate. Means and standard deviations are shown. P-values using a Student’s t-test were <0.05 for the comparison between the original virus and the viruses incorporating the selected mutations in all the cases.

### Relevance of the HA stalk domain mutations in evading the antibody immune response

**Figure 5:**
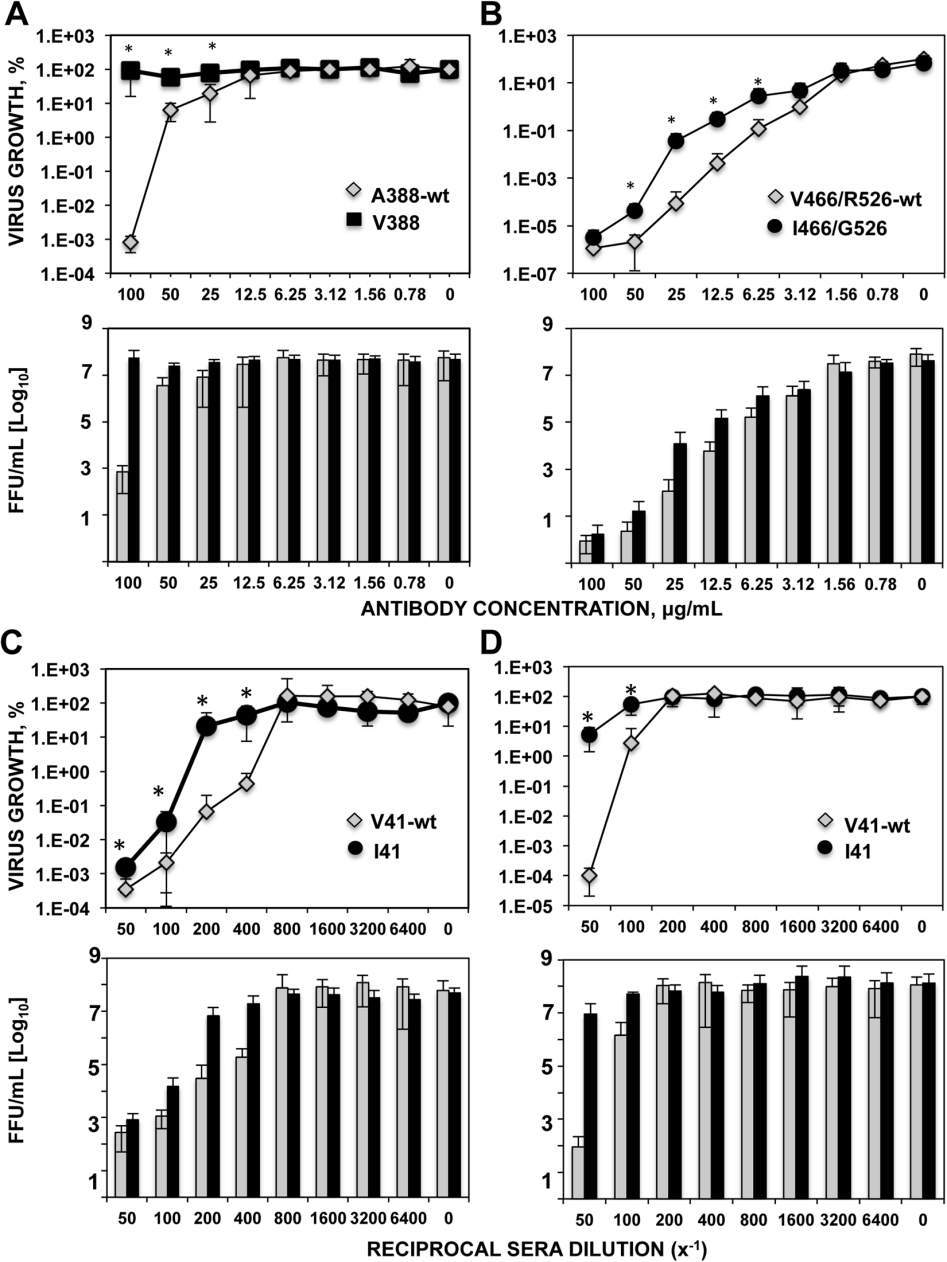
Microneutralization assays for the viruses incorporating mutations in the HA protein stalk domain. The original virus or the viruses incorporating the mutations A388V (**A**), V466I/R526G (**B**), or V41I (**C** and **D**) were grown in the presence of the mAbs 6F12 (**A**), and CR9114 (**B**), or in the presence of serum from subject 298 directly (**C**) or pre-absorbed with the head domain of the HA protein from A/California/04/2009 (**D**). Virus growth in the presence of different concentrations of mAb/serum was compared to virus growth in the absence of mAbs/serum, and represented as % growth (upper panels) or as virus titers (in FFU/ml, lower panels). Experiments were repeated three independent times in duplicate, with similar results. Data represents the means and standard deviations (SD) from the three experiments. *p-values <0.05 for comparison of wt versus V41I, A388V or V466I/R526G mutants, using a Student’s t-test.

To determine the relevance of the mutations in the stalk domain selected in evading the antibody response, MN assays were performed. To this end, the original viruses, and the viruses incorporating the selected mutations (A388 vs V388 in Fig. 5A, V466/R526 vs I466/G526 in Fig. 5B, and V41 vs I41 in Figs. 5C and D), were incubated at different mAb/sera concentrations, and virus growth was analyzed at 48 hpi. Sera collected from patient 298 was used directly in Fig. 5C, whereas in Fig. 5D sera from patient 298 which was pre-absorbed with the HA head domain of HA A/California/07/2009 protein. The growth of all the viruses was inhibited in the presence of the mAbs/sera, indicating that they present virus-neutralizing activity. Interestingly, using all the mAbs/sera, the virus growth of the viruses incorporating the selected mutations (V388, I466/G526, and I41) was inhibited to a reduced extent in the presence of mAbs/sera, as compared to the growth of the original viruses (A388, V466/R526, and V41) (Fig. 5). As expected, using the subject 298 serum directly, the MN titers were higher than the titers using the HA head domain pre-absorbed sera, consistent with the idea that neutralizing antibodies specific for the HA head region were pre-absorbed. These data suggested that the stalk mutations selected in the presence of mAb/sera partially contribute to antibody evasion, as they affect the ability of the antibodies to neutralize virus growth.

**Figure 6:**
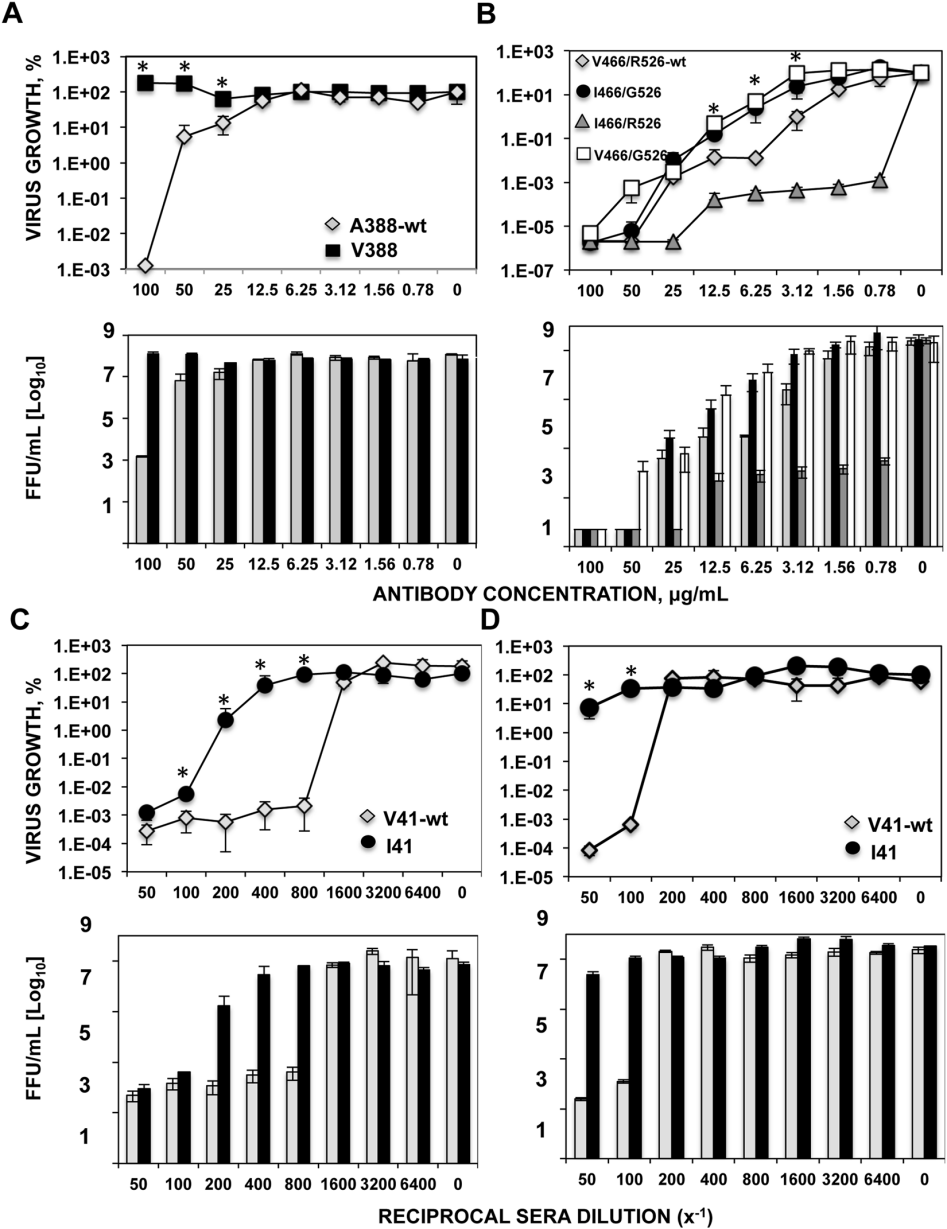
Microneutralization assays for the recombinant viruses incorporating the mutations in the stalk domain. The wt (original) virus or the recombinant viruses incorporating the mutations A388V (**A**), V466I/R526G alone or in combination (**B**), or V41I (**C** and **D**) were grown in the presence of the mAbs 6F12 (**A**), and CR9114 (**B**), or in the presence of serum from subject 298 directly (**C**) or pre-absorbed with the head domain of the HA protein from A/California/04/2009 (**D**). Virus growth in the presence of the mAb/serum was compared to virus growth in the absence of mAb/serum, and represented as % growth (top panels) or as virus titers (in FFU/ml, bottom panels). Experiments were repeated three independent times in duplicate, with similar results. Data represents the means and SDs from the three experiments from the three experiments. *p-values <0.05 for comparison of wt versus V41I, A388V, I466/G526 and V466/G526 viruses, using a Student’s t-test.

Recombinant viruses incorporating only the mutations in the HA protein were generated, and used for the MN assays described in the previous paragraph. Results using the wt virus, and the viruses incorporating mutations V388, and I41 were similar to those reported in Fig. 5A,C,D (Fig. 6A,C,D), further suggesting that the mutations V388 and I41 contribute to decrease the neutralization ability of mAb 6F12 and of sera 298, respectively. In the presence of mAb CR9114, the virus growth of the viruses I466/G526, and G526 was inhibited to a reduced extent compared to the growth of the wt virus or the virus incorporating the mutation I466 alone (Fig. 6B), further suggesting that the mutation G526, and not the mutation I466, is responsible for the partial escape from the antibodies observed. Importantly, the growth inhibition mediated by an anti-head antibody was the same for all the viruses with the exception of virus V466I, which showed a decreased sensitivity to antibodies, most probably because this virus grows with lower titers (Supplementary Figure 4), strongly suggesting that the mutations selected under immune pressure partially (R526G, and V41I) and more strongly (A388V) affect the antigenicity of the HA stalk domain.

**Figure 7:**
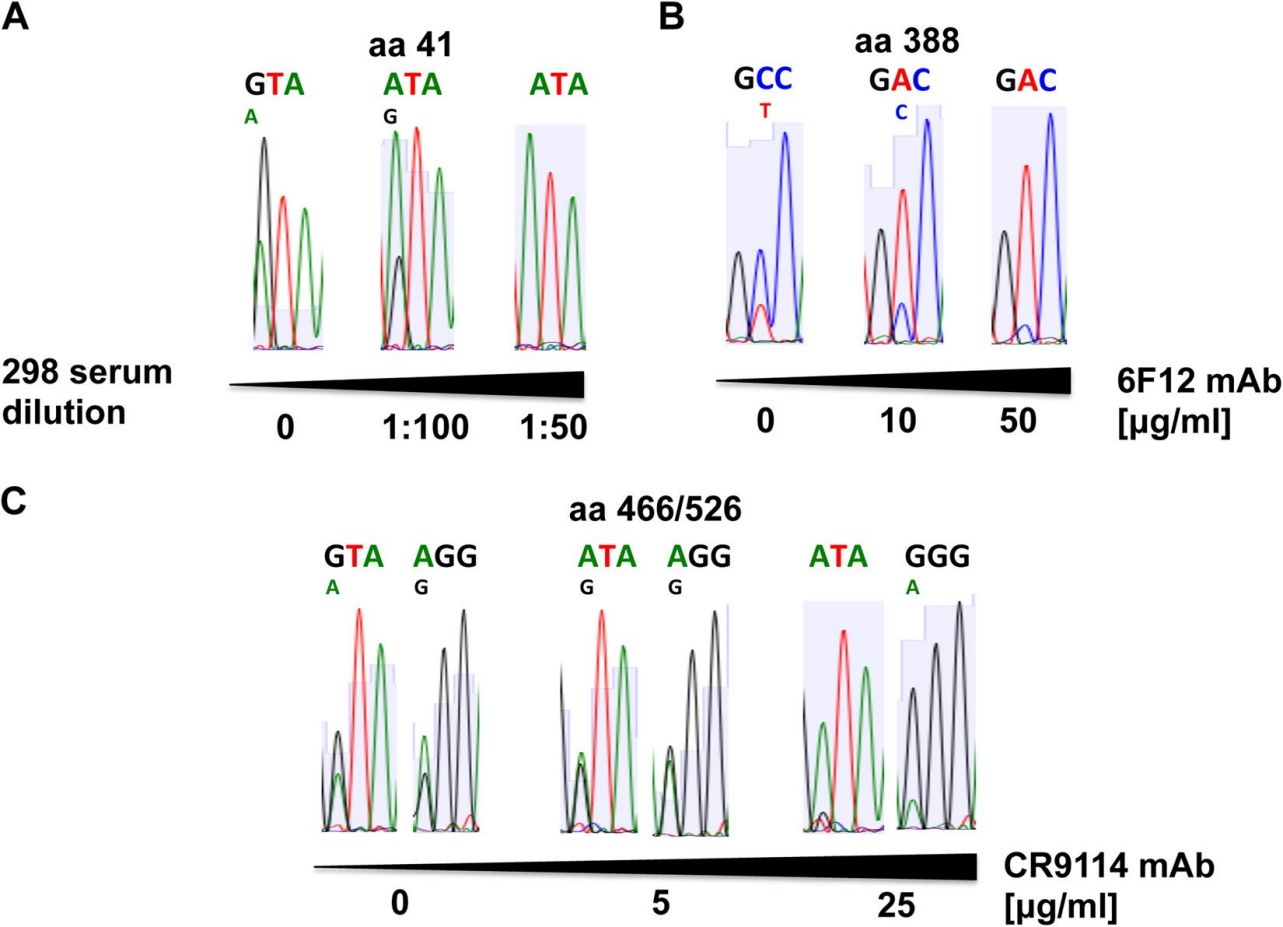
Co-infection of wt and viruses encoding the mutations selected under immune pressure. The wt virus and viruses incorporating the mutations V41I (**A**), A388V (**B**) and V466I/R526G (**C**) were used to co-infect cells at a ratio 2: 1 in the absence (0) or presence of two different concentrations of 298 serum (**A**), mAb 6F12 (**B**) or mAb CR9114 (**C**). When cytopathic effect was approximately 10%, viral and cellular RNA from infected cells was extracted and the haemagglutinin (HA) sequence was obtained. Chromatograms showing the sequences for amino acids 41 (**A**), 388 (**B**) and 466/526 (**C**) are shown. Letter represent the nucleotide sequence for codons 41 (GTA, encoding V, to ATA, encoding I, in **A**), 388 (GCC, encoding A, to GAC, encoding V, in **B**), and 466/526 (GTA/AGG, encoding V/R, to ATA/GGG, encoding I/G, in **C**).

To confirm the results showing that mutations V41I, A388V, and V466I/R526G affect the antigenicity of the protein, MDCK cells were co-infected with the original virus and the virus incorporating the mutations selected under immune pressure (Fig. 7), in the presence or absence of serum from patient 298, pre-absorbed for HA1 antibodies (wt+ I41 viruses), in the presence or absence of mAb CR9114 (wt + I466/G526 viruses), and mAb 6F12 (wt + V388 viruses). When CPE reached 10%, total RNAs were extracted and the HA gene was sequenced. In the absence of immune pressure, the predominant virus produced was the wt virus, since the wt selected viruses were mixed at a 2:1 proportion (Fig. 7). Interestingly, in the presence of the lowest concentration of 298 serum (Fig. 7A), mAb 6F12 (Fig. 7B) and mAb CR9114 (Fig. 7C), the predominant viruses were the viruses encoding the selected mutations V41I, A388V, and V466I/R526G, respectively. Moreover, in the presence of higher concentrations of serum/mAbs the vast majority of the viruses detected were the mutant viruses encoding the amino acid changes V41I, A388V, and V466I/R526G (Fig. 7). These data indicate that in the presence of immune pressure, but importantly, not in its absence, the viruses encoding the mutations selected present a selective advantage in growth. Furthermore, in plaque lysis assays, for the wt virus, the number of plaques was reduced by more than 90% using 1:450 dilution of 298 serum pre-absorbed for HA1 antibodies, however, only a 1:150 dilution was capable of inhibiting the number of plaques by more than 90% for the V41I virus. Whereas the number of plaques formed by the wt virus was reduced by more than 90% in the presence of 20 μg/ml of 6F12 mAb or of 0.18 μg/ml of CR9114 mAb, we observed no reduction in the number of plaques for the A388V virus, even in the presence of 60 μg/ml of 6F12 mAb and a higher concentration of 0.55 μg/ml of CR9114 mAb was necessary to reduce the number of plaques by more than 90% using the virus incorporating the mutations V466I/R526G (data not shown). These results suggest that the mutations selected under immune pressure V41I and R526G partially contribute to decrease the antibody-neutralization ability, whereas the mutation A388V has a stronger effect on the antigenicity of the protein. Although the V41I and R526G mutations only partially affect antibody reactivity, the competition experiments demonstrate these changes are biologically significant.

**Figure 8:**
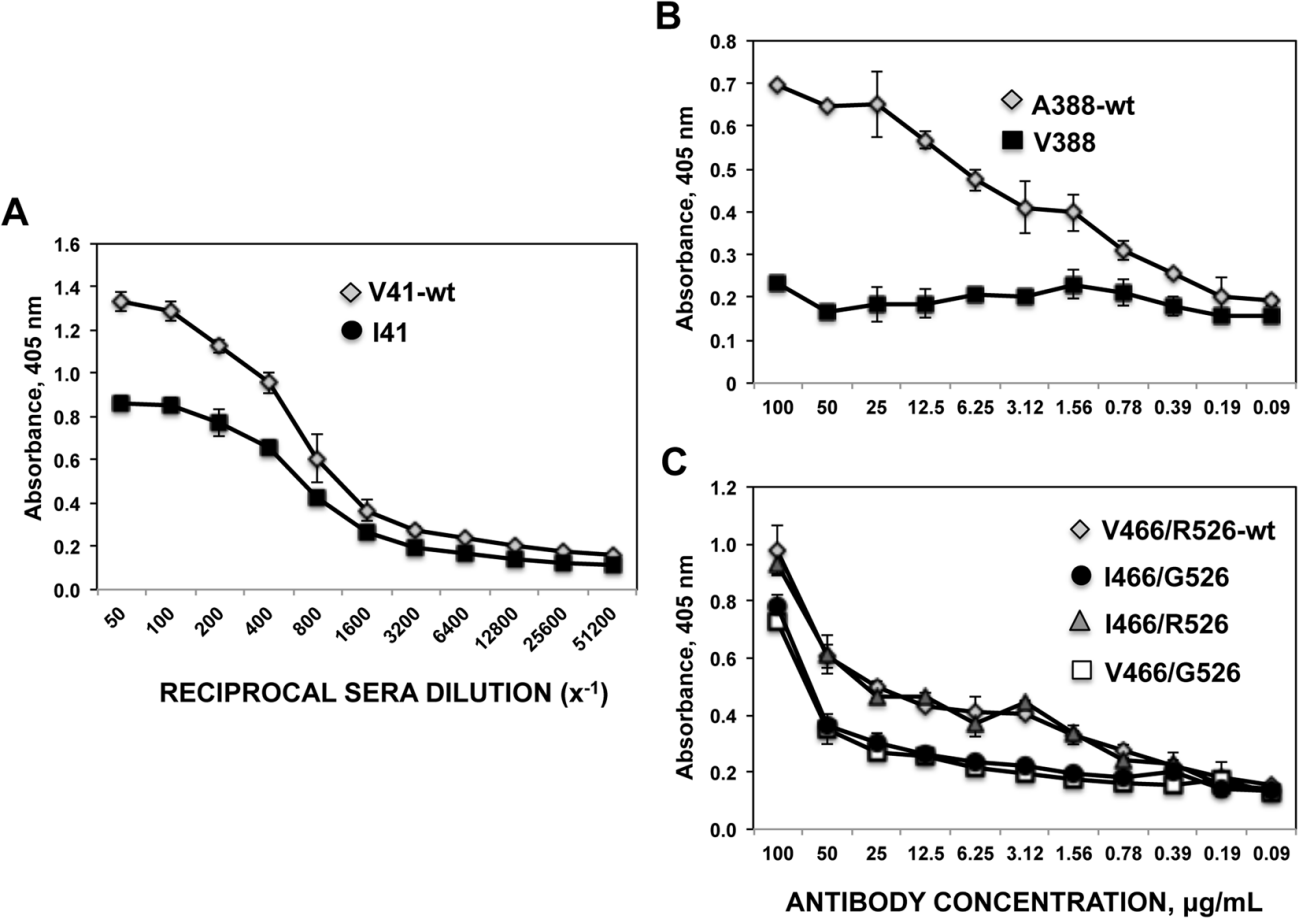
Effect of mutations V41I, A388V, and R526G on the binding of mAbs/sera to the virus. ELISA titers specific for the whole virus were measured using the sera from patient 298 pre-absorbed for anti-head antibodies (**A**), mAb 6F12 (**B**), and mAb CR9114 (**C**). Concentrated virions from the following recombinant viruses were used: WT and V41I (A), WT and A388V (**B**) and WT, V466I, R526G and V466I/R526G (**C**). ELISAs were performed twice in duplicates, with similar results. Means and standard deviations from the duplicates are shown.

To test for complete loss versus partial loss of mAb/sera binding, recombinant viruses from clarified supernatants of infected cells were concentrated, and ELISAs against the whole viruses were performed. Whereas the mutations A388V abolished the binding of mAb 6F12 to the virus, the mutations R526G and V41I decreased the binding of mAb CR9114 and of sera 298 pre-absorbed for anti-head antibodies, respectively, but they did not completely abolish the binding (Fig. 8).

The HA sequences deposited in IRD since the virus emerged in 2009 were analyzed. Interestingly, all the mutations selected in the HA protein head (S160G, A152S, T89A, and V237M) and stalk (V466I, V41I, R526G, A388V) domains, were detected in subjects infected with 2009 pandemic H1N1 viruses although at low frequencies (with the exception of mutation S160G) (Supplementary Figure 5A and B, Supplementary Table 4). Interestingly, R526G was present in 6 viruses from 2009, isolated in different geographical locations, and then disappeared from pH1N1 thereafter. These mutations were also found in seasonal H1N1 (Supplementary Table 4), with low frequencies, except the mutation R526G, which is found at very high frequency (98%). These data indicated that all the mutations lead to viable viruses already present in the human population.

### Effect of HA-stalk mutations on virus growth

**Figure 9:**
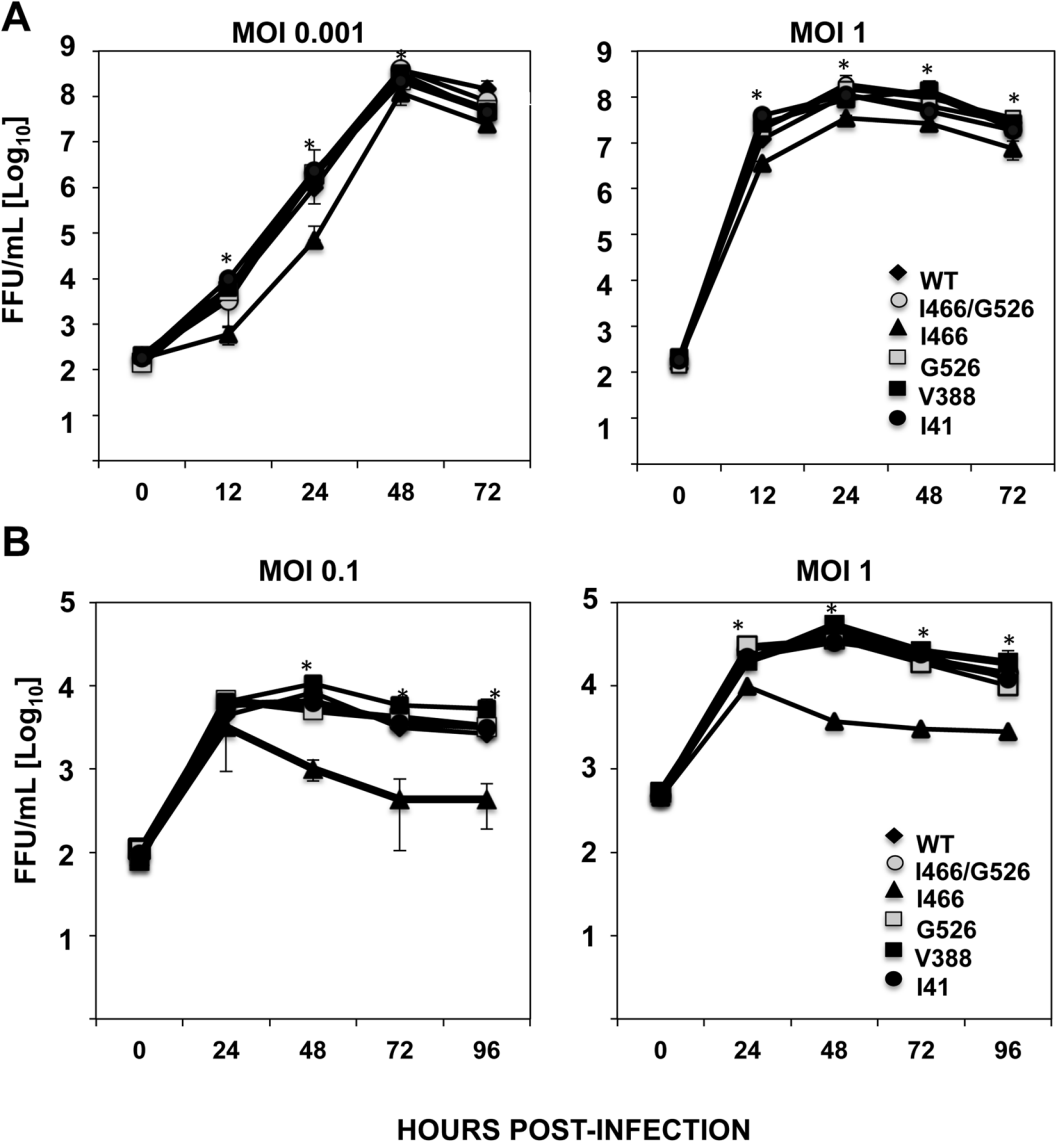
Recombinant viruses growth kinetics *in vitro*. Canine MDCK (**A**) and human A549 (**B**) cells were infected in duplicates with recombinant A/California/04/2009/E3 viruses incorporating the mutations in the HA protein stalk domain at the indicated MOIs. Virus titers of infected cells supernatants were determined at different times pi by immunofocus assay. Experiments were repeated three independent times in duplicate, with similar results. Data represents the results from the three experiments combined. *p-values <0.05 for comparison of wt versus I466 virus, using a Student’s t-test. P-values using the same Student’s t-test were >0.05 (not significant) for comparison of wt versus I466/G526, wt versus G526, wt versus V388, and wt versus I41 viruses.

To analyze whether the mutations selected in the HA stalk domain affect virus growth, virus titers of wt and recombinant viruses encoding the mutations selected (V388, I41, and I466/G526, separately or in combination) were compared, by infecting MDCK cells (MOIs 0.001 and 1, Fig. 9A) and human A549 cells (MOIs 0.1 and 1, Fig. 9B). In both cell lines, all the viruses, with the exception of mutant I466 (which grew with titers around 10-fold lower), grew to the same extent (Fig. 9A and B), suggesting that immune pressure could select mutations in the stalk domain which contribute to antibody evasion and do not compromise virus growth, at least *in vitro*.

### Effect of HA-stalk mutations on pathogenesis *in vivo*

**Figure 10:**
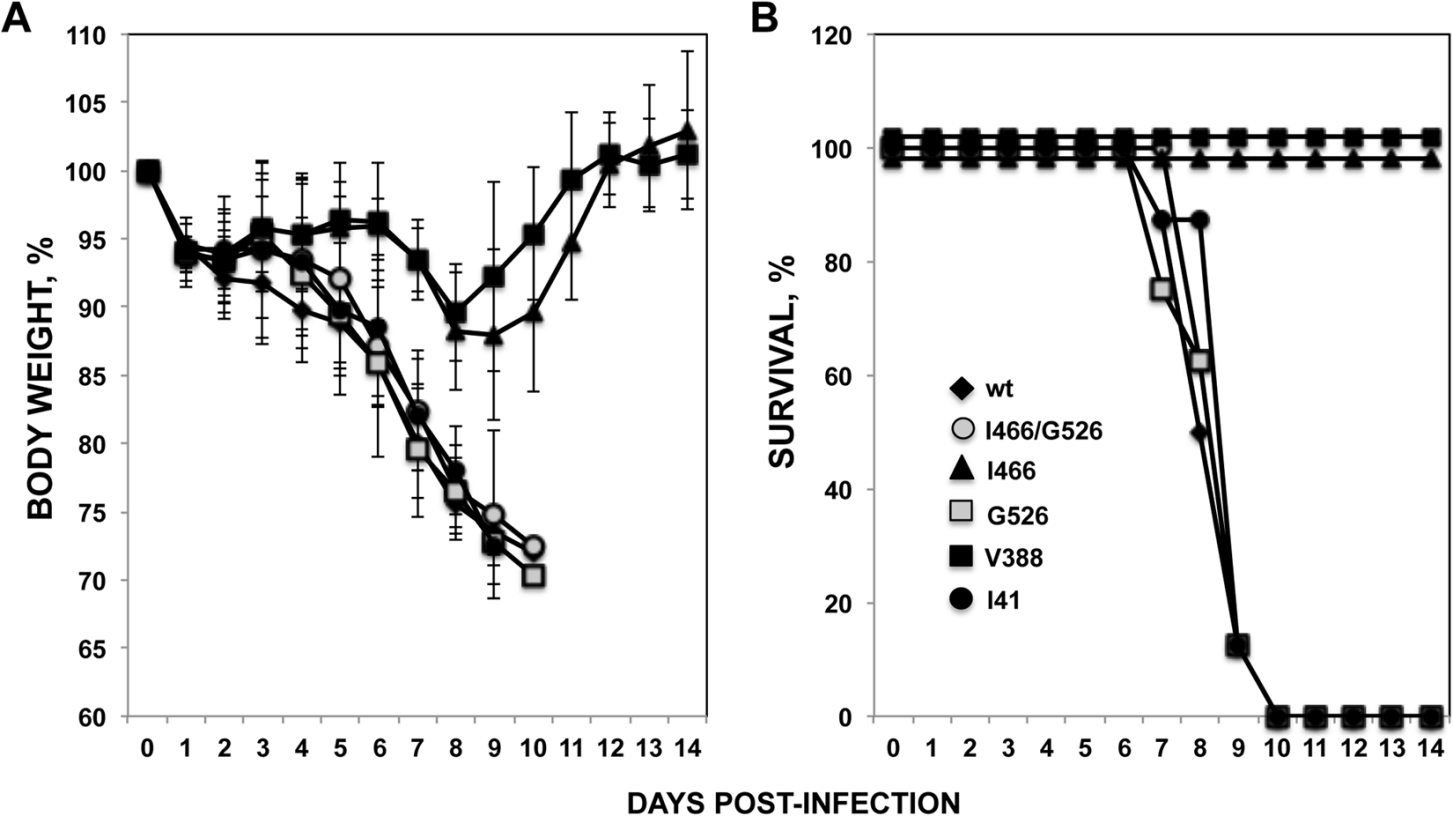
Virulence of recombinant A/California/04/2009/E3 viruses containing mutations in the HA stalk domain. Groups (N=8) of 7- to-8-week-old C57BL/6 female mice were infected (500 FFU/mice) with recombinant A/California/04/2009/E3 viruses containing mutations in the HA stalk domain. (**A**) Mice were monitored daily for weight loss. Data is plotted as the percent of the starting weight averaged for each group of mice. To determine significance, the weight loss over time (days) was non-parametrically modeled by penalized regression splines, assuming different curves for each virus. Bonferroni corrected confidence intervals for the A388V and V466I were significantly less than the other groups, including the wt virus (p < 0.003). (**B**) Mice survival was analyzed during 14 days.

Given that the viruses with mutations in the stalk domain grew well *in vitro*, we determined whether they would be equal in pathogenesis to the wt A/California/04/2009/E3 H1N1 virus *in vivo*. Groups of mice were infected with 500 FFU of virus. They were then monitored for weight loss, which is a sensitive measure of disease. All the mice lost weight within the first 8-10 days of infection (Fig. 10A). Mice infected with the V466I and A388V stalk mutations lost only 12% and 9% of their starting weight by day 8. However, viruses with the V41I, R526G or both V466I and R526G mutations were all highly virulent and similar to the wt influenza A/California/04/2009/E3 virus, causing all the animals to lose more than 25% of their weight (the limit of what they can tolerate) by day 9-10 (Fig. 10A). Considering that mice losing more than 25% of their initial weight had reached the experimental endpoint, survival curves were determined. Whereas all the mice infected with the mutant viruses V466I and A388V survived the infection, 100% of mice infected with the wt, V41I, R526G or both V466I/R526G viruses died (Fig. 10B). These observations demonstrate that while some stalk mutations can decrease viral pathogenesis (V466I and A388V), other viruses encoding mutations affecting the antigenicity of the stalk domain (V41I, R526G, and V466I/R526G) are fully viable, fit, and as virulent as the wt virus.

## Discussion

In this work, we have addressed whether the HA stalk domain can drift. First, we analyzed the sequences of human H1N1 influenza viruses circulating since 1918. We found sequence variability in the HA head, but also in the stalk domain (Figs. 1 and 2), indicating that the stalk domain is not absolutely conserved. Furthermore, when stalk sequence variation was evaluated over time, these aa changes appear to follow a non-random path to the present day and stalk codons demonstrate positive selection (dN/dS analysis, Fig. 2A). This suggests they occur as a result of a progressive biological effect such as immune pressure, though this is difficult to directly confirm. In support of this interpretation, ELISA data using different HA strains and different mAbs directed against the stalk domain showed differences in recognition (Fig. 3), suggesting antigenic variability. Clearly, experiments of this type would benefit from more comprehensive mAbs targeting different regions of the HA stalk domain.

To experimentally analyze HA protein antigenic drift, virus A/California/04/2009/E3 was passaged under immune pressure using immune sera and mAbs. The original virus used was selected in eggs in a single passage from a common human isolate collected early in the 2009 pandemic^51^, and encodes three mutations in the HA protein head domain (K136N, S200P, and D239G) as compared to the sequence in GenBank (reference FJ966082.1). Interestingly, mutations S200P, K136N or K136E, and D239G increased virus growth in eggs, MDCK cells and mice^51–53^, suggesting that these mutations were selected as a consequence of growing the virus in cell systems. Mutations in positions 136 and 239 did not significantly alter the antigenicity of the protein using immune ferret sera^53^.

For virus passages, we deliberately chose human sera from subjects infected and/or vaccinated with the pandemic A/California/04/2009 strain (Supplementary Table 3), as it has been shown that infection and even vaccination with the pH1N1 2009 virus induced higher levels of antibodies specific for the stalk domain compared to infection or vaccination with seasonal influenza viruses^8,17–21,23^. Our data show that growing the virus under immune pressure provided by different human sera, can select different mutations in the head and stalk regions, suggesting that individuals have different antibody repertoires and therefore, that different sera can provide different pressures depending on the region of HA bound by the antibodies. Accordingly, a study using sera from different patients revealed variations in the binding of antibodies to different regions of HA proteins^54^. Similarly, previous works showed that in the presence of different patient sera, the mutations selected in the HA protein head antigenic sites were not always the same^55,56^. These findings support the conclusion that it is easier to select mutations in the head than in the stalk domain using human sera, although this effect could vary in different people based on the quality or quantity of their anti-stalk-specific antibodies. In our work, mutations in the head were selected first at passage 4, whereas the earliest mutations in the stalk were not selected until passage 8 (Table 1). One explanation is that antibodies to the HA head domain in human sera are generally more abundant^57^, providing greater selection efficiency. Another reason for the lower selection efficiency in the HA stalk region could be the lower structural tolerances in the stalk domain compared to the head domain^37,38^. Furthermore, within the head and stalk domains, different tolerances exist: the receptor-binding domain on average has a significantly higher mutational tolerance than all sites in the protein, although sites in the receptor-binding pocket itself are often highly constrained. On the other hand, sites in the stalk fusion domain have a significantly lower mutational tolerance than all sites, and even the stalk epitopes for broadly neutralizing antibodies have a low mutational tolerance.

Not surprisingly, after growing the virus A/California/04/2009/E3 in the presence of human sera, the HA protein head domain mutations V237M, T89A, and S160G, located in the previously described antigenic sites Ca2, Cb, and Ca2, respectively^50^ were selected (Table 1). Consistent with these results, these mutations contributed to evasion of the antibody response, as determined in HAI and MN assays. Remarkably, a similar mutation to the T89A mutation selected in this work (T89K), was selected after passaging the virus A/California/04/2009 in the presence of a mouse mAb specific for this HA protein strain^58^, confirming the relevance of this residue in the binding to antibodies. By performing molecular dynamic simulations, it was suggested that the same mutation we found (S160G), also likely arose to avoid immune recognition^59^.

The human sera used in this study contained antibodies specific for the HA protein stalk domain (Supplementary Figure 3), consistent with the results showing that infection and even vaccination with the pandemic H1N1 2009 virus induced high levels of antibodies specific for this domain^17–21^. Interestingly, using one of these human sera a mutation in the stalk domain (V41I) that partially affected the ability of the serum to neutralize the virus (Figs. 5C,D, 6C,D and 7A) was selected, providing proof of concept that immune pressure from polyclonal human sera can cause antigenic drift in the stalk domain. This mutation (V41I) was located close to the fusion peptide of the stalk domain (Fig. 4), suggesting that the antibody selecting this mutation may be inhibiting the fusion of viral and endosomal membranes necessary to release the virus genome in the cytoplasm^13^.

The HA stalk domain-specific mAb 6F12 used in this study was obtained by sequentially immunizing mice with plasmid DNA encoding the HA of antigenically different H1 influenza A viruses and interestingly, has neutralizing activity against a divergent panel of H1 viruses *in vitro*^31^. However, using the mouse mAb 6F12, one mutation in the stalk domain, affecting the ability of the mAb 6F12 to neutralize A/California/04/2009/E3, was selected (A388V) after 8 passages (Figs 5A, 6A, 7B and Table 1) and in a previous report^31^. Nevertheless, in the previous report, the authors did not generate a recombinant virus expressing the mutated stalk to measure the microneutralization activity of this antibody to the escape mutant or report on the ability of the virus to cause disease *in vivo*, as we did herein. This mutation was positioned in the short α-helix at the interior interface of the stalk (Fig. 4)^31^. However, this position at the short α-helix seems inaccessible in the native pre-fusion conformation of HA, suggesting that either mAb 6F12 binds after conformational changes during the fusion process or that this escape mutation induces conformational changes in residues relevant for the mAb 6F12 binding.

The human mAb CR9114 specific for the HA protein stalk domain selected an A/California/04/2009/E3 virus with mutations V466I/R526G in the stalk domain after 16 passages in cell cultures (Table 1). This human mAb was obtained from display libraries constructed from human B cells of volunteers recently vaccinated with the seasonal influenza vaccine, neutralizes many influenza A viruses, and protected mice against challenges with lethal doses of influenza A and B viruses^46^. Engineering viruses by encoding the mutations separately or in combination we found that the mutation R526G specifically was responsible for partially decreasing the neutralization activity of this antibody (Fig. 6B). Crystal structures were determined for Fab CR9114 with HAs from H5, H3, and H7^46^, but not H1. However, these structures do not include aa 526 of HA. This position is 15 aa away from the last residue (510) included in X-ray crystallography of HA^60^. Studies of the transmembrane and linker domain of H2 HA (a group 1 HA) suggest residue 526 is part of the linker, and is located very close to the transmembrane region, but outside of it^61,62^. Assuming this is the same in H1 HA, this is an unusual location for an antibody epitope. Therefore, whether this aa directly affects the epitope to mAb CR9114, or it affects the conformational structure of the epitope, needs further investigation.

Interestingly, all the mutations selected in the HA protein head and stalk domains in this study have been found in A/California/04/2009 viruses circulating in the human population since 2009 (Supplementary Figure 5), suggesting that antigenic drift of the pH1N1 HA stalk may already be under way. According to this hypothesis, sera from contemporary cohorts frequently had cross-neutralizing antibodies to 2009 H1N1 and A/New Caledonia/20/1999 viruses, that mapped in the HA stalk domain. Interestingly, a conserved mutation in HA2 from strains that circulated in the 2006/07 and 2007/08 influenza seasons, respectively, abrogated this neutralization, suggesting that the stalk domain may be evolving under direct or indirect immune pressure^63^.

We have found that while two individual mutations attenuated the viruses *in vitro* (V466I) or *in vivo* (V466I and A388V), the other mutations in the stalk domain (V41I and R526G) did not significantly affect the growth of the viruses *in vitro*, or pathogenesis in mice (Figs 9 and 10). In contrast, in previous studies, all the escape mutations in the HA stalk domain selected in the presence of mAbs led to virus attenuation^32,64^.

Altogether, these data indicated that under immune pressure using mAbs, and remarkably, using sera from human subjects, fit viruses incorporating partial escape mutations in the HA protein stalk domain can be generated, though changes in the stalk epitopes occur more slowly *in vitro* and in nature than epitopes in the head. These results are important to inform the proposed use of “universal” influenza vaccines based on the assumption that the stalk domain is conserved and therefore not capable of antigenic drift.

## Materials and Methods

### Publically Available HA Sequences

DNA and protein sequences for human H1N1 hemagglutinin influenza viruses were compiled from the Influenza Resource Database (fludb.org) resulting in 14988 and 5565 sequences, respectively. Sequences were filtered by removing those with missing nucleotides or amino acids, duplications, and those without associated metadata; resulting in 11535 DNA and 4827 protein sequences. Sequences were aligned using the MUSCLE algorithm.

### Positive Selection Analysis

dN/dS ratios were calculated on aligned DNA sequences using the HyPhy MPI software. Analysis was performed using the HKY85 evolutionary model, single Ancestor Counting, and full tree SLAC. dN/dS ratio is used to infer the direction and magnitude of natural selection acting on a codon. Generally, dN/dS values above one indicate positive selection, less than one indicate negative selection, and equal to one neutral selection.

### Shannon Entropy

Shannon entropy (SE) of a multiple protein sequence alignment can be used to estimate diversity at each aa position and is less effected by sampling error and biases^65^. SE values greater than 2.0 are considered variable, those less than 2.0 are considered relatively conserved and values lower than 1.0 very conserved, being a value 0 when only one aa is present at that particular position. Shannon entropy was calculated on 4827 H1 protein sequences using the BioPhysConnectoR package in R.

### Principal Component Analysis (PCA)

Each HA sequence was truncated to include only amino acids positions 1–59 & 292–567, producing a string of amino acids representing the stalk^9^. All viruses with complete HA protein sequences were included (4827 sequences). Alignment was performed using the MUSCLE algorithm. Pairwise Hamming distances between all sequences were calculated resulting in a square distance matrix. In information theory, the Hamming distance between two strings of equal length is the number of positions at which the corresponding symbols are different. In other words, it measures the minimum number of substitutions required to change one string into the other. Principal component analysis, which clusters the sequences based on the positions that account for the most variation, was performed on the distance matrix using the FactoMineR package in R. After this dimensional reduction technique, the results can be plotted in two dimensions with each dimension accounting for a proportion of the variation in sequence^40,42^.

### 3D Protein Structure

HA protein structure was created from the A/California/04/2009 (H1N1) protein sequence using the automated protein structure homology-modeling server (swissmodel.expasy.org). Figure was created and labeled using the MacPyMOL software.

### Study Design and Human Subjects

Human subjects were enrolled as part of a prospective “family flu” surveillance study of families with at least one child in the household less than 4 years of age. Subjects reporting influenza-like illness (fever, cough, rhinitis) were asked to visit the University of Rochester Vaccine Research Unit (VRU) for sampling by nasal wash and nasopharyngeal swab (combined). Sera from subjects were obtained prior to the start of the flu season, and around 28 days after the acute illness visit. Families were followed for at least one week for evidence of infection. For this study, sera from subjects FAM195, FAM196, FAM203, FAM256, FAM297, FAM298, and FAM300 (named as 195, 196, 203, 256, 297, 298, and 300) collected at 2010 fall, were used.

### HA protein monoclonal antibodies (mAbs) and IgG isotype controls

Anti-HA stalk domain human IgG mAb CR9114^46^ was kindly provided by Patrick Wilson (University of Chicago, IL). Anti-HA stalk domain mouse IgG mAb 6F12^31^ was kindly provided by Peter Palese (Mount Sinai School of Medicine, NYC, NY). Anti-HA2 domain RA5-22 mouse mAb was obtained from Bei Resources (NR-44222). The mAbs C179 (human), F49 (human), CM2S3 or CR6261 (human), B198M (mouse), and FB75 (human), specifically recognizing the stalk region of the HA protein were obtained from Clontech (M145, and M146, respectively), from Acrobiosystems (CR1-M2S3), from GeneTex (GTX40926), and from Absolute Antibody (AB00148), respectively. A mAb specific for the head domain of HA A/California/04/2009 produced in mouse ascites fluid was obtained from BeiResources NIAID/NIH (NR-28665). Human and mouse IgG isotype controls were obtained from Thermo Fisher Scientific.

### Cells and viruses

Madin-Darby canine kidney (MDCK) cells, human embryonic kidney (293T) cells, and human lung epithelial carcinoma (A549) cells were obtained from the ATCC (CCL-34, CRL-11268, and CCL-185, respectively). All cell lines were grown in Dulbecco’s modified minimal essential medium (DMEM, Gibco) supplemented with 10% fetal bovine serum (Gibco) and 100 units/mL penicillin, 0.1 mg/mL streptomycin and 50 μg/mL gentamicin (Gibco). The influenza vaccine strain A/California/04/2009/E3 grown in eggs^49^ was used for the passages of the virus in the presence/absence of immune pressure.

### Virus titrations

Influenza viruses were titrated by immunofocus assay (Fluorescent Forming Units, FFU/mL) in MDCK cells. Confluent wells of MDCK cells (96-well format, 10^4^ cells/well) were infected with 10-fold serial dilutions of tissue culture supernatants. At 8 h post-infection (hpi), cells were fixed and permeabilized (4% formaldehyde 0.5% triton X100 in PBS) for 20 min at room temperature. After washing with PBS, the cells were incubated in blocking solution (2.5 % BSA, in PBS) for 1 h at room temperature, washed with PBS, and incubated with influenza virus NP mAb (Bei Resouces NR -4282) diluted in PBS/1% BSA for 2 h at 37 °C. After washing with PBS, the cells were incubated with a fluorescein isothiocyanate (FITC)-conjugated rabbit anti-mouse IgG secondary antibody (Dako) for 1 h at 37 °C. Cells were visualized using a fluorescence microscope. NP-expressing cells were enumerated to determine the virus titer (FFU/mL). All the infections were performed in the presence of 1 μg/mL of tosylsulfonyl phenylalanyl chloromethyl ketone (TPCK)-treated trypsin (Sigma).

### Virus passages

Virus A/California/04/2009/E3^49^ (MOI 0.1) was incubated during 1 h at RT with different 3-fold dilutions of sera from patients (1:50, 1:150, 1:450 and 1:1350 dilutions) or with different concentrations of mAbs 6F12 and CR9114 (0.2, 1, 5 and 10 μg/ml). As controls, viruses were passaged in the presence of human and mouse IgG isotype antibodies (at 10 μg/ml), in the presence of two patient sera showing HAI titers <10 (at a 1:50 dilution) or in the absence of immune pressure. Then, sera-virus mixtures were used to infect MDCK cells (24-well plate format). When cytopathic effect (CPE) was evident (approximately 24 hpi in the absence of sera/mAb, and at 48–72 hpi in the presence of sera/mAb), total RNA was collected from the wells incubated with the highest Ab/sera amounts, a RT-PCR was performed, and the PCR products were sequenced, using the protocol described above. Supernatants were collected and passaged in the presence of sera/mAbs, up to 16 times.

### HA and NA sequencing

RNA was obtained from cell culture extracts using the RNeasy mini kit (Qiagen) according to the manufacturer’s instructions. Reverse transcription reactions were performed during 2 h at 37 °C using the High-Capacity cDNA reverse transcription kit (Life Technologies), and the primers pH1N1-HA-5′-NCR-VS (5′-GGGGAAAACAAAAGCAACAAAAATG-3′) and pH1N1-HA-3′-NCR-RS (5′-GTGTTTTTCTCATGCTTCTGAAATCCTAAT-3′) for HA amplification, and the primers NAcal-1-VS (5′-ATGAATCCAAACCAAAAGATAATAACC-3′) and NAcal-1410-RS (5′-TTACTTGTCAATGGTAAATGGCAAC-3′), for NA amplification. For HA sequencing, the cDNAs were amplified in two HA overlapping regions by using the Pfx DNA Polymerase (Life Technologies), and the primers pH1N1-HA-5′-NCR-VS and pH1N1-HA-923-RS (5′-GGGAGGCTGGTGTTTATAGCACCC-3′) (for region 1), and pH 1N1-HA-793-VS (5′-CTAGTGGTACCGAGATATGCATTCGC-3′) and pH1N1-HA-3′-NCR-RS (for region 2). For NA sequencing, the cDNAs were amplified with the same primers used for RT reaction. The VS and RS primers used for each PCR were used for Sanger sequencing.

### Construction of plasmids pDZ encoding influenza HA proteins for virus rescue

HA proteins encoded by the viruses A/California/04/2009/E3 and the different variants containing mutations in the HA stalk domain, were cloned in pDZ plasmids using the restriction enzyme SapI. To generate the plasmids encoding the wild type (wt)-HA, the V41I-HA, the A388V-HA, and the V466I+R526G-HA, the HA segments were amplified using RNAs from infected cells. To this end and to delete one SapI restriction enzyme site by introducing one silent mutation, overlapping PCRs were performed using the primers SapI-5′NCR-HA-VS (5′- GATC*GCTCTTC*TGGGAGCAAA AGCAGGGGAAAATAAAAGCAACAAAAATGAAGGCAATACTAGTAGTTCTGC-3′, including an SapI restriction site in italics, the 5′ NCR and first 25 nt of HA ORF), and ΔSapI-HA-1162-RS (5′-CATTCTGTGTGCTCTT*A*AGGTCGGCTGCATATC-3′, complementary to nucleotides 1130 to 1162 of HA ORF, and including one silent mutation to delete SapI restriction site in italics) for the 5′ PCR, and the oligonucleotides ΔSapI-HA-1130-VS (5′-GATATGCAGCCGACCT*T*AAGAGCACACAGAATG -3′, complementary to nucleotides 1130 to 1162 of HA ORF, and including one silent mutation to delete SapI restriction site in italics) and SapI-3′NCR-HA-RS (5′-GATC*GCTCTTC*TATTAGTAGAAACAAGGGTGTTTTTCTCATGCTTTCTGAAATCCTAATGTTAAATACATATTCTACACTG-3′, including an SapI restriction site in italics, the 3′ NCR and last 21 nt of HA ORF) for the 3′ PCR.

To generate the plasmid encoding the V466I-HA, the plasmids pDZ-wt-HA and the plasmid pDZ-V466I+R526G-HA were digested with the restriction enzymes BstBI (cutting at nt 775 of the HA ORF), and PmlI (cutting at nt 1470 of the HA ORF), and the HA fragments were exchanged. To generate the plasmid encoding the R526G-HA, the plasmids pDZ-wt-HA and the plasmid pDZ-V466I+R526G-HA were digested with the restriction enzymes ClaI (cutting at nt 337 of HA ORF), and PmlI (cutting at nt 1470 of HA ORF), and the HA fragments were exchanged.

### Virus rescue

Cocultures (1:1) of 293T/MDCK cells in suspension were cotransfected, using DNA-IN (Molecular Transfec, Inc), with 1 μg each of the seven ambisense wt plasmids (pDZ-PB2, -PB1, -PA, -NP, -NA, -M, -NS) encoding A/California/04/2009 viral proteins (kindly provided by Adolfo Garcia-Sastre, Mount Sinai School of Medicine, NYC, NY) plus the ambisense HA plasmids (pDZ-HAs). At 12 hpt, the medium was replaced with DMEM containing 0.3% bovine serum albumin (BSA), antibiotics and 1 μg/mL TPCK-treated trypsin (Sigma). At 96 hpt, tissue culture supernatants were collected, clarified, and used to infect fresh MDCK cells. At 3 days post-infection (dpi), recombinant viruses were plaque purified and scaled up in MDCK cells. Virus stocks were generated by infecting confluent 10-cm dishes of MDCK cells at a low MOI (0.001). Stocks were titrated by immunofocus assay (FFU/mL) on MDCK cells, and the identity of the HA gene was confirmed by restriction analysis and sequencing.

### Recombinant proteins

Recombinant HA proteins from viruses A/California/04/2009 virus (H1N1), A/Solomon Islands/3/2006 (H1N1), A/South Carolina/11/1918 (H1N1), A/Brisbane/59/2007 (H1N1) were obtained from Influenza Reagent Resources (catalog numbers FR-180, FR-67, FR-692, and FR-65, respectively). Recombinant HA proteins from viruses A/Hong Kong/33982/2009 (H9N2), A/Singapore/1/1957 (H2N2), A/Brisbane/10/2007 (H3N2), A/New Caledonia/20/1999 (H1N1), B/Brisbane/60/2008, and A/Puerto Rico/8/1934 (H1N1), were obtained from Bei Resources (catalog numbers NR-41792, NR-2668, NR-19238, NR-48873, NR-19239, and NR-19240, respectively).

A recombinant protein expressing the H6 head domain from A/mallard/Sweden/81/2002 (H6) and the stalk region from A/California/04/2009 (cH6/H1) was kindly provided by Florian Krammer (Icahn School of Medicine at Mount Sinai, New York). Recombinant protein expressing the H5 head ectodomain from A/Indonesia/05/2005 (H5) and the stalk region from A/California/04/2009 (cH5/H1) was obtained and purified in our lab. A recombinant protein expressing the ectodomain from A/Indonesia/05/2005 (H5) was obtained and purified in our lab, as previously described. Briefly, the extra-cellular domain of HA was fused to the FoldOn domain of T4 fibritin to promote trimerization and to the hexahistidine tag to facilitate affinity purification. The HA sequence included a Y98F mutation that prevents nonspecific binding to sialic acid. DNA plasmids were purified using an endotoxin-free maxi-kit (Omega) and transfected into 293F cells (Invitrogen) at a density of 1 × 10^6^ cells/mL grown with FreeStyle media (Invitrogen). The supernatant was collected after 4 days of transfection, cleared by centrifugation and passed through a Ni Sepharose Fast Flow column (GE Healthcare life Sciences). Protein was eluted with 500 mM imidazole and buffer exchanged and concentrated with Amicon 100 KDa (Millipore) at 4 ºC. The concentrated protein was purified by HPLC on a Superdex 200 16/60 column (GE Healthcare life Sciences) to collect the HA trimer. Integrity of the protein was confirmed by 10% acrylamide SDS-PAGE (BioRad) followed by Coomassie Blue staining.

### Enzyme-linked immunosorbent assays (ELISA)

96-well plates were coated with 500 ng per well of recombinant purified HA proteins, at 4 °C during 16 h. Alternatively, plates were coated with concentrated viruses. To this end, clarified supernatants from infected cells were concentrated 100X by treating them with a polyethylene glycol virus precipitation solution (System Biosciences). Then, protein concentration was quantified and each well was coated with 50 μl of a 10 μg/mL solution. After washing with PBS containing 0.1% Tween 20, the coated wells were blocked with PBS containing 2.5% BSA, and then the plates were incubated with 1:2 dilutions of human serum (starting dilution, 1:10) for 2 h at 37 °C, or with mAbs (15 μg/mL) overnight at 4 °C. Then, the wells were washed with PBS containing 0.1% Tween 20, and incubated with alkaline phosphatase-conjugated goat anti-human IgG (Southern Biotechnology) for 30 min at 37 °C. The reactions were developed with p-nitrophenyl phosphate substrate tablets (Sigma) in diethanolamine buffer for 30 min at room temperature, and read at 405 nm (Vmax kinetic microplate reader; Molecular Devices).

### Hemagglutination inhibition (HAI) and virus microneutralization (MN) assays

For HAI assays, 2-fold dilutions of human sera treated with receptor destroying enzyme (RDE, Denka Seiken) were mixed with 8 HAU/50 μL of each virus. The mixtures were incubated with 0.5% turkey red blood cells (RBCs, Lampire Biological Laboratories), during 60 minutes to allow hemagglutination of RBCs. The HAI assays were performed in the presence of 20nM oseltamivir carboxylate. The HAI titer was defined as the highest dilution of serum that inhibits the hemagglutination of RBCs.

For MN assays to test the relevance of the HA stalk mutations, 2-fold dilutions of human sera or mAbs CR9114 and 6F12 were mixed with 1000 FFU of each virus, and incubated during 30 min at room temperature to allow the binding of the antibodies to the viruses. Serum from patient 298 was used directly, or was pre-absorbed with a recombinant A/California/07/2009 HA1 domain (Influenza Reagent Resources, FR-695). As control, mAb specific for the HA head domain of A/California/04/2009 (NR-28665, BeiResources, NIAID, NIH) was used. The serum-virus samples were then transferred to MDCK cells in M96 plate wells. After an absorption period of 60 min, the inocula were removed, and DMEM containing 0.3% BSA and 1 μg/mL of TPCK-treated trypsin (Sigma) supplemented with the sera or mAbs was added to the cultures. 2 days post-infection, virus supernatants were titrated using the immunofocus assay described above. Alternatively, to test the relevance of the HA head mutations, 2-fold dilutions of human sera were mixed with 1000 FFU of each virus, and incubated during 30 min at room temperature to allow the binding of the antibodies to the viruses. The serum-virus samples were then transferred to MDCK cells in M96 plate wells. After an absorption period of 60 min, the inocula were removed, and DMEM containing 0.3% BSA and 1 μg/mL of TPCK-treated trypsin (Sigma) was added to the cultures. 2 days post-infection, virus supernatants were titrated using an HA assay. The highest dilution of sera that completely abolishes virus growth is shown.

### Competition assays

Confluent MDCK cells were co-infected with the recombinant wt virus plus the I41, V388 or I466/G526 viruses, at MOIs of 0.01 and 0.005, respectively, in the absence or in the presence of serum from patient 298, pre-absorbed with a recombinant A/California/07/2009 HA1 domain (Influenza Reagent Resources, FR-695) (diluted 1:50 and 1:100 in the culture media), in the absence or presence of mAb 6F12 (at 10 and 50 μg/ml), or in the absence or presence or mAb CR9114 (at 5 and 25 μg/ml). When the CPE in the infected-cells was approximately 10% (12–24 h earlier in the cells infected in the absence of immune pressure), the total RNA from infected-cells was extracted, using the RNeasy mini kit (Qiagen) according to the manufacturer’s instructions, and amplified by RT-PCR as indicated above. PCR products were subjected to Sanger sequencing (Genewiz).

### Plaque reduction assays

50 Plaque Forming Units (PFU) of virus was incubated during 1 h at RT with 3-fold dilutions of mAb 6F12, mAb CR9114 or 298 serum pre-absorbed for antibodies specific for the HA1 domain (in duplicates). Then, this mixture was used to infected MDCK cells (24-well plate format), in the presence of the same concentration of mAb/serum, in DMEM media containing 1 μg/ml TPCK-trypsin, 0.3% BSA and 0.6% agar. 60 hpi, the cells were fixed with 10% formaldehyde and stained with crystal violet. Lysis plaques were counted from duplicate wells.

### Virus growth kinetics

To determine *in vitro* virus growth rates, confluent MDCK or A549 cells were infected in duplicates at an MOI of 0.001 or 1 (MDCK) or 0.1 and 1 (A549 cells). After 1 h of virus absorption at room temperature, cells were washed and overlaid with DMEM containing 0.3% BSA TPCK-treated trypsin (1 μg/mL for MDCK cells and 0.25 μg/mL for A549 cells). At the indicated times post-infection, tissue culture supernatants were collected and viral titers were determined by immunofocus assay (FFU/mL) as described above.

### *In vivo* pathogenesis

C57BL/6 female mice (Jackson Laboratories, Bar Harbor, ME) were sedated with avertin (2,2,2-tribromoethanol) and infected by instillation of 500 FFU of virus in 30 µl phosphate buffered saline intranasally. Individual animal weights were collected daily as a measure of disease severity. Mice showing 25% loss of their initial body weight were considered to have reached the experimental endpoint.

### Ethics statement

The study involving human subjects was approved by the University of Rochester Human Research Subjects Review Board (protocol number DMID 07-0046). The study was carried out in accordance with Good Clinical Practice (GCP) as required by the U.S. Code of Federal Regulations applicable to clinical studies (45 CFR 46), the International Commission on Harmonization (ICH) guidelines for Good Clinical Practice (GCP) E6, and the NIH Clinical Terms of Award. Investigators will complete and remain current with appropriate Human Subjects Protection Training. Informed written individual or parental consent was obtained for each participant.

All mice studies were conducted in an AAALAC certified vivarium under an Institutional Animal Care and Use Committee approved protocol. All experimental protocols were performed in accordance with the standards established by the United States Animal Welfare Act, as set forth by the National Institutes of Health guidelines. The experimental protocols were reviewed and approved by the University Committee for Animal Resources, number 101431-UCAR-2006-029R, and conducted in Association for Assessment and Accreditation of Laboratory Animal Care International accredited facilities.

### Author summary

Influenza viruses are considered an important human health problem, because they cause high morbidity and mortality. Despite efforts to vaccinate, estimates of protection range from 40 to 70% in the US, at least in part due to antigenic drift in the influenza HA head domain, requiring periodic updates to the seasonal vaccine to maintain a good match with circulating viruses. Because of this concern, antibodies against the more conserved HA stalk domain are currently being discussed as promising therapeutic targets. However, little work has been done analyzing influenza HA stalk antigenic variability and antigenic drift under immune pressure. We found antigenic variability in the HA stalk domain, suggesting antigenic drift. Furthermore, using human immune sera from pandemic A/California/04/2009 immune subjects and mAbs specific for the stalk domain, viruses containing mutations in both stalk and head domains that limitedly contributed to antibody evasion, were selected *in vitro*. Interestingly, recombinant viruses encoding two different amino acid changes in the HA stalk domain selected *in vitro*, retained pathogenicity *in vivo*. These data demonstrates that the HA protein stalk domain can be induced to drift under immune pressure, and the selected viruses can retain fitness and virulence in vivo, being a concern to consider in the design of vaccines targeting this domain

## Electronic supplementary material

Supplementary Tables
