# Publisher Correction: Natural and directed antigenic drift of the H1 influenza virus hemagglutinin stalk domain

**DOI:** 10.1038/s41598-017-17926-6

**Published:** 2018-01-05

**Authors:** Christopher S. Anderson, Sandra Ortega, Francisco A. Chaves, Amelia M. Clark, Hongmei Yang, David J. Topham, Marta L. DeDiego

**Affiliations:** 1 David H. Smith Center for Vaccine Biology and Immunology, and Department of Microbiology and Immunology, Rochester, NY United States; 2 Department of Biostatistics and Computational Biology, University of Rochester Medical Center, Rochester, NY 14642 United States

Correction to: *Scientific Reports* https://doi.org/10.1038/s41598-017-14931-7, published online 03 November 2017

The original HTML version of this Article contained a typographical error in the publication date ‘03 November 2017’ which was incorrectly given as ‘06 November 2017’. This has now been corrected in the HTML version of the Article.

