# Publisher Correction: Natural and directed antigenic drift of the H1 influenza virus hemagglutinin stalk domain

**DOI:** 10.1038/s41598-018-22372-z

**Published:** 2018-03-06

**Authors:** Christopher S. Anderson, Sandra Ortega, Francisco A. Chaves, Amelia M. Clark, Hongmei Yang, David J. Topham, Marta L. DeDiego

**Affiliations:** 1 David H. Smith Center for Vaccine Biology and Immunology, and Department of Microbiology and Immunology, Rochester, NY United States; 2 Department of Biostatistics and Computational Biology, University of Rochester Medical Center, Rochester, NY 14642 United States

Correction to: *Scientific Reports* 10.1038/s41598-017-14931-7, published online 03 November 2017

An error occurred after publication resulting in the inadvertent omission of the Article and Volume numbers from the PDF version of this Article. This error has now been corrected in the PDF version of the Article; the HTML version was correct from the time of publication.

