# Supplementary material for: Natural and directed antigenic drift of the H1 influenza virus hemagglutinin stalk domain
Source: Sci Rep. 2017 Nov 3;7:14614. doi: 10.1038/s41598-017-14931-7 (PMC5668287; doi:10.1038/s41598-017-14931-7)
Supplement: Supplementary file 1 — Supplementary Tables [file 41598_2017_14931_MOESM1_ESM.pdf]

Natural and directed antigenic drift of the H1 influenza virus hemagglutinin stalk domain

Christopher S. Anderson[1], Sandra Ortega[1], Francisco A. Chaves[1], Amelia M. Clark[1], Hongmei Yang[2], David J. Topham[1]*, and Marta L. DeDiego[1*].

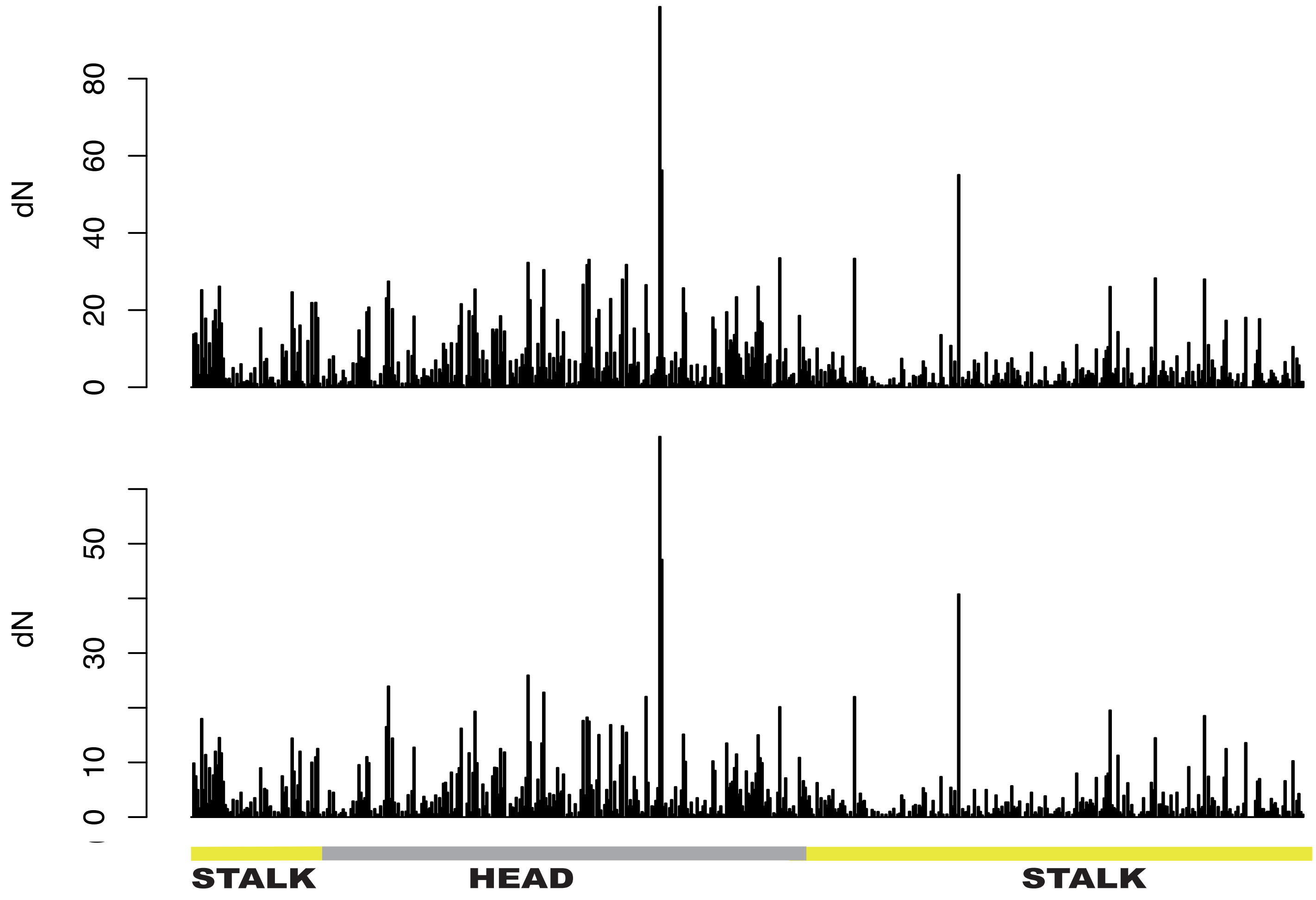

Supplementary Figure 1. Non-synonymous changes of influenza H1N1 HA protein. dN analysis of codons across the H1 HA proteins of viruses that circulated since 1918 (top panel) and pandemic viruses circulating since 2009 (bottom panel).

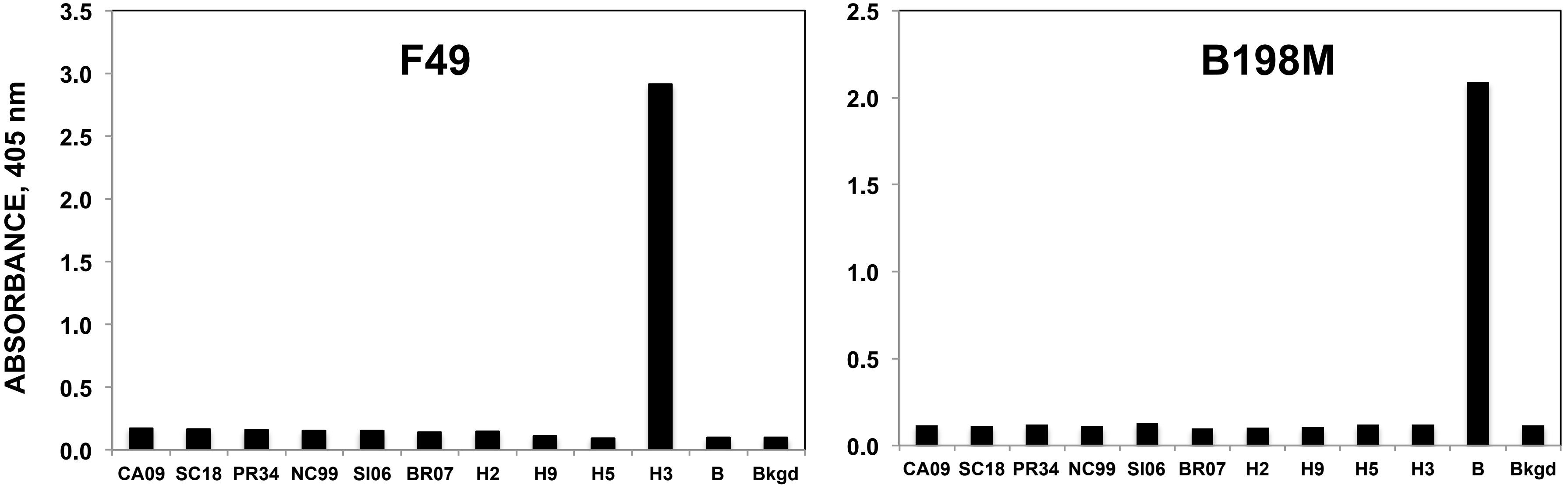

Supplementary Figure 2. Antigenic variation in the stalk region of historical influenza viruses. ELISA titers were measured using the monoclonal antibodies reactive to the stalk region of HA protein F49 (left panel) and B198M (right panel), against recombinant HA from 6 H1N1 viruses and 5 other subtypes (H2, H9, H5, H3, and influenza B). The assays were performed in duplicates, twice, and the averages are shown. Purified HA proteins from the following strains were used: H1N1 strains A/California/04/2009 (CA09), A/South Carolina/11/1918 (SC18), A/Puerto Rico/8/1934 (PR34), A/New Caledonia/20/1999 (NC99), A/Solomon Islands/3/2006 (SI06), A/Brisbane/59/2007 (BR07), and H2N2 A/Singapore/1/1957 (H2), H9N2 A/Hong Kong/33982/2009 (H9), A/Indonesia/05/2005 (H5), H3N2 A/Brisbane/10/2007 (H3), and B/Brisbane/60/2008 (B). Bkgd, background.

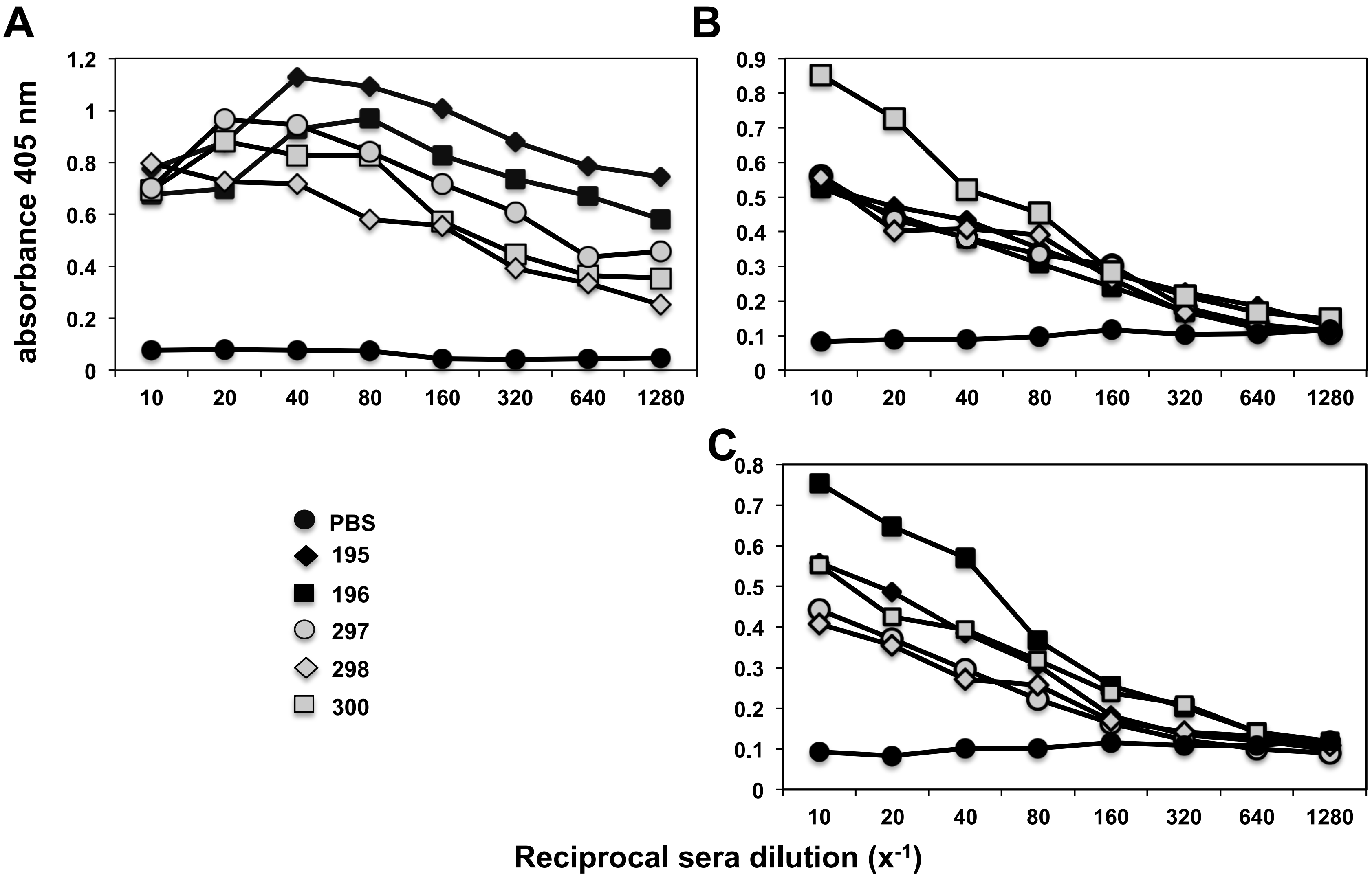

Supplementary Figure 3. Humoral responses in human sera after influenza A/California/04/2009 infection/ vaccination. Sera from patients 195, 196, 297, 298, and 300 were evaluated by ELISA for IgG antibodies specific for A/California/04/2009 HA protein (A) specific for a chimeric protein encoding the HA head from the "exotic" H5 strain A/Indonesia/5/2005 and the HA stalk from A/California/04/2009 strain (B) and specific for a chimeric protein encoding the HA head from the "exotic" H6 strain A/ A/mallard/Sweden/81/2002 and the HA stalk from A/California/04/2009 strain (C). Experiments were repeated twice, with similar results.

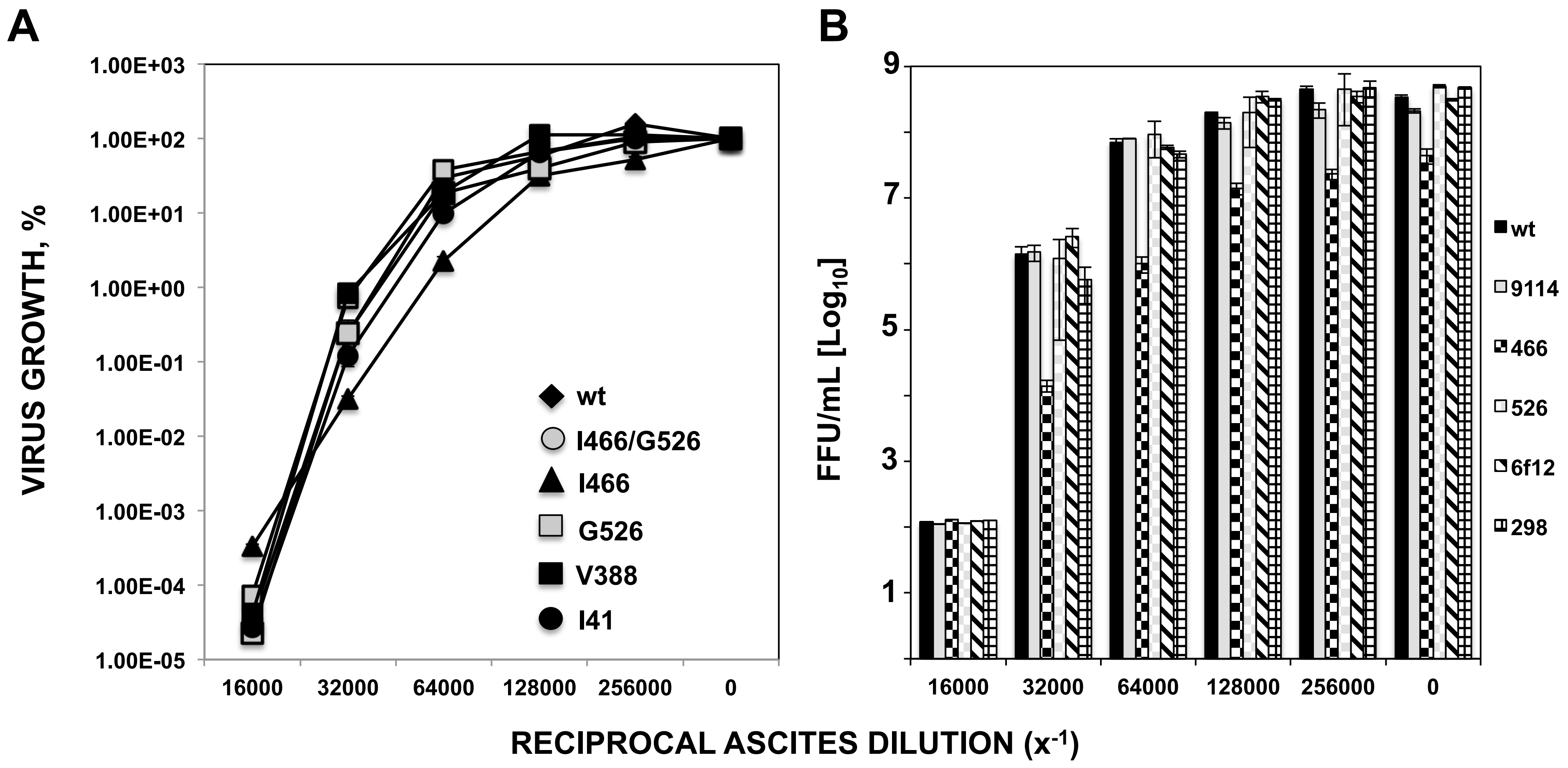

Supplementary Figure 4. Microneutralization assays for the recombinant viruses incorporating the stalk mutations A388V, V466I, R526G, and V41I. The wt virus or the viruses incorporating the mutations, were grown in the presence of different concentrations of the mouse ascites containing the anti-HA head antibody NR-28665. Virus growth in the presence of the mAb was compared to virus growth in the absence of mAb, and represented as % growth (A) or as virus titers (in FFU/ml, B). Experiments were repeated three independent times in duplicate, with similar results.

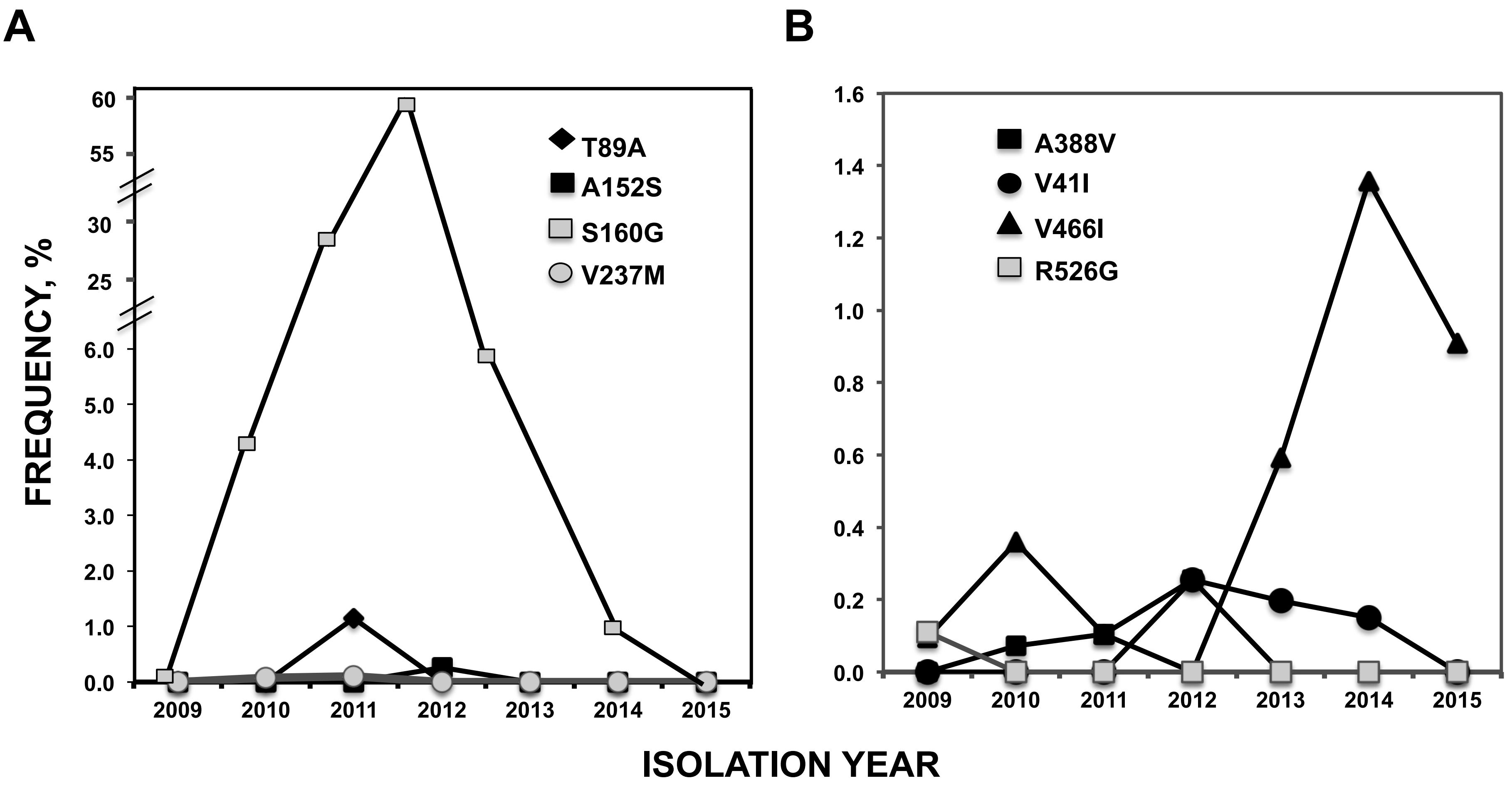

Supplementary Figure 5. Frequency of the HA protein mutations selected in human subjects infected with A/California/04/2009-like viruses since 2009. Analysis of HA protein sequence variability among the pandemic H1N1 strains publically available in Influenza Resources Database. The frequency of the mutations T89A, A152S, S160G, and V237M in the HA head domain (A), and the mutations V41I, A388V, V466I, and R526G in the HA stalk domain (B), per year of isolation, is represented.

SUPPLEMENTARY TABLE 1. dN/dS analysis showing selection of codons across the H1 HA proteins of viruses circulating since 1918.

	Observed S Changes	Observed NS Changes	E[S Sites]	E[NS Sites]	Observed S. Prop.	P{S}	dS	dN	dN-dS	P{S leq. observed}	P{S geq. observed}	Scaled dN-dS	dN_dS
1	0	0	0	3	0	0	0	0	0	0	0	0	0
2	18	27.9760702	0.850553424	2.034743766	0.391508015	0.294788844	21.16269184	13.74918585	-7.413505987	0.942137932	0.102815637	-0.763506313	1.554226122
3	3	28	0.999921804	1.99990717	0.096774194	0.33332627	3.000234607	14.00064984	11.00041523	0.002415603	0.99953834	1.132916934	9.333333333
4	0	29.99819361	0.253965927	2.735899464	0	0.084942261	0	10.96465495	10.96465495	0.069746848	0.930253152	1.129234034	0
5	15	4.000021701	1.760423586	1.23490525	0.789472782	0.587722979	8.52067657	3.239132478	-5.281544091	0.982187174	0.056426026	-0.543938625	0.266668113
6	6	49.01207032	1.048737086	1.944523544	0.109066973	0.350366111	5.721166991	25.20518225	19.48401526	4.49E-05	0.999990479	2.006630692	8.168678387
7	16	15	0.999332821	2.000667179	0.516129032	0.33311094	16.01068198	7.497498915	-8.513183068	0.988999313	0.02685432	-0.876760473	0.9375
8	27.05968426	23.00020057	1.708403638	1.289397559	0.540546275	0.569885568	15.83916333	17.83794332	1.998779984	0.389041406	0.713899359	0.205851474	0.849980375
9	24.0042025	4.000109321	1.733613082	1.249496889	0.857160949	0.581142867	13.84634366	3.201375974	-10.64496768	0.999636612	0.00175231	-1.096309903	0.166642042
10	44	23	0.768493637	2.009137178	0.656716418	0.276672347	57.2548657	11.44770016	-45.80716554	1	1.07E-10	-4.717614058	0.522727273
11	7	10	0.999458508	2.000495454	0.411764706	0.333157949	7.003792499	4.998761672	-2.005030827	0.82854872	0.325499254	-0.206495239	1.428571429
12	12	38.0172534	0.78083526	2.218757101	0.239917212	0.260313791	15.36815845	17.13448191	1.766323457	0.443580669	0.680516678	0.181911111	3.16810445
13	8.000316818	40.00099851	0.999904324	1.996878499	0.1666687	0.333659255	8.001082332	20.03176385	12.03068151	0.008048272	0.997092911	1.239022576	4.999926805
14	45.0035422	30.00662411	0.999535049	2.000464951	0.599965903	0.33317835	45.02447639	14.99982496	-30.02465143	0.999999363	2.00E-06	-3.092195643	0.666761385
15	11	52.0110302	1.009400431	1.990543305	0.17457261	0.336473121	10.89755825	26.12906239	15.23150414	0.003405548	0.998690862	1.568670692	4.728275473
16	8	37.00152676	0.76966941	2.227365049	0.177771746	0.25681033	10.39407296	16.61224179	6.218168835	0.147580059	0.921774237	0.64040026	4.625190845
17	14.00058702	15.00071738	1	1.999916748	0.482757149	0.333342584	14.00058702	7.500670912	-6.499916108	0.968850303	0.068187912	-0.669417006	1.071434888
18	3	5.000065045	0.768576834	2.231411964	0.374996951	0.256193235	3.903318273	2.240762856	-1.662555417	0.877431863	0.336953698	-0.17122419	1.666688348
19	13.00962081	4	0.999597715	2.000381085	0.764838967	0.333201593	13.01485649	1.999618987	-11.0152375	0.999953478	0.00034037	-1.134443459	0.307464765
20	18	3	1.464883826	1.327398795	0.857142857	0.524618753	12.28766383	2.260059306	-10.02760452	0.999748197	0.001546495	-1.032728559	0.166666667
21	14	2	0.768557138	2.086494268	0.875	0.269192049	18.21595208	0.958545648	-17.25740643	0.999999966	7.06E-07	-1.777315454	0.142857143
22	1	14.00015192	0.214261257	2.785721039	0.066665991	0.07142084	4.667199352	5.025683379	0.358484027	0.708710738	0.670936967	0.036919754	14.00015192
23	11	0.000195899	0.999999999	1.999988814	0.999982191	0.333334576	11.00000002	9.80E-05	-10.99990207	1	5.65E-06	-1.132864084	0.000017809
24	15	7.00053737	0.768493637	2.000154858	0.681801528	0.277569955	19.51870422	3.499997683	-16.01870653	0.999984831	9.35E-05	-1.649743534	0.466702491
25	4	2.000065039	0.768502762	2.231450579	0.66665944	0.256171572	5.204925992	0.896307119	-4.308618873	0.994793427	0.040948586	-0.443738457	0.50001626
26	36.00909565	12.00035049	1	2	0.750041889	0.333333333	36.00909565	6.000175243	-30.0089204	0.999999999	4.26E-09	-3.090575527	0.333258869
27	8.000541947	3	0.768600017	2.231399983	0.727286163	0.256200006	10.40923988	1.344447442	-9.064792439	0.999844986	0.001415352	-0.933569928	0.374974598
28	15	3.000021783	0.768493637	2.231506363	0.833332325	0.256164546	19.51870422	1.344393112	-18.17431111	0.99999997	4.81E-07	-1.871746146	0.200001452
29	6	3.000238591	1.000017357	1.77328363	0.666648994	0.360587387	5.999895859	1.691911288	-4.30798457	0.986582654	0.061694637	-0.443673131	0.500039765
30	16.00373411	3	1	2	0.842136288	0.333333333	16.00373411	1.5	-14.50373411	0.999999376	7.30E-06	-1.493718704	0.187456251
31	6	8	0.76855363	2.23141278	0.428571429	0.256187412	7.806872244	3.585172619	-4.221699626	0.956661974	0.122882541	-0.434786769	1.333333333
32	16	2	0.999997376	2.000002624	0.888888889	0.333332459	16.00004198	0.999998688	-15.00004329	0.999999905	1.68E-06	-1.544832872	0.125
33	16.0010237	10	0.999317087	2.000671729	0.615399758	0.333106937	16.01195848	4.998321242	-11.01363723	0.999180766	0.002942745	-1.134278649	0.624960014
34	8.002189465	2.000086897	0.768500662	2.231499338	0.800036829	0.256166887	10.41272944	0.896297329	-9.516432107	0.999963389	0.000498554	-0.980083648	0.249942457
35	13	0	1	2	1	0.333333333	13	0	-13	1	6.27E-07	-1.338851292	0
36	24	31	0.975379172	2.024192006	0.436363636	0.325172871	24.60581554	15.3147527	-9.29106284	0.969285572	0.055355815	-0.956873191	1.291666667
37	25	1.000065377	1.727636859	1.271948184	0.961536044	0.575958619	14.47063361	0.786246947	-13.68438666	0.999999411	1.19E-05	-1.409335289	0.040002615
38	12	14	0.8065703	2.119738897	0.461538462	0.275627162	14.8778104	6.604587016	-8.27322338	0.98752914	0.03280417	-0.85204737	1.166666667

39	11	15.00010903	0.852887929	2.031391726	0.423075149	0.295702231	12.89735688	7.384153847	-5.513203034	0.945457906	0.11531609	-0.567796847	1.363646275
40	7	4	0.76850206	2.231469668	0.636363636	0.256169768	9.108628802	1.79254061	-7.316088192	0.998585869	0.008727279	-0.753473394	0.571428571
41	17.00435933	5	0.999829261	2.000170739	0.772772298	0.33327642	17.00726313	2.499786595	-14.50747653	0.999995852	3.10E-05	-1.49410413	0.294042245
42	8	5.000130196	0.999902501	2.000097499	0.615378452	0.333300834	8.00078007	2.499943227	-5.500836844	0.991182256	0.0346372	-0.56652327	0.625016275
43	18.00527012	2.000021802	0.999996307	1.999975539	0.900025363	0.33333523	18.00533662	1.000023132	-17.00531349	0.999999988	2.31E-07	-1.751352764	0.1110798
44	11	0	1	2	1	0.333333333	11	0	-11	1	5.65E-06	-1.13287417	0
45	18	4	0.768526326	2.231216765	0.818181818	0.256197382	23.42144879	1.79274379	-21.628705	0.999999996	5.45E-08	-2.227509203	0.222222222
46	10.00018441	1.000010848	1	1.995309392	0.909091537	0.333855328	10.00018441	0.501180845	-9.499003563	0.999994255	0.000131813	-0.978288707	0.099999241
47	20	22	0.999826168	2.000173832	0.476190476	0.333275389	20.00347725	10.99904401	-9.004433241	0.981364706	0.038299418	-0.927353621	1.1
48	41.16178363	17.00779321	0.768493637	2.231506363	0.707617037	0.256164546	53.56164532	7.621664673	-45.93998065	1	8.48E-13	-4.731292495	0.413193786
49	19	17.969036	1.064236427	1.932697209	0.513943615	0.35510844	17.85317578	9.297388084	-8.555787698	0.984155944	0.034521334	-0.881148263	0.945738737
50	30.00189908	2.000024084	1.706604278	1.292907883	0.937503003	0.568960613	17.57988039	1.546919244	-16.03296114	0.999999632	4.51E-06	-1.651211595	0.06666325
51	6	3	0.803670286	2.122669095	0.666666667	0.274633315	7.465748216	1.413314966	-6.052433249	0.997547869	0.016207474	-0.62333139	0.5
52	4.000196117	55.00276033	0.768940888	2.230831262	0.067796537	0.256333098	5.202215382	24.6557242	19.45350881	0.000205739	0.99995988	2.003488877	13.75001593
53	36.0291856	31	0.84459561	2.051186707	0.537514894	0.291664054	42.65850446	15.11320246	-27.545302	0.999992587	2.21E-05	-2.836851013	0.860413564
54	20	9	0.768496794	2.231369925	0.689655172	0.256176979	26.02483206	4.033396659	-21.9914354	0.999999826	1.21E-06	-2.264866284	0.45
55	27.00415705	20.00775042	0.768493637	2.231506363	0.574411006	0.256164546	35.13907694	8.966028847	-26.1730481	0.999999109	3.71E-06	-2.695524558	0.740913719
56	30.98904183	32	0.999477615	1.993275868	0.491975127	0.333965902	31.0052385	16.05397452	-14.95126397	0.996747332	0.006813754	-1.53980916	1.032623086
57	9	3	0.803266889	2.080396875	0.75	0.27855775	11.20424621	1.442032545	-9.762213667	0.999896371	0.000937818	-1.005396337	0.333333333
58	26.02438072	1	1.74858561	1.246159987	0.962996377	0.583884525	14.88310356	0.802465181	-14.08063838	0.999999484	1.02E-05	-1.450144683	0.038425506
59	9.002839431	0	0.768493637	2.086500362	1	0.26917522	11.71491733	0	-11.71491733	1	7.45E-06	-1.206502477	0
60	11.00115247	25	0.810057828	2.074256513	0.305577786	0.280849357	13.58070016	12.05251127	-1.528188891	0.703591672	0.432039507	-0.157385975	2.272489184
61	28.02958644	1.000022081	1.759457373	1.230135579	0.965551651	0.588527402	15.93081303	0.812936475	-15.11787655	0.999999776	4.63E-06	-1.55696835	0.035677376
62	8.000721296	44.01001505	0.875954121	2.010732245	0.153828264	0.303446239	9.133721851	21.88755622	12.75383437	0.010665575	0.995981421	1.313499048	5.500755922
63	29.00709158	6.000545744	0.999892648	1.993174154	0.828593238	0.334069606	29.0102059	3.010547639	-25.99965826	1	2.45E-09	-2.677667388	0.206864784
64	13.5	45.5	0.922897054	2.074673401	0.228813559	0.307881689	14.62785036	21.93116274	7.303312389	0.120835249	0.928669824	0.752157633	3.37037037
65	6	36.0013994	0.999889043	2.000110957	0.142852383	0.333296348	6.000665814	17.99970111	11.99903529	0.004716313	0.998581469	1.235763377	6.000233233
66	9.00036863	2.000028922	0.99998805	1.999958276	0.81818576	0.333335314	9.000476189	1.000035324	-8.000440865	0.999870117	0.001371987	-0.823953891	0.222216334
67	25.00913719	0	1.626269706	1.231988232	1	0.568972339	15.37822238	0	-15.37822238	1	7.68E-07	-1.583780993	0
68	5	6	0.771675883	2.15921841	0.454545455	0.263290247	6.479404255	2.778783273	-3.700620982	0.956356681	0.137096556	-0.381121629	1.2
69	24	0	1.625228243	1.233614123	1	0.56849173	14.76715662	0	-14.76715662	1	1.30E-06	-1.520848209	0
70	14	4	0.999894483	1.99984534	0.777777778	0.333327069	14.0014774	2.000154672	-12.00132272	0.99998148	0.000144866	-1.235998956	0.285714286
71	5	15	0.802260829	2.084391949	0.25	0.277920793	6.232387047	7.196343283	0.963956237	0.503567709	0.690888381	0.099276466	3
72	24	1	0.76853589	2.086484574	0.96	0.269187524	31.22820979	0.479275051	-30.74893474	1	3.91E-13	-3.166788539	0.041666667
73	8	18	0.768530926	2.231462	0.307692308	0.256177579	10.4094705	8.066460464	-2.343010033	0.799057873	0.341719609	-0.241303232	2.25
74	12.99235627	7	0.907866705	2.092133295	0.649866184	0.302622235	14.31086326	3.345867119	-10.96499614	0.999704376	0.001423574	-1.129269172	0.538778329
75	16.00779379	1.000021855	1	2	0.941202217	0.333333333	16.00779379	0.500010928	-15.50778286	0.999999992	2.73E-07	-1.59712424	0.062470936
76	25.01529284	2	0.999978919	1.991973193	0.925967858	0.334222903	25.01582019	1.004029576	-24.01179062	1	2.07E-10	-2.472939761	0.079951093
77	0	2	0.000188739	1.294706036	0	0.000145756	0	1.544752202	1.544752202	0.999708509	0.000291491	0.159091806	0
78	21.00243362	9	0.919665731	2.080334269	0.700024334	0.306555244	22.83702971	4.326227825	-18.51080188	0.999998142	1.06E-05	-1.906400847	0.428521769
79	35	3	1.738067591	1.24795104	0.921052632	0.582068569	20.13730662	2.403940463	-17.73336616	0.999999542	4.12E-06	-1.826333861	0.085714286
80	22.00511842	1	0.99998205	1.928078499	0.956531413	0.341516862	22.00551342	0.518651082	-21.48686234	1	8.47E-10	-2.212901031	0.045443973

81	24.00721873	3	0.768493637	2.231506363	0.888918588	0.256164546	31.2393201	1.34438335	-29.89493675	1	8.05E-12	-3.078836514	0.124962414
82	12	3	1.000399775	1.99959319	0.8	0.333467374	11.99520462	1.50030517	-10.49489945	0.999968416	0.000286335	-1.080854591	0.25
83	25.01761809	13.00024073	0.847576632	2.078672981	0.658049108	0.289646047	29.51664445	6.254105793	-23.26253866	0.999999446	2.79E-06	-2.39577538	0.519643424
84	7	1	0.768423602	2.086428197	0.875	0.269164095	9.109558819	0.479288001	-8.630270817	0.999972449	0.000626004	-0.888819172	0.142857143
85	12	13	0.801034567	2.130387071	0.48	0.273258052	14.98062693	6.102177476	-8.878449449	0.992317606	0.021900672	-0.914378732	1.083333333
86	10	26	1.01735513	1.759345572	0.277777778	0.366389914	9.829409322	14.77822232	4.948813	0.176658971	0.901317274	0.509671129	2.6
87	13	15.00274831	1.08145828	1.918324439	0.464240147	0.360512204	12.02080583	7.820756489	-4.200049339	0.908162738	0.171452751	-0.432557037	1.154057562
88	15.00115443	15	0.993975736	2.005960898	0.50001924	0.331332244	15.09207307	7.477713058	-7.614360009	0.982294979	0.041290303	-0.78419198	0.999923044
89	14	15.00375048	0.999591793	1.999569098	0.482696195	0.333290487	14.00571724	7.503491875	-6.502225364	0.96885618	0.068176837	-0.669654833	1.071696463
90	14	39.00255325	0.996523929	2.00033098	0.264138219	0.332523248	14.04883475	19.49804989	5.449215138	0.181918986	0.887158538	0.561206825	2.785896661
91	22.7418862	46.0022779	0.773810399	2.220524711	0.330819154	0.258424782	29.38948122	20.71685025	-8.672630976	0.929957596	0.112058981	-0.893181784	2.022799582
92	10	3	1.000052066	1.770328687	0.769230769	0.360980008	9.999479371	1.694600569	-8.304878803	0.99952561	0.00327815	-0.855307516	0.3
93	0	0	0	1.294639638	0	0	0	0	0	0	0	0	0
94	10.00054213	3	1	1.999404479	0.769240393	0.333399516	10.00054213	1.500446774	-8.500095361	0.999786897	0.001650679	-0.875412589	0.299983737
95	7	1.000065051	0.768493637	2.000021107	0.874992885	0.277583364	9.108728635	0.500027248	-8.608701386	0.999964745	0.000769212	-0.886597767	0.142866436
96	9	1.000065465	0.895866437	2.104133563	0.899994108	0.298622146	10.04614039	0.475286114	-9.570854281	0.99999436	0.000138103	-0.985688509	0.111118385
97	15	7.000021778	0.983938172	2.016061828	0.681817507	0.327979391	15.24486033	3.472126539	-11.77273379	0.999852365	0.000724693	-1.212456911	0.466668119
98	9	1	0.804296744	2.122043346	0.9	0.274847324	11.18989983	0.471243908	-10.71865592	0.99999754	6.74E-05	-1.103898948	0.111111111
99	8	11.00002171	0.999853641	2.000132296	0.421052151	0.333286109	8.001171046	5.499647062	-2.501523984	0.853947872	0.279186986	-0.257628355	1.375002714
100	18	46.00897039	0.999612173	1.989417275	0.281210585	0.334427007	18.00698359	23.12685779	5.119874197	0.222568507	0.849978708	0.527288476	2.556053911
101	9	61.10478897	0.773007277	2.226802298	0.128379247	0.257685449	11.64283994	27.44059902	15.79775909	0.006678715	0.997408827	1.626988474	6.789420997
102	6	9	1.000034735	1.780999236	0.4	0.359590981	5.999791599	5.053342989	-0.94644861	0.728967495	0.467085635	-0.09747338	1.5
103	6	45.00002201	0.776841097	2.215573034	0.117647008	0.259603472	7.723587262	20.3107825	12.58719524	0.011174519	0.99619194	1.296337124	7.500003668
104	5	7	0.768624712	2.230952522	0.416666667	0.256244348	6.505125222	3.137673227	-3.367451995	0.939353336	0.170802778	-0.346809035	1.4
105	26.00469586	3	0.996625261	1.930364504	0.896568472	0.340494959	26.09275208	1.554110632	-24.53864144	1	7.62E-10	-2.527199369	0.11536378
106	30.03381181	13.00002196	0.992037867	2.007940906	0.697911601	0.330681629	30.27486429	6.474305058	-23.80055924	0.999999808	9.47E-07	-2.451185344	0.432846222
107	11	0	0.768493637	2.086500362	1	0.26917522	14.31371643	0	-14.31371643	1	5.38E-07	-1.474149056	0
108	18	2	0.768493637	2.000058151	0.9	0.27757965	23.42244506	0.999970925	-22.42247414	1	9.88E-09	-2.309258343	0.111111111
109	10	0	1	2	1	0.333333333	10	0	-10	1	1.69E-05	-1.029885609	0
110	20.00379396	2	0.999978765	1.927288238	0.909106584	0.341608321	20.00421875	1.037727497	-18.96649125	0.999999998	4.94E-08	-1.95333164	0.099981034
111	12	21.01275788	0.769618048	2.226571456	0.363495835	0.256865611	15.59214994	9.437270842	-6.154879096	0.94100982	0.116494754	-0.633882141	1.751063157
112	5	2	0.768560284	2.231432689	0.714285714	0.256187361	6.505670546	0.896285158	-5.609385388	0.998455572	0.014365811	-0.577702529	0.4
113	21	17.00284245	0.916971772	2.083028228	0.552590245	0.305657257	22.90146834	8.162559785	-14.73890856	0.999564038	0.001340269	-1.517938982	0.809659164
114	5	40.9869355	0.768500662	2.231499338	0.108726532	0.256166887	6.506175269	18.36744237	11.8612671	0.011899633	0.996249359	1.221574829	8.1973871
115	2	6	0.768507692	2.000127021	0.25	0.277576413	2.602446302	2.999809481	0.397363179	0.60890325	0.697769123	0.040923862	3
116	31.01480695	4.000110013	0.851255609	2.075087308	0.885759832	0.290894004	36.43418807	1.927682752	-34.50650532	1	3.35E-13	-3.553775325	0.128974203
117	26	1	0.850467272	2.075879534	0.962962963	0.290624225	30.5714292	0.481723522	-30.08970568	1	2.19E-13	-3.098895486	0.038461538
118	27.00946633	3	1.762876587	1.232368635	0.900031544	0.58855835	15.32124626	2.43433654	-12.88690972	0.999970691	0.000202094	-1.327204287	0.111072169
119	23.5	9.5	0.888885267	2.007243576	0.712121212	0.306921866	26.43760771	4.732858589	-21.70474912	0.999999525	2.71E-06	-2.235340877	0.404255319
120	13.02409939	6.000434912	0.852138584	2.074164835	0.684594912	0.291199668	15.28401558	2.892940238	-12.39107534	0.99992428	0.000447615	-1.276139017	0.460717838
121	13	4	0.806379026	1.42701057	0.764705882	0.36105614	16.12145105	2.803062629	-13.31838842	0.999872752	0.000830157	-1.371641657	0.307692308
122	25.00340623	3	1.623640662	1.233837656	0.892870175	0.568207518	15.39959353	2.431438192	-12.96815534	0.999967752	0.000224753	-1.335571656	0.119983652

123	19	10	0.768675598	2.231270932	0.655172414	0.256229766	24.71783941	4.481750672	-20.23608874	0.999998787	7.24E-06	-2.084085658	0.526315789
124	10	2	1.000069519	1.773701064	0.833333333	0.360545146	9.999304863	1.127585725	-8.871719138	0.99989248	0.001109272	-0.913685587	0.2
125	29.00707629	14	1.000055342	1.999875534	0.674472175	0.333359462	29.00547107	7.000435658	-22.00503542	0.999998851	5.07E-06	-2.26626693	0.482640851
126	11	2	1	1.773821636	0.846153846	0.360513447	11	1.12750908	-9.87249092	0.999958191	0.000468284	-1.016753632	0.181818182
127	11.75	8.25	1.000459989	1.768884478	0.5875	0.361262386	11.74459762	4.663956352	-7.080641266	0.987525722	0.035574512	-0.729225054	0.70212766
128	7.75	8.25032647	0.771953482	2.227162028	0.484365117	0.257393715	10.03946504	3.704412328	-6.335052717	0.985109342	0.04679459	-0.652437963	1.064558254
129	6	24.00446429	0.804728731	2.121059954	0.199970243	0.275046771	7.455928645	11.31720215	3.861273509	0.241952355	0.872151927	0.397667002	4.000744048
130	44.04908589	20.0100833	0.924596303	2.056307336	0.687631239	0.31017316	47.64142552	9.731076159	-37.91034936	1	6.25E-10	-3.904332324	0.454267845
131	4	13	0.768988924	2.2309689	0.235294118	0.256333245	5.201635389	5.827064644	0.625429255	0.549654024	0.668978854	0.064412059	3.25
132	32	2	0.844394644	2.081908518	0.941176471	0.288553372	37.89697179	0.960657004	-36.93631479	1	-1.11E-15	-3.804017905	0.0625
133	4	32	0.210701621	2.789298379	0.111111111	0.070233874	18.98419191	11.4724191	-7.511772813	0.894765529	0.244645675	-0.773626672	8
134	19	3	0.768603788	2.231302688	0.863636364	0.25620925	24.72014879	1.344506066	-23.37564272	1	3.87E-09	-2.407423804	0.157894737
135	13	6	1	2	0.684210526	0.333333333	13	3	-10	0.999619202	0.001874823	-1.029885609	0.461538462
136	39.02709039	23.03762582	0.849726945	2.034142062	0.628812839	0.294648246	45.92897826	11.32547537	-34.60350289	0.999999988	5.22E-08	-3.563764965	0.590298318
137	8	32	0.989469181	2.006849868	0.2	0.330228245	8.085143179	15.9453881	7.860244921	0.052465335	0.97678461	0.809515313	4
138	12	48.00742358	0.774438486	2.225263998	0.199975258	0.258171765	15.49509769	21.5738104	6.078712711	0.190145418	0.882824715	0.626037874	4.000618632
139	30.0163675	1	1.00005209	1.769960181	0.967758958	0.361028036	30.01480402	0.564984461	-29.44981956	1	1.10E-12	-3.032994535	0.033315157
140	0	0	0	1.294639638	0	0	0	0	0	0	0	0	0
141	16.00158661	6.000174128	1	2	0.727286639	0.333333333	16.00158661	3.000087064	-13.00149955	0.99996898	0.000183214	-1.339005728	0.374973699
142	12	44.03583263	0.769438534	2.228024958	0.214148687	0.256696549	15.59578767	19.76451497	4.168727299	0.288183958	0.809625504	0.429331225	3.669652719
143	8.003924204	6	0.769044165	2.230728898	0.571548666	0.256367448	10.40762621	2.689703803	-7.717922408	0.997396741	0.012069017	-0.794857722	0.749632286
144	29.00449346	41.17256522	0.770209489	2.228787676	0.41330449	0.256822347	37.65792796	18.47307649	-19.18485147	0.998622245	0.0030313	-1.975820244	1.419523677
145	36	47.72743442	0.99569126	1.879187706	0.429966596	0.346341975	36.15578587	25.3979069	-10.75787897	0.955836717	0.069248744	-1.107938474	1.325762067
146	8	31.00432592	0.781149327	2.218829469	0.205105455	0.260384949	10.24131971	13.97328021	3.731960504	0.279506593	0.833474613	0.384349242	3.87554074
147	5	15	0.805520309	2.065567815	0.25	0.280562725	6.207168141	7.261925699	1.054757559	0.492916425	0.700052619	0.108627963	3
148	19.00650481	2	1	1.997597727	0.904791396	0.333600467	19.00650481	1.001202581	-18.00530223	0.999999996	8.61E-08	-1.854340165	0.105227132
149	18	19.00219852	0.993596229	2.006318776	0.486457581	0.331208127	18.11601079	9.47117614	-8.64483465	0.983416029	0.036077384	-0.89031908	1.055677696
150	41	7	0.999957993	1.999747983	0.854166667	0.333352002	41.00172235	3.500441085	-37.50128126	1	1.32E-13	-3.86220299	0.170731707
151	13	13.99966835	0.99992673	2.00007327	0.481487396	0.33330891	13.00095258	6.999577743	-6.001374842	0.964099192	0.078974834	-0.618072958	1.076897565
152	7	4	1	1.997210661	0.636363636	0.333643548	7	2.002793235	-4.997206765	0.9911212	0.038823467	-0.514655133	0.571428571
153	33	0	0.768493637	2.086500362	1	0.26917522	42.94114928	0	-42.94114928	1	-1.78E-15	-4.422447168	0
154	16.00287136	29.99568824	0.999971854	2.000028146	0.347899402	0.333323951	16.00332179	14.99763306	-1.005688728	0.648248506	0.471823826	-0.103574435	1.874394136
155	18.5	31.50560826	0.774419622	2.215955514	0.369958504	0.258970727	23.88885749	14.21761767	-9.671239825	0.969627469	0.057297726	-0.996027072	1.703005852
156	7	30	0.996971805	2.001968498	0.189189189	0.332441364	7.021261749	14.98525078	7.963989034	0.042404015	0.98267637	0.82019977	4.285714286
157	23	11	0.999553838	1.931530321	0.676470588	0.341018471	23.01026631	5.694966257	-17.31530005	0.999984504	6.77E-05	-1.783277834	0.47826087
158	4	37.00189973	0.989864846	2.004211487	0.097556455	0.330607752	4.04095571	18.46207347	14.42111776	0.000536443	0.999892768	1.485210165	9.250474933
159	7	19	0.799404965	2.097606307	0.269230769	0.275941268	8.756513043	9.057943781	0.301430738	0.569452332	0.604132819	0.031043918	2.714285714
160	15	32.11300863	0.786802773	2.213197227	0.318383403	0.262267591	19.06449813	14.5097817	-4.554716428	0.850919936	0.23490188	-0.46908369	2.140867242
161	15	1	0.768519519	2.23147608	0.9375	0.256173549	19.51804688	0.448133865	-19.06991302	1	1.63E-08	-1.963982898	0.066666667
162	4	1.000021717	0.768493637	2.000021112	0.799996525	0.277583364	5.204987791	0.500005581	-4.704982211	0.99835189	0.023093825	-0.484559347	0.250005429
163	6	14	0.817413329	2.065993695	0.3	0.283488707	7.340227753	6.776400157	-0.563827596	0.6702867	0.51882609	-0.058067793	2.333333333
164	14.09820886	8	0.768877414	2.231122586	0.637979709	0.256292471	18.33609443	3.585638929	-14.7504555	0.999966245	0.000188955	-1.519128185	0.56744797

165	16	3.000192123	1.577153061	1.201344777	0.842096748	0.56762796	10.1448619	2.49736144	-7.647500464	0.99756308	0.011535846	-0.787605067	0.187512008
166	6	19.00041565	0.33604369	2.663900067	0.23999601	0.112016663	17.85482118	7.132555714	-10.72226546	0.982851772	0.053761019	-1.10427069	3.166735942
167	0	1	2.33E-05	1.294663682	0	1.80E-05	0	0.772401368	0.772401368	0.999981976	1.80E-05	0.079548505	0
168	29.5	6.5	1.733943647	1.265019598	0.819444444	0.578181026	17.01324034	5.138260316	-11.87498003	0.999335113	0.00231224	-1.222987104	0.220338983
169	14	17.00733112	0.998804301	2.001195699	0.451506128	0.332934767	14.01675983	8.498584686	-5.51817514	0.941533896	0.114443925	-0.568308916	1.214809366
170	12	12.00080602	0.807566003	2.077336376	0.499983208	0.279928364	14.85946655	5.777016259	-9.082450289	0.993861741	0.018306382	-0.935388485	1.000067168
171	20	21.00396894	0.806005968	2.077898282	0.487757661	0.279484303	24.81371204	10.10827581	-14.70543623	0.998632445	0.003702032	-1.514491715	1.050198447
172	58.54097734	62.5725955	0.992907078	1.937543363	0.483356043	0.338824047	58.95917014	32.29481037	-26.66435977	0.99961136	0.00074191	-2.746124041	1.06886831
173	14.92845198	50.25464844	0.779080261	2.220774938	0.229023349	0.259705956	19.1616355	22.62932978	3.467694281	0.343407095	0.755627596	0.357132844	3.366367022
174	17	9.00023938	1.002146193	1.773308828	0.653840134	0.361074557	16.96359286	5.075393094	-11.88819977	0.999389045	0.002282223	-1.224348586	0.529425846
175	27	2	0.768582643	2.000056654	0.931034483	0.277603025	35.12959896	0.999971674	-34.12962728	1	2.02E-13	-3.514961198	0.074074074
176	10.00026094	6	1.000028719	1.999781279	0.625006116	0.33336402	9.999973746	3.000328117	-6.999645629	0.995957238	0.015955756	-0.72088343	0.599984344
177	39.00811699	23.00111834	0.850175496	2.036314768	0.629069473	0.294536069	45.88242918	11.29546311	-34.58696606	0.999999988	5.05E-08	-3.562061861	0.589649543
178	15.00032733	10	1.0374915	1.9625085	0.600005237	0.3458305	14.45826527	5.095519332	-9.362745937	0.997447005	0.008231132	-0.96425573	0.666652119
179	11.98841952	46	0.770610186	2.228355601	0.206738166	0.256958645	15.55704783	20.64302483	5.085976998	0.238129241	0.847820159	0.523797452	3.837036227
180	12	60.00713807	0.799788261	1.972926021	0.166650145	0.288449577	15.00397116	30.41530064	15.41132948	0.012575395	0.994329701	1.587190645	5.000594839
181	31	10	0.999915615	1.994435958	0.756097561	0.333933939	31.00261615	5.01394891	-25.98866724	0.999999994	3.89E-08	-2.676535439	0.322580645
182	16	4	0.768722558	2.00005282	0.8	0.277639914	20.8137511	1.999947182	-18.81380392	0.999999841	1.80E-06	-1.937606591	0.25
183	14.83333333	18.16666667	0.9176065	2.0823935	0.449494949	0.305868833	16.1652444	8.723935542	-7.441308855	0.971910429	0.059613685	-0.76636969	1.224719102
184	8	4	0.76866437	2.23133563	0.666666667	0.256221457	10.40766337	1.792648289	-8.615015077	0.999521791	0.003292077	-0.887248005	0.5
185	31	17.00238418	0.76914094	2.229775873	0.645801256	0.256472916	40.30470672	7.625153894	-32.67955283	0.999999997	1.61E-08	-3.365620117	0.548464006
186	15.00555122	8	0.80366572	2.080028383	0.652257843	0.278693125	18.67138395	3.846101363	-14.82528259	0.99996102	0.000209605	-1.526834519	0.53313603
187	29.99335541	35.00586036	0.995861028	2.000065092	0.461441804	0.332405069	30.11801302	17.50236054	-12.61565248	0.989004781	0.020729222	-1.299267893	1.167120513
188	8	13	0.806334471	2.077367379	0.380952381	0.279617836	9.921441153	6.257920545	-3.663520608	0.896516722	0.210482447	-0.377300715	1.625
189	17	17.00218322	0.803448199	2.122836329	0.499967896	0.274562569	21.15880032	8.009182329	-13.14961799	0.998532332	0.004321764	-1.354260233	1.000128425
190	24.34170281	28.66150619	0.999285054	2.00070638	0.459249605	0.333095969	24.35911826	14.3256934	-10.03342486	0.979160091	0.03920826	-1.033327987	1.177465127
191	48.99408684	0	1.004798381	1.995201619	1	0.334932794	48.7601172	0	-48.7601172	1	-2.00E-15	-5.021734301	0
192	31	2	0.999969665	2.000030335	0.939393939	0.333323222	31.00094041	0.999984833	-30.00095558	1	3.92E-13	-3.089755241	0.064516129
193	34.28679155	9	1.740844099	1.258871271	0.792084382	0.580336427	19.69549805	7.14926157	-12.54623648	0.99894152	0.003125191	-1.29211884	0.262491752
194	0	1	0.000142024	1.294757145	0	0.00010968	0	0.772345612	0.772345612	0.99989032	0.00010968	0.079542763	0
195	17	2	1	1.997880445	0.894736842	0.333569006	17	1.001060902	-15.9989391	0.999999966	6.29E-07	-1.647707714	0.117647059
196	13	14	0.917467769	2.082532231	0.481481481	0.30582259	14.16943509	6.72258503	-7.446850057	0.98302927	0.041744101	-0.766940371	1.076923077
197	22	1	0.769027745	2.230972255	0.956521739	0.256342582	28.60755042	0.448235068	-28.15931535	1	1.71E-12	-2.900087365	0.045454545
198	23.07699978	3	0.769048843	2.230951157	0.884956091	0.256349614	30.00719655	1.34471792	-28.66247863	1	2.99E-11	-2.951907426	0.129999568
199	15	12	0.999557981	1.998702402	0.555555556	0.333379311	15.00663321	6.00389532	-9.002737894	0.994950845	0.014398289	-0.92717902	0.8
200	18	53.27713325	0.999293897	2.000492043	0.252535409	0.333121735	18.01271883	26.63201458	8.619295748	0.091712105	0.944577598	0.887688865	2.959840736
201	19.50437713	17.50712602	0.988179488	2.011790603	0.526981491	0.329396447	19.73768668	8.702260561	-11.03542612	0.99554811	0.011035266	-1.136522655	0.897599852
202	9.5	68.25676681	0.848566659	2.151356554	0.122175862	0.282862793	11.19534912	31.72731488	20.53196575	0.000679176	0.99977008	2.114557605	7.184922822
203	16.50226398	66.51875691	0.984429132	2.011313027	0.198772116	0.328609433	16.76328284	33.07230451	16.30902168	0.006807895	0.996760422	1.679642672	4.030886731
204	24	23.00002182	0.769111851	2.230323796	0.510638061	0.256418854	31.20482405	10.31241377	-20.89241028	0.999948862	0.000166702	-2.151679269	0.958334243
205	5	7	0.80325005	1.429321208	0.416666667	0.359786971	6.22471172	4.897429605	-1.327282114	0.76525076	0.445235572	-0.136694875	1.4
206	8	3	0.806276317	1.437932457	0.727272727	0.359269746	9.922156753	2.086328871	-7.835827882	0.997482981	0.014563905	-0.807000637	0.375

207	19.5	39.5067769	0.779503778	2.220474949	0.330470516	0.259836435	25.01591467	17.79203901	-7.223875665	0.912179465	0.141267896	-0.743976559	2.025988559
208	14.0001527	40.110708	0.99892513	2.00107487	0.258730919	0.332975043	14.01521723	20.04458134	6.029364113	0.15494772	0.906078305	0.620955533	2.865019322
209	9	2.5	0.76850305	2.000044639	0.782608696	0.277583461	11.71107909	1.249972101	-10.46110699	0.999950173	0.000542315	-1.077374355	0.277777778
210	30.33489496	5.673965937	0.854980488	1.394283449	0.842428619	0.380115679	35.48021902	4.069449393	-31.41076963	0.999999997	2.22E-08	-3.234949961	0.187044193
211	4	11.0016726	0.770190595	2.227794449	0.266636935	0.256902748	5.19351966	4.938369699	-0.255149961	0.66339323	0.563442194	-0.026277527	2.75041815
212	6.000283194	18.00041384	0.982543224	2.012357508	0.250004539	0.328072051	6.106889798	8.944938349	2.83804855	0.281290658	0.849522777	0.292286536	2.99992738
213	7	12.00724557	0.768508373	2.231460301	0.36828061	0.256172133	9.108553978	5.380891412	-3.727662566	0.91234403	0.192413891	-0.383906603	1.715320796
214	10.00040522	45.83129022	1	1.999508342	0.17911699	0.333387971	10.00040522	22.92127983	12.92087461	0.008217093	0.996734818	1.330702282	4.582943312
215	3.000151752	4	0.768493637	2.000569247	0.428583816	0.277528416	3.90393831	1.999430915	-1.904507395	0.901220084	0.302599102	-0.196142476	1.333265891
216	16	18.0074373	0.999366012	2.000633988	0.470485319	0.333122004	16.01015024	9.000865428	-7.009284814	0.9674695	0.067076342	-0.721876156	1.125464831
217	7	5	0.791377687	2.203644725	0.583333333	0.264230973	8.845334044	2.268968288	-6.576365756	0.995939567	0.01941787	-0.677290445	0.714285714
218	15	3.000065371	0.999897248	2.000098343	0.833330307	0.333299572	15.00154144	1.49995893	-13.50158251	0.999998327	1.85E-05	-1.390508553	0.200004358
219	33	27.02771892	0.999338598	1.997626817	0.549746027	0.33345016	33.02184072	13.52991394	-19.49192678	0.999823984	0.000459555	-2.007445488	0.819021785
220	5	55.11548505	1.000013779	1.971322805	0.083173246	0.336553517	4.999931108	27.95863007	22.95869896	4.32E-06	0.999999272	2.364483367	11.02309701
221	5	0	1	1.768574932	1	0.361196653	5	0	-5	1	0.006147784	-0.514942805	0
222	6	64.10967967	0.870812894	2.01873502	0.085580194	0.301366484	6.890113869	31.75735251	24.86723864	1.29E-05	0.999997364	2.561041122	10.68494661
223	11	7	0.769027745	2.000129704	0.611111111	0.277711816	14.30377521	3.499773033	-10.80400218	0.999331844	0.003141396	-1.112688636	0.636363636
224	9.000500544	13.0149244	0.768774676	2.226316274	0.408827019	0.256678241	11.70759239	5.84594586	-5.861646529	0.964746335	0.086284942	-0.603682541	1.446022289
225	10	12.00002196	0.858844556	2.033562426	0.454545001	0.296930743	11.64355055	5.900985287	-5.742565267	0.963961322	0.08629582	-0.591418533	1.200002196
226	34.00625368	31.00650751	0.856485452	2.031868577	0.523070441	0.296530634	39.7044148	15.260095	-24.4443198	0.999960188	0.00011004	-2.517485319	0.911788396
227	7	12	0.768987895	2.230948705	0.368421053	0.256334716	9.102874109	5.378877594	-3.723996515	0.912249077	0.192586625	-0.383529042	1.714285714
228	35	13.00041645	0.865556354	2.028827248	0.72916034	0.299046869	40.43641971	6.407847914	-34.02857179	1	1.00E-09	-3.504553639	0.37144047
229	37.00745263	1	1.0000044	1.9999956	0.973689371	0.3333348	37.0072898	0.5000011	-36.5072887	1	2.22E-16	-3.759833126	0.027021584
230	12	2	0.803296	2.122881159	0.857142857	0.274520631	14.93845358	0.942115856	-13.99633772	0.999999475	9.30E-06	-1.44146268	0.166666667
231	3	5	0.197706732	2.802293268	0.375	0.065902244	15.17399015	1.784252939	-13.38973721	0.998935985	0.012462583	-1.378989766	1.666666667
232	12	53.0328568	0.999336286	2.000324339	0.184522111	0.333149783	12.00796985	26.51212895	14.50415909	0.006041544	0.997484991	1.493762472	4.419404733
233	4.004818747	36.69318308	0.349910593	2.646307455	0.098403326	0.116784088	11.44526295	13.86580498	2.420542028	0.477163481	0.714426469	0.24928814	9.162258119
234	16	1.000173717	0.883704942	2.000452681	0.941166853	0.306399669	18.10559073	0.499973694	-17.60561703	0.999999998	7.30E-08	-1.813177162	0.062510857
235	14.00279742	6	0.999985938	2.000014062	0.700041955	0.333328646	14.00299432	2.999978908	-11.00301541	0.999832621	0.000878593	-1.133184723	0.42848581
236	7	7	0.804287647	2.079437414	0.5	0.278905801	8.703353869	3.366295111	-5.337058758	0.979665035	0.066011602	-0.549656001	1
237	24	9.000065628	0.997518388	2.002468319	0.727271281	0.332507603	24.05970685	4.494485901	-19.56522094	0.999999287	4.10E-06	-2.014993949	0.375002735
238	20.00466671	16.00058845	0.927814071	2.060062919	0.555604081	0.310526194	21.56107277	7.767038719	-13.79403405	0.999356272	0.001967064	-1.420627716	0.799842791
239	10	218.6119347	0.779614392	2.216997504	0.043742248	0.260165286	12.82685403	98.60720832	85.7803543	3.53E-18	1	8.834395243	21.86119347
240	11	80.21537526	0.807031983	1.426148447	0.120593704	0.361382346	13.6301909	56.24616105	42.61597015	2.07E-07	0.999999952	4.388957437	7.292306842
241	13	16.00178475	0.824473179	2.109722588	0.448248275	0.280987788	15.76764451	7.584781453	-8.182863057	0.983381684	0.040198682	-0.84274129	1.230906519
242	24.99843679	6.000065181	0.999904339	1.999114955	0.806440157	0.333410439	25.0008284	3.00136076	-21.99946764	0.99999999	8.71E-08	-2.265693513	0.240017615
243	16	0	0.887048938	2.004523832	1	0.306770401	18.03733628	0	-18.03733628	1	6.15E-09	-1.857639306	0
244	1	10	0.009763459	2.990236541	0.090909091	0.003254486	102.4227185	3.344217042	-99.07850144	0.999428722	0.035222458	-10.20395228	10
245	13.00338571	15	0.76852305	2.231452225	0.464350484	0.256176461	16.91996838	6.722079833	-10.19788854	0.995202158	0.013833318	-1.050265865	1.153545725
246	23	2	0.768493637	2.000569307	0.92	0.27752841	29.9286798	0.999715428	-28.92896437	1	2.54E-11	-2.979352409	0.086956522
247	12	18	0.769027745	2.000998407	0.4	0.277624724	15.60411841	8.995509412	-6.608609001	0.95136847	0.100656458	-0.680611131	1.5
248	0	1	0.0002239	1.294749212	0	0.000172899	0	0.772350344	0.772350344	0.999827101	0.000172899	0.07954325	0

249	8.001586336	10	0.999787455	2.000187819	0.444493401	0.333265232	8.003287396	4.999530498	-3.003756898	0.892561471	0.222973362	-0.3093526	1.249752184
250	35	9	1.759770344	1.233098327	0.795454545	0.587987826	19.88895888	7.29868803	-12.59027085	0.998965666	0.003088737	-1.296653876	0.257142857
251	11	51.03942895	1.00827357	1.98670539	0.177306597	0.336654642	10.90973752	25.69048698	14.78074945	0.004219371	0.998351828	1.522248116	4.639948086
252	43.00738675	40.00225595	0.845800169	2.081237767	0.518101095	0.288961123	50.84816522	19.22041613	-31.62774909	0.999996489	9.74E-06	-3.257296364	0.930125241
253	46.5	4.5	0.996237226	1.991292089	0.911764706	0.333465255	46.67562983	2.25983924	-44.41579059	1	8.88E-16	-4.574318355	0.096774194
254	17	3	0.999947372	1.927630938	0.85	0.341561272	17.00089473	1.556314511	-15.44458021	0.999999652	4.16E-06	-1.59061509	0.176470588
255	4.5	12.5	0.768756335	2.231227945	0.264705882	0.256253454	5.853610302	5.602296274	-0.251314028	0.647900875	0.559373793	-0.02588247	2.777777778
256	7	3	0.824147078	2.071827255	0.7	0.284583695	8.493629584	1.447997169	-7.045632416	0.998918072	0.007724372	-0.725619543	0.428571429
257	3	1	0.197103042	2.802896958	0.75	0.065701014	15.22046523	0.356773729	-14.8636915	0.999981367	0.001078526	-1.530790198	0.333333333
258	10	12.00375695	0.959734177	2.040241066	0.454467845	0.319914032	10.41955183	5.883499333	-4.536052502	0.939766433	0.131370453	-0.467161519	1.200375695
259	32.04237839	2	0.76851721	2.231453952	0.941249698	0.256174866	41.69376815	0.896276617	-40.79749154	1	2.55E-15	-4.201674942	0.062417339
260	10.00379396	3	0.805792214	2.120460537	0.769298098	0.275366581	12.41485556	1.414786999	-11.00006856	0.999969834	0.00030309	-1.132881231	0.299886224
261	7	5.000086768	0.999545834	2.000284225	0.583329115	0.33320082	7.003180605	2.499688148	-4.503492458	0.981287818	0.066317835	-0.463808207	0.71429811
262	10	11	0.988922602	2.011077398	0.476190476	0.329640867	10.11201482	5.469704949	-4.642309868	0.948368953	0.11727859	-0.478104813	1.1
263	14	1	0.999982065	1.928087435	0.933333333	0.341515823	14.0002511	0.518648678	-13.48160242	0.9999999	3.00E-06	-1.388450832	0.071428571
264	9.000585252	1	0.768493637	2.231506363	0.900005852	0.256164546	11.71198409	0.448127783	-11.2638563	0.999998782	3.66E-05	-1.160048351	0.111103886
265	28.00067476	3	1.761362802	1.231637116	0.903227913	0.588494103	15.89716482	2.435782393	-13.46138243	0.999981793	0.000130092	-1.386368404	0.107140275
266	27.00042344	37.00028976	0.95177636	2.042346316	0.421876915	0.317881551	28.36845352	18.11656009	-10.25189343	0.970380797	0.051580209	-1.055827751	1.370359611
267	21	30.00536828	0.999186416	2.000806549	0.411721368	0.33306292	21.01709918	14.99663638	-6.0204628	0.908239766	0.148582137	-0.6200388	1.428827061
268	36	2	1.000093531	1.999888854	0.947368421	0.333366468	35.99663321	1.000055576	-34.99657763	1	5.55E-15	-3.604247167	0.055555556
269	19.00672207	10	0.828911048	1.967818346	0.655252325	0.296385861	22.92974874	5.081769881	-17.84797886	0.999985962	6.90E-05	-1.838137658	0.526129648
270	8.006309901	7	0.768493637	2.004852428	0.533529559	0.277099799	10.41818632	3.491528803	-6.926657518	0.991234163	0.03179922	-0.71336649	0.874310398
271	11	1	1	2	0.916666667	0.333333333	11	0.5	-10.5	0.999998118	4.70E-05	-1.08137989	0.090909091
272	22	1	0.768493637	2.231457652	0.956521739	0.256168705	28.62743285	0.448137566	-28.17929529	1	1.69E-12	-2.902145069	0.045454545
273	7	39	0.999806304	2.000087688	0.152173913	0.333280545	7.001356134	19.49914508	12.49778894	0.004930738	0.998395902	1.287129298	5.571428571
274	3	27	0.169232526	2.826744925	0.1	0.056486582	17.72708869	9.551622347	-8.175466338	0.913363806	0.238887177	-0.841979513	9
275	9	26	0.80002488	2.133034713	0.257142857	0.272761209	11.24965014	12.18920622	0.939556086	0.504610498	0.64483987	0.096763529	2.888888889
276	12	23.00529491	0.881977623	2.001996902	0.342805282	0.305820185	13.60578737	11.49117408	-2.114613292	0.748951386	0.377219644	-0.21778098	1.917107909
277	10	30	0.793770341	2.206077228	0.25	0.264603558	12.59810234	13.59879864	1.00069629	0.499375482	0.641821199	0.103060271	3
278	8	47.00359197	0.989652919	2.010347081	0.145445047	0.329884306	8.083642103	23.38083429	15.29719219	0.001687613	0.999463304	1.575435809	5.875448996
279	18.5	16.50435692	0.99531955	1.935577925	0.528505638	0.339595485	18.5869955	8.526836716	-10.06015879	0.992621066	0.017726713	-1.036081276	0.892127401
280	10	15.00045716	0.999788898	1.994983961	0.399992686	0.33384465	10.00211146	7.519086595	-2.483024867	0.820538476	0.306366372	-0.255723158	1.500045716
281	12	6.000283015	0.9998802	1.997287563	0.666656185	0.333608353	12.00143777	3.00421588	-8.997221887	0.999139578	0.003950507	-0.926610934	0.500023585
282	14	4	0.913155345	2.086844655	0.777777778	0.304385115	15.33145492	1.916769411	-13.41468551	0.999994677	4.73E-05	-1.381559155	0.285714286
283	12.5	24.4982399	0.891430571	2.108536082	0.33785391	0.297146827	14.02240444	11.61860122	-2.403803221	0.76466201	0.353467156	-0.247564234	1.959859192
284	11	18	0.901673521	2.098326479	0.379310345	0.30055784	12.19953758	8.578264717	-3.621272859	0.869182345	0.231229113	-0.37294968	1.636363636
285	8	9	1.000104625	1.772860891	0.470588235	0.360662482	7.999163085	5.076540435	-2.92262265	0.882932846	0.241279399	-0.300996701	1.125
286	16	23	0.768784211	2.231076101	0.41025641	0.256273336	20.81208196	10.3089267	-10.50315526	0.988855995	0.025539709	-1.081704845	1.4375
287	11	15.00015257	0.99969035	2.000241955	0.423074441	0.333237636	11.0034072	7.499169051	-3.504238148	0.879686013	0.219816266	-0.360896444	1.363650234
288	19	28	0.99671757	1.97580731	0.404255319	0.335310085	19.06257155	14.17142241	-4.891149138	0.875202757	0.197282939	-0.503732411	1.473684211
289	33.5	53.51771657	0.950509341	2.049490659	0.384979075	0.316836447	35.24426173	26.11269114	-9.131570591	0.927617738	0.108121687	-0.940447314	1.597543778
290	35	38.00002202	0.769279381	2.229226667	0.47945191	0.25655422	45.49712481	17.04628003	-28.45084478	0.999987815	3.44E-05	-2.93011156	1.085714915

291	10	37	0.771794008	2.224494488	0.212765957	0.257583343	12.95682514	16.63299244	3.676167302	0.303142628	0.805828279	0.37860318	3.7
292	9	4	0.768509385	2.086280586	0.692307692	0.269199974	11.71098256	1.91728765	-9.79369491	0.999753065	0.001761108	-1.008638545	0.444444444
293	24.5	13.5	0.772382406	2.22703533	0.644736842	0.257510782	31.72003895	6.061870604	-25.65816835	0.999999852	7.94E-07	-2.642497834	0.551020408
294	6	16	1	1.999879916	0.272727273	0.333346677	6	8.000480366	2.000480366	0.3619209	0.793887926	0.206026594	2.666666667
295	12	17	0.986301591	2.00306373	0.413793103	0.329936787	12.16666394	8.486999061	-3.679664882	0.875435746	0.220122994	-0.378963391	1.416666667
296	17	0	0.768493637	2.086500362	1	0.26917522	22.12119811	0	-22.12119811	1	2.04E-10	-2.278230359	0
297	37.01367755	1	0.846712746	1.38492079	0.973693679	0.379413883	43.71456283	0.722062956	-42.99249987	1	6.11E-15	-4.427735692	0.02701704
298	8	2	1	1.999850811	0.8	0.333349911	8	1.0000746	-6.9999254	0.999644212	0.003405166	-0.720912243	0.25
299	20	14.00089154	1	2	0.58821987	0.333333333	20	7.000445768	-12.99955423	0.999364047	0.00200402	-1.338805383	0.700044577
300	29.02137185	67	0.854178791	2.001293544	0.302238671	0.299137477	33.97575797	33.47834715	-0.497410824	0.576131376	0.511998494	-0.051227625	2.308643449
301	16	3	0.999964812	1.995032631	0.842105263	0.333878352	16.00056303	1.503734803	-14.49682823	0.999999361	7.47E-06	-1.493007477	0.1875
302	19	13.00006541	0.999957729	2.000042271	0.593748786	0.333319243	19.00080319	6.499895326	-12.50090786	0.999313571	0.002221366	-1.287450511	0.684213969
303	0.5	27.5	0.228337032	2.768328647	0.017857143	0.076197032	2.189745553	9.933791651	7.744046098	0.234216839	0.891301917	0.797548163	55
304	14	4.000021703	0.76972071	2.227953299	0.77777684	0.256772654	18.18841538	1.795379511	-16.39303586	0.999999503	5.55E-06	-1.688295173	0.285715836
305	11.00067553	5	0.979683871	2.020316129	0.687513193	0.32656129	11.22880131	2.47486021	-8.753941097	0.9993581	0.003366837	-0.901555796	0.454517542
306	21.01359438	7	0.769266637	2.230621849	0.750121319	0.256431744	27.31639898	3.138138365	-24.17826062	0.999999993	6.48E-08	-2.490084266	0.333117689
307	31	7	1.0041228	1.9958772	0.815789474	0.3347076	30.87271796	3.507229804	-27.36548816	1	1.50E-09	-2.818332244	0.225806452
308	9	1	1	1.999985951	0.9	0.333334894	9	0.500003512	-8.499996488	0.999983064	0.000355651	-0.875402406	0.111111111
309	13	2	0.768493637	2.231260467	0.866666667	0.256185544	16.91621032	0.896354338	-16.01985598	0.99999994	1.25E-06	-1.649861914	0.153846154
310	28.06855261	25.84462142	0.851015199	1.394199476	0.520625118	0.379035114	32.98243396	18.53724798	-14.44518598	0.987686276	0.024152975	-1.487688916	0.920767871
311	34	10.00006635	0.768535634	2.231457301	0.772726108	0.256179148	44.23997862	4.481406093	-39.75857252	1	1.11E-12	-4.094678168	0.294119599
312	6.498752162	28.50019723	0.23180302	2.76816878	0.185684207	0.0772684	28.0356665	10.29568625	-17.73998026	0.989673576	0.032938672	-1.827015037	4.385487632
313	13	15	0.768614226	2.23119547	0.464285714	0.256220995	16.91355632	6.722853378	-10.19070294	0.99518938	0.013868174	-1.049525831	1.153846154
314	14.9589693	8	1.002767032	1.995635133	0.651552302	0.334433801	14.91769157	4.008748828	-10.90894275	0.99952516	0.001952104	-1.123496314	0.534796204
315	16	15	0.923151074	2.076848926	0.516129032	0.307717025	17.33194106	7.222480081	-10.10946097	0.995445835	0.012362918	-1.041158837	0.9375
316	10	3	0.999790172	2.000163759	0.769230769	0.333268508	10.00209872	1.499877191	-8.50222153	0.999787789	0.001644932	-0.87563156	0.3
317	11.00045702	6	0.8757478	2.1242522	0.647068312	0.291915933	12.5612157	2.824523379	-9.736692319	0.999502801	0.00254315	-1.00276793	0.545431884
318	44.89409444	1	0.999988053	1.9319115	0.978210704	0.34107173	44.89463081	0.517622055	-44.37700875	1	-1.11E-15	-4.570324269	0.022274645
319	10	21.00120662	0.806208319	2.082530392	0.32256809	0.279086619	12.40374201	10.08446585	-2.319276159	0.774258422	0.357530353	-0.238858914	2.100120662
320	12	3	0.768306081	2.085750758	0.8	0.269197891	15.61877524	1.438331013	-14.18044423	0.999997696	2.80E-05	-1.460423544	0.25
321	19	9	1	1.999381374	0.678571429	0.333402084	19	4.501392339	-14.49860766	0.999957244	0.000197801	-1.493190738	0.473684211
322	25.48942258	0	0.815363811	2.068328998	1	0.282749885	31.26141024	0	-31.26141024	1	4.57E-14	-3.219567652	0
323	12	8	0.768493637	2.000091364	0.6	0.27757632	15.61496337	3.99981728	-11.61514609	0.999433432	0.002521771	-1.196227181	0.666666667
324	26	2.000131417	0.999902464	2.000097536	0.92856707	0.333300821	26.00253619	1.000016939	-25.00251925	1	6.84E-11	-2.574973477	0.076928131
325	6	12	0.809989136	2.079593609	0.333333333	0.280313529	7.407506756	5.770358184	-1.637148572	0.781971715	0.391910826	-0.168607575	2
326	10	10	0.768604449	2.231392022	0.5	0.256201784	13.01059345	4.481507464	-8.529085982	0.995162877	0.016504451	-0.878398291	1
327	15	18	0.999490399	2.000403613	0.454545455	0.333175237	15.00764791	8.998184108	-6.009463806	0.949216455	0.099552869	-0.618906029	1.2
328	6	9	0.803191688	2.067497133	0.4	0.279790579	7.470196832	4.353089471	-3.117107362	0.903790667	0.221388422	-0.321026401	1.5
329	22	2	1.624204735	1.233645471	0.916666667	0.568330954	13.54509042	1.621211318	-11.9238791	0.999975204	0.00023012	-1.228023149	0.090909091
330	10.00440347	4.000348546	0.886683003	2.002755296	0.714357774	0.306870371	11.28295392	1.997422528	-9.285531394	0.999691534	0.002019616	-0.956303516	0.399858778
331	20	7	1.559751243	1.440182686	0.740740741	0.519928532	12.8225575	4.860494485	-7.962063017	0.994585014	0.016290303	-0.820001412	0.35
332	20.99138228	16.00072639	1	2	0.567455683	0.333333333	20.99138228	8.000363196	-12.99101908	0.998997686	0.002893857	-1.33792636	0.762252156

333	16.00186824	5	0.999988051	1.999920139	0.761925942	0.333339552	16.00205946	2.50009983	-13.50195963	0.999989735	7.25E-05	-1.390547392	0.312463515
334	28.00340534	2	0.999985875	1.926653476	0.9333409	0.341684012	28.0038009	1.038069391	-26.96573151	1	1.71E-11	-2.777161882	0.071419885
335	30	1.000021839	1.633864109	1.233309456	0.967741254	0.569851832	18.36138014	0.810844216	-17.55053592	0.999999973	6.55E-07	-1.807504438	0.033334061
336	23	3.000347448	0.932705867	2.066059943	0.884603563	0.311029912	24.65943533	1.452207357	-23.20722797	1	1.95E-09	-2.390079011	0.130449889
337	8	2	0.768506847	2.231493153	0.8	0.256168949	10.40979665	0.896260872	-9.513535773	0.999963447	0.000498267	-0.979785358	0.25
338	31.00286094	67.10912121	0.986999321	2.013000679	0.315994645	0.328999774	31.41122824	33.33785324	1.926624999	0.438571554	0.644581485	0.198420336	2.164610593
339	41	2	1.000096845	1.999903155	0.953488372	0.333365615	40.99602974	1.000048425	-39.99598132	1	-3.77E-15	-4.119128558	0.048780488
340	25.01173485	10	0.999788073	2.000137991	0.714381477	0.333270904	25.01703664	4.999655046	-20.0173816	0.99999914	4.60E-06	-2.061561324	0.39981233
341	15	11	0.913627278	2.086372722	0.576923077	0.304542426	16.41807371	5.272308195	-11.14576552	0.998979254	0.003577316	-1.147886351	0.733333333
342	8	6	0.80328298	1.428487415	0.571428571	0.359930834	9.95913047	4.200247015	-5.758883455	0.970617482	0.087548517	-0.593099119	0.75
343	18	10.00061021	0.99946151	1.999390372	0.642843133	0.333281386	18.00969803	5.001829732	-13.0078683	0.999803176	0.000783257	-1.339661637	0.555589456
344	13	5	0.884972305	1.995387406	0.722222222	0.307243676	14.68972523	2.505779071	-12.18394616	0.999946868	0.00035034	-1.254807081	0.384615385
345	18.0008544	4.90E-05	0.999999999	1.998513468	0.999997279	0.333498585	18.00085441	2.45E-05	-18.0008299	1	2.61E-09	-1.853879567	2.72E-06
346	29	1	1.75155112	1.246964472	0.966666667	0.584139407	16.55675342	0.801947467	-15.75480595	0.999999901	2.21E-06	-1.622564792	0.034482759
347	22.74591301	6.001023454	0.768615968	2.231362948	0.791246505	0.256207123	29.59333915	2.689398181	-26.90394097	0.999999999	5.30E-09	-2.770798164	0.263828647
348	24	3.000438827	0.999975672	1.992806032	0.888874442	0.334129172	24.00058389	1.50563516	-22.49494873	1	3.44E-09	-2.316722397	0.125018284
349	17	0	1	2	1	0.333333333	17	0	-17	1	7.74E-09	-1.750805535	0
350	7	2.000043436	0.913444978	2.086555022	0.777774024	0.304481659	7.663296829	0.958538555	-6.704758274	0.999515051	0.00471033	-0.690513406	0.285720491
351	27	1	1	2	0.964285714	0.333333333	27	0.5	-26.5	1	2.49E-12	-2.729196864	0.037037037
352	61.6194577	1	1	1.99847614	0.984030523	0.333502737	61.6194577	0.500381256	-61.11907645	1	1.11E-15	-6.294565727	0.01622864
353	26	0	0.768493637	2.231506363	1	0.256164546	33.83242064	0	-33.83242064	1	-2.22E-16	-3.484352314	0
354	5	1	0.91350489	2.08649511	0.833333333	0.30450163	5.473424447	0.479272631	-4.994151816	0.999202854	0.011721492	-0.514340509	0.2
355	8	1	0.803684611	2.122659957	0.888888889	0.274637724	9.954153522	0.471107017	-9.483046506	0.999991111	0.000220179	-0.976645313	0.125
356	19.02391483	4.0002221	0.999940815	1.997071641	0.826259629	0.333645866	19.02504082	2.003043866	-17.02199696	0.999999833	1.71E-06	-1.753070971	0.210273339
357	55.00045928	1	0.99999391	1.992915416	0.982143004	0.334121018	55.00079424	0.501777442	-54.4990168	1	1.11E-16	-5.612775311	0.018181666
358	0	3	0.000956524	1.295314332	0	0.000737905	0	2.31604015	2.31604015	0.997787919	0.002212081	0.238525642	0
359	1.000021743	1.00008697	1	2	0.499983694	0.333333333	1.000021743	0.500043485	-0.499978258	0.888876282	0.555565482	-0.051492041	1.000065226
360	69.03411944	1	0.999991055	1.990371838	0.985721245	0.334404583	69.03473696	0.502418684	-68.53231827	1	-1.33E-15	-7.058044835	0.014485591
361	0	3	0.000252357	2.999725605	0	8.41E-05	0	1.000091473	1.000091473	0.999747663	0.000252337	0.102997982	0
362	20.5	15.50015363	0.913060569	2.08690425	0.569442014	0.304357092	22.45196069	7.42734298	-15.02461771	0.999692749	0.000988938	-1.547363756	0.756105055
363	8.001929958	10.00010862	0.768528947	2.231407189	0.444501323	0.256181769	10.41200853	4.481525681	-5.930482853	0.977368465	0.064688015	-0.610771895	1.249712091
364	11.99724715	0	1	1.92687081	1	0.341661817	11.99724715	0	-11.99724715	1	2.67E-06	-1.235579219	0
365	0	0	0	1.294639638	0	0	0	0	0	0	0	0	0
366	36	2	0.768493637	2.000063508	0.947368421	0.277579113	46.84489012	0.999968247	-45.84492187	1	-4.44E-16	-4.721502529	0.055555556
367	5	2.17E-05	1	1.998526899	0.999995663	0.333497092	5	1.09E-05	-4.999989149	0.999999993	0.00412552	-0.514941687	0.00000434
368	15.00232024	6	0.768493637	2.000192842	0.714317279	0.277566147	19.52172342	2.999710764	-16.52201265	0.999994415	4.01E-05	-1.701578306	0.399938137
369	37.04388266	6.000021921	0.76861897	2.230786273	0.860606932	0.256257127	48.19537911	2.689644452	-45.50573466	1	2.22E-15	-4.686570126	0.161970655
370	11	1	0.768536025	2.231463975	0.916666667	0.256178675	14.31292696	0.448136296	-13.86479067	0.99999992	2.86E-06	-1.427914838	0.090909091
371	8	4	0.807602599	1.423985586	0.666666667	0.361895893	9.905862132	2.809017198	-7.096844934	0.992766385	0.031379359	-0.730893847	0.5
372	6	7.000021795	0.768493637	2.231506363	0.461537688	0.256164546	7.807481687	3.136904251	-4.670577436	0.972330312	0.088759993	-0.481016049	1.166670299
373	25	14.00006577	0.851604331	2.075314395	0.64102456	0.29095592	29.3563561	6.745997524	-22.61035858	0.999998679	6.15E-06	-2.328608292	0.560002631
374	30.00570239	7.000021806	0.852403851	1.37997775	0.810839486	0.381836085	35.20127502	5.072561355	-30.12871367	0.999999984	1.18E-07	-3.102912863	0.233289717

375	46.01008502	0	1	1.99119075	1	0.334315022	46.01008502	0	-46.01008502	1	-8.88E-16	-4.738512443	0
376	7	2	1	1.777924482	0.777777778	0.359980988	7	1.124907172	-5.875092828	0.998274167	0.013277514	-0.605067356	0.285714286
377	25	2	1.000017679	1.931481991	0.925925926	0.341128361	24.99955803	1.03547432	-23.96408371	1	3.34E-10	-2.468026495	0.08
378	5.000758972	7.000021701	0.768493637	2.000106325	0.416702805	0.27757482	6.507222349	3.499824791	-3.007397558	0.914493475	0.219491637	-0.309727547	1.39979186
379	9	2	1	2	0.818181818	0.333333333	9	1	-8	0.999870164	0.001371742	-0.823908487	0.222222222
380	40.00211283	0	1	2	1	0.333333333	40.00211283	0	-40.00211283	1	-2.22E-16	-4.119760034	0
381	32.00600464	2.000021808	0.768521746	2.231478254	0.941186254	0.256173915	41.64619261	0.896276629	-40.74991598	1	0	-4.196775204	0.062488956
382	31.00361074	17	1.660955112	1.252531897	0.645859973	0.57009182	18.66613404	13.57250865	-5.093625389	0.886891786	0.180608503	-0.524585149	0.548323231
383	29	5	0.849461571	2.034312896	0.852941176	0.294565883	34.13927245	2.457832328	-31.68144012	1	2.11E-11	-3.262825926	0.172413793
384	5	1	0.768528837	2.231471163	0.833333333	0.256176279	6.505936749	0.448134852	-6.057801897	0.99971736	0.005206615	-0.6238843	0.2
385	10.0004119	1	0.999779702	2.000220298	0.909094313	0.333259901	10.00261545	0.499944932	-9.502670519	0.999994365	0.0001296	-0.978666362	0.099995881
386	28.25951535	0	0.837818835	1.393687528	1	0.375449897	33.72986399	0	-33.72986399	1	2.13E-12	-3.473790152	0
387	5	24	0.771434759	2.228536997	0.172413793	0.257147341	6.481429491	10.76939716	4.287967667	0.206089787	0.900380021	0.441611619	4.8
388	14	5	1	2	0.736842105	0.333333333	14	2.5	-11.5	0.99993935	0.000380798	-1.18436845	0.357142857
389	13	14.00019677	0.912380309	2.087619691	0.481477973	0.30412677	14.24844428	6.70629657	-7.542147715	0.983858384	0.039989512	-0.776754939	1.076938213
390	19	2	0.768493637	2.231506363	0.904761905	0.256164546	24.72369201	0.896255567	-23.82743644	1	6.95E-10	-2.453953389	0.105263158
391	17	112.5031325	0.86864118	2.043423062	0.131270956	0.298290528	19.57079678	55.05621162	35.48541484	6.65E-06	0.999997733	3.654591808	6.617831324
392	17	0	0.912120747	2.087879253	1	0.304040249	18.63788325	0	-18.63788325	1	1.62E-09	-1.919488775	0
393	26	5	0.995349157	2.004650843	0.838709677	0.331783052	26.12148695	2.494199933	-23.62728701	0.999999999	8.67E-09	-2.433340288	0.192307692
394	13.00864909	2	0.768495742	2.231497193	0.866743503	0.256165851	16.92741857	0.89625925	-16.03115932	0.99999994	1.25E-06	-1.651026028	0.153743866
395	11	4.000087461	0.805465846	2.078290136	0.733329057	0.279311374	13.65669327	1.924701172	-11.7319921	0.999957967	0.00033929	-1.208260983	0.363644315
396	15	8	0.998971804	2.00100004	0.652173913	0.332993727	15.01543882	3.99800092	-11.0174379	0.999590349	0.001728073	-1.134670074	0.533333333
397	20.9516192	1	0.768503151	2.231464915	0.954445274	0.256170444	27.26289302	0.448136107	-26.81475691	1	1.40E-11	-2.761613225	0.047729008
398	11	4	0.999950763	1.99499178	0.733333333	0.333879782	11.00054163	2.005020793	-8.99552084	0.999709862	0.001834804	-0.926435746	0.363636364
399	24	14	0.998432127	2.001567873	0.631578947	0.332810709	24.03768805	6.994516742	-17.0431713	0.999957967	0.000156223	-1.755251686	0.583333333
400	10	8	0.913239119	2.086760881	0.555555556	0.30441304	10.95003465	3.833692722	-7.116341928	0.993136524	0.023250551	-0.732901814	0.8
401	13	13	0.805265278	2.120934286	0.5	0.27519151	16.14374835	6.12937425	-10.0143741	0.995925915	0.012300608	-1.031365977	1
402	23	2	0.848580386	2.035137189	0.92	0.294266121	27.10409099	0.982734732	-26.12135626	1	9.34E-11	-2.69020089	0.086956522
403	0	2	6.07E-05	2.999939342	0	2.02E-05	0	0.666680146	0.666680146	0.999959562	4.04E-05	0.068660429	0
404	27	0	0.768493637	2.231506363	1	0.256164546	35.13366759	0	-35.13366759	1	7.77E-16	-3.618365865	0
405	17.00180729	18.0002399	0.994939704	2.004885131	0.485737511	0.331665933	17.08827905	8.97819013	-8.110088922	0.980630445	0.04219652	-0.835246387	1.058725087
406	25.99217497	1	0.846907654	1.384668822	0.962952226	0.379510926	30.69068373	0.72219435	-29.96848938	1	2.24E-10	-3.086411594	0.038473117
407	8	1	0.768493637	2.231492308	0.888888889	0.256165746	10.40997558	0.448130606	-9.961844977	0.99999525	0.000128883	-1.025956078	0.125
408	9	3	0.999682737	2.000317263	0.75	0.333227579	9.002856276	1.49976209	-7.503094186	0.99945777	0.003846108	-0.772732873	0.333333333
409	11	6.000021778	1	1.995300674	0.647057995	0.3338563	11	3.007076506	-7.992923494	0.998096083	0.008114734	-0.823179688	0.545456525
410	28	14.00058499	0.999652089	2.000305657	0.666657381	0.333222056	28.0097449	6.999222816	-21.01052209	0.999997597	1.03E-05	-2.163843434	0.500020893
411	17.00505342	7.000588222	0.999894493	2.00010327	0.708377375	0.333298413	17.00684776	3.500113383	-13.50673438	0.99996404	0.000192645	-1.391039136	0.411676932
412	8	2	0.803272557	2.080422841	0.8	0.278556659	9.959259697	0.961343031	-8.997916666	0.999924338	0.000924696	-0.926682489	0.25
413	24	4	0.848526874	2.077830776	0.857142857	0.289960072	28.28431335	1.925084586	-26.35922877	1	6.94E-10	-2.714699038	0.166666667
414	5	3.000216847	0.768542972	2.231178022	0.624983059	0.256204818	6.505817086	1.344678379	-5.161138707	0.995168086	0.030273255	-0.531538248	0.600043369
415	30.00496746	8	0.76850948	2.231437343	0.789501201	0.256174367	39.04306745	3.585133154	-35.4579343	1	9.07E-12	-3.651761626	0.266622519
416	23.01089841	14	0.769373963	2.225674509	0.621733041	0.256881974	29.90860041	6.290227949	-23.61837246	0.99999938	3.15E-06	-2.432422191	0.608407362

417	39.00254987	1	1.747695542	1.230434996	0.975001594	0.586843162	22.31655853	0.812720707	-21.50383783	0.999999999	1.61E-08	-2.214649312	0.025639349
418	25	16.00867014	0.804099459	2.122260765	0.609627182	0.274778017	31.0906813	7.543215425	-23.54746587	0.99999832	7.40E-06	-2.425119624	0.640346806
419	5	10.0009986	0.804203327	2.079534422	0.333311144	0.278875334	6.217333147	4.809248886	-1.408084261	0.781010564	0.411624959	-0.145016572	2.00019972
420	11	4.001762019	0.88641126	2.003756571	0.7332472	0.306698888	12.40958966	1.997129829	-10.41245983	0.99988331	0.000830066	-1.072364253	0.363796547
421	3	11.99650304	0.19039192	2.80960808	0.200046637	0.063463973	15.75697116	4.26981369	-11.48715747	0.987442918	0.065481979	-1.183045817	3.998834347
422	18	6.00037092	0.850380157	2.076000227	0.749988409	0.290591121	21.16700378	2.890351765	-18.27665202	0.999999462	4.29E-06	-1.882286089	0.33335394
423	6	1.000021723	0.768493637	2.231506363	0.857140197	0.256164546	7.807481687	0.448137518	-7.359344169	0.999927614	0.001543686	-0.757928265	0.166670287
424	21.90789645	0	1.584357247	1.19970769	1	0.569080565	13.8276241	0	-13.8276241	1	8.43E-06	-1.424087107	0
425	15	3	0.768493637	2.231471163	0.833333333	0.256167551	19.51870422	1.344404557	-18.17429966	0.99999997	4.81E-07	-1.871744968	0.2
426	4.000195262	8	0.80327543	2.080741409	0.333344181	0.278526609	4.979855121	3.844783386	-1.135071735	0.778164269	0.440514734	-0.116899405	1.999902374
427	89.83726374	0	0.809598125	2.074094684	1	0.280750475	110.9652566	0	-110.9652566	1	-3.11E-15	-11.42815209	0
428	8.002666667	18.00811157	0.999487837	2.000512163	0.307667329	0.333162612	8.006767437	9.001750601	0.994983164	0.482388237	0.678732356	0.102471884	2.250263858
429	4	1	0.768493637	2.231506363	0.8	0.256164546	5.204987791	0.448127783	-4.756860008	0.99889695	0.017117903	-0.489902167	0.25
430	3	2	0.768506435	2.231482368	0.6	0.256169768	3.903675838	0.896265204	-3.007410634	0.982880791	0.110129558	-0.309728893	0.666666667
431	23	1.000022274	0.999971859	1.996836756	0.958332444	0.333678919	23.00064726	0.500803218	-22.49984405	1	1.78E-10	-2.317226559	0.043479229
432	23.00734912	4	0.768629907	2.231334863	0.851892165	0.256212978	29.93293508	1.792648905	-28.14028618	1	1.43E-10	-2.898127577	0.173857491
433	33.99605321	2	1.764670402	1.23191583	0.944438353	0.588893583	19.26481748	1.62348754	-17.64132994	0.999999857	1.79E-06	-1.816855183	0.058830359
434	16	0	0.768493637	2.231506363	1	0.256164546	20.81995116	0	-20.81995116	1	3.44E-10	-2.144216809	0
435	19.99841335	11.00128275	0.899161378	2.100838622	0.645116432	0.299720459	22.24118366	5.236614861	-17.0045688	0.999984011	7.38E-05	-1.75127607	0.550107779
436	0	2	0.000294589	1.294819548	0	0.000227461	0	1.544616779	1.544616779	0.999545129	0.000454871	0.159077859	0
437	10	1	1	2	0.909090909	0.333333333	10	0.5	-9.5	0.999994355	0.000129836	-0.978391329	0.1
438	9	1	0.768493637	2.000092228	0.9	0.277576234	11.71122253	0.499976944	-11.21124559	0.999997285	7.34E-05	-1.154630049	0.111111111
439	8	1	0.768498033	2.231501967	0.888888889	0.256166011	10.40991603	0.448128666	-9.961787368	0.99999525	0.000128884	-1.025950145	0.125
440	45	3.000109376	1	2	0.937497864	0.333333333	45	1.500054688	-43.49994531	1	-1.33E-15	-4.479996767	0.066669097
441	15	3	0.803364322	2.122964103	0.833333333	0.274529788	18.67147891	1.413118571	-17.25836034	0.999999912	1.27E-06	-1.777413695	0.2
442	57	4	1.752647419	1.232810677	0.93442623	0.58706147	32.52222858	3.244618233	-29.27761035	1	1.10E-09	-3.015258957	0.070175439
443	29	2	1.612860834	1.249403596	0.935483871	0.563491205	17.98047258	1.600763762	-16.37970882	0.999999526	5.76E-06	-1.68692264	0.068965517
444	6	13	0.998164975	2.001835025	0.315789474	0.332721658	6.01103039	6.494041636	0.483011246	0.545368744	0.646039049	0.049744633	2.166666667
445	29	6	1.762364284	1.236657877	0.828571429	0.587646303	16.45516779	4.851786507	-11.60338128	0.999414853	0.002193365	-1.19501554	0.206896552
446	31	1	1.618518878	1.248902916	0.96875	0.564450923	19.15331382	0.800702751	-18.35261107	0.999999989	2.90E-07	-1.890109003	0.032258065
447	8	3	0.804182981	2.122151931	0.727272727	0.274808935	9.947984708	1.413659388	-8.53432532	0.999721179	0.002325681	-0.878937883	0.375
448	7	0	0.768493637	2.231506363	1	0.256164546	9.108728635	0	-9.108728635	1	7.24E-05	-0.938094854	0
449	5	2	0.803198574	2.123150476	0.714285714	0.274471213	6.225110655	0.94199635	-5.283114305	0.997711283	0.019507902	-0.544100339	0.4
450	16	4.000282978	0.885444665	2.002105493	0.799988681	0.306642176	18.07001683	1.998038061	-16.07197877	0.999999233	7.61E-06	-1.655229964	0.250017686
451	21.00495318	22.00663721	0.999767887	2.000232113	0.48835565	0.333255962	21.00982984	11.00204174	-10.00778809	0.988226784	0.025190794	-1.030687694	1.047687992
452	14	1	1.633171503	1.233572967	0.933333333	0.569695527	8.57227791	0.810653303	-7.761624608	0.999783906	0.002664404	-0.799358549	0.071428571
453	21	10.00015277	0.76876752	2.230769815	0.677416016	0.256295366	27.31645062	4.48282593	-22.83362469	0.999999841	1.04E-06	-2.351602147	0.476197751
454	21	10.00108859	0.768493637	2.022344382	0.677395568	0.275363039	27.3261859	4.945294519	-22.38089139	0.999999381	3.68E-06	-2.304975796	0.476242314
455	32.02384876	5.000131641	0.768515552	2.231221756	0.864948836	0.256194284	41.6697472	2.240983725	-39.42876347	1	1.37E-14	-4.060711608	0.156137748
456	14	7.00076654	0.768493638	2.231506361	0.666642333	0.256164546	18.21745726	3.137237994	-15.08021926	0.999985887	9.09E-05	-1.55309008	0.500054753
457	12.50867416	7.5	1.000038414	1.774034944	0.62516257	0.360494582	12.50819366	4.227650659	-8.280543003	0.994969642	0.016411751	-0.852801207	0.599583929
458	3	8	0.768893386	2.230535208	0.272727273	0.256346621	3.901711283	3.586583154	-0.315128129	0.69569002	0.564284737	-0.032454593	2.666666667

459	15	7.000347237	1.000169532	1.999830468	0.681807421	0.333389844	14.99745745	3.50047034	-11.49698711	0.999816356	0.0008808	-1.184058157	0.466689816
460	30	6.00022059	0.850563303	2.033500255	0.833328227	0.294918363	35.27074341	2.95068593	-32.32005748	1	3.21E-11	-3.328596208	0.200007353
461	22	22.00480858	0.768641748	2.230955625	0.499945363	0.256248307	28.62191658	9.863400386	-18.7585162	0.999857577	0.000448499	-1.931912588	1.000218572
462	38.00076252	0	1.605872063	1.211014983	1	0.570087489	23.66363012	0	-23.66363012	1	5.33E-10	-2.437083212	0
463	10.00106224	2.000238696	0.768493637	2.000073226	0.833331511	0.277578139	13.01385171	1.000082732	-12.01376898	0.999993247	0.000100352	-1.237280778	0.200002625
464	16	5.000196036	0.807031353	2.119329455	0.76189765	0.275779853	19.82574771	2.359329279	-17.46641843	0.999999446	5.09E-06	-1.798841298	0.312512252
465	25	15	0.844663991	2.039210197	0.625	0.292892108	29.59756812	7.355789033	-22.24177909	0.999996876	1.35E-05	-2.29064882	0.6
466	13.00076051	19	0.998365523	2.001634477	0.406264111	0.332788508	13.02204474	9.492242573	-3.529802169	0.857185441	0.240520313	-0.363529246	1.461452965
467	13	21.00621037	0.878761646	2.005231463	0.382283114	0.304703102	14.79354505	10.47570356	-4.317841494	0.877521801	0.210342184	-0.444688282	1.615862336
468	18.7547315	57.87015228	0.774338386	2.221911179	0.244760326	0.258435878	24.22033032	26.04521406	1.82488374	0.451753929	0.649221668	0.18794215	3.085629473
469	32.00626667	5	0.850437573	1.384229763	0.864887749	0.380565626	37.63505716	3.612117101	-34.02294006	1	1.64E-09	-3.503973635	0.156219407
470	42.06599404	4.000162938	1.590421073	1.200750641	0.913164822	0.569804095	26.44959548	3.33138522	-23.11821026	0.999999956	3.63E-07	-2.380911206	0.095092557
471	13.00362429	10.00026134	0.810685243	2.076634971	0.565279471	0.280774276	16.0402874	4.815608655	-11.22467875	0.998974463	0.003879278	-1.156013511	0.769036471
472	11	32	0.769476098	2.229417964	0.255813953	0.256586622	14.2954408	14.35352209	0.058081294	0.57553983	0.562742031	0.005981709	2.909090909
473	2	2	0.768493637	2.231506363	0.5	0.256164546	2.602493896	0.896255567	-1.706238329	0.94567971	0.272163006	-0.17572303	1
474	18	2	1	2	0.9	0.333333333	18	1	-17	0.999999988	2.30E-07	-1.750805535	0.111111111
475	32.00696509	10.00006555	0.850726063	2.032968909	0.761943051	0.2950125	37.62311567	4.918946623	-32.70416904	1	5.53E-10	-3.368155306	0.312434044
476	6	0	0.803283049	2.123057042	1	0.274500921	7.46934721	0	-7.46934721	1	0.000427822	-0.76925732	0
477	7	21.00624945	0.897203278	2.102796722	0.249944214	0.299067759	7.802022321	9.989671957	2.187649636	0.368577988	0.776884379	0.225302888	3.000892779
478	16.00179958	4.000130316	0.999805468	1.92728683	0.800012782	0.341569505	16.00491304	2.075524127	-13.92938891	0.999995832	3.54E-05	-1.434567718	0.249980029
479	27.07905696	8.000047101	0.768496734	2.231492871	0.771942662	0.256166466	35.23639821	3.585065051	-31.65133316	1	2.66E-10	-3.259725253	0.295433002
480	44.0044491	1.000087427	0.999978868	1.998628717	0.977778075	0.333481071	44.00537904	0.500386799	-43.50499224	1	6.66E-16	-4.480516543	0.022726962
481	16.00392318	0	0.768493637	2.086500362	1	0.26917522	20.8250562	0	-20.8250562	1	7.66E-10	-2.144742568	0
482	3.998851173	1	0.768682634	2.231262888	0.799954036	0.256232198	5.202213497	0.448176683	-4.754036814	0.998879687	0.017220914	-0.48961141	0.250071822
483	14	2	0.805771481	2.120554316	0.875	0.275352622	17.37465315	0.943149621	-16.43150353	0.999999953	9.55E-07	-1.692256902	0.142857143
484	9	2	0.76860176	2.231380601	0.818181818	0.256202093	11.70957506	0.89630608	-10.81326898	0.999989718	0.000154998	-1.113643011	0.222222222
485	29	10	0.768563926	2.00014332	0.743589744	0.277589451	37.73271036	4.999641725	-32.73306863	1	2.03E-09	-3.371131633	0.344827586
486	4	1.000021682	0.768493637	2.231506363	0.799996531	0.256164546	5.204987791	0.4481375	-4.756850292	0.998896901	0.017118295	-0.489901166	0.250005421
487	6	2	0.805216025	2.078417328	0.75	0.279236618	7.451416528	0.962270653	-6.489145875	0.999199747	0.007695943	-0.668307795	0.333333333
488	38.98015184	6	0.76835067	2.086883397	0.866607831	0.269102516	50.73224165	2.875100741	-47.85714091	1	3.55E-15	-4.928738071	0.15392449
489	23	23.00713397	0.768578328	2.231421671	0.499922469	0.256192776	29.92538191	10.31052726	-19.61485465	0.999893569	0.000334153	-2.020105653	1.000310173
490	14.0040608	15	0.768493637	2.231506363	0.482831039	0.256164546	18.22274138	6.721916751	-11.50082463	0.997683906	0.007107968	-1.184453378	1.071117886
491	42	57.00995484	0.97877533	2.0170401	0.424199769	0.326714163	42.91076687	28.26416531	-14.64660155	0.983754454	0.026660728	-1.508432416	1.357379877
492	10.97921363	0	0.768493637	2.086500362	1	0.26917522	14.28666822	0	-14.28666822	1	8.30E-07	-1.471363401	0
493	0	9	0.000546434	2.999440347	0	0.000182145	0	3.000559757	3.000559757	0.998361885	0.001638115	0.309023331	0
494	7	9.00015193	0.804104648	2.122232376	0.437495846	0.274781969	8.705334578	4.240888996	-4.464445582	0.953656567	0.12143241	-0.459786826	1.28573599
495	8	15	0.768575381	2.231424619	0.347826087	0.256191794	10.40886841	6.722162994	-3.686705416	0.890782912	0.216593897	-0.379688485	1.875
496	29	8.001552727	0.999921198	2.000078802	0.783750893	0.333307066	29.00228542	4.000618736	-25.00166669	0.999999997	2.52E-08	-2.574885673	0.275915611
497	5	6	0.805741081	2.077771597	0.454545455	0.279430393	6.205467387	2.887709125	-3.317758262	0.942827793	0.167342835	-0.341691149	1.2
498	4	3.000021697	0.768506844	2.231493156	0.5714268	0.256168948	5.204898347	1.344401029	-3.860497317	0.985638529	0.076391397	-0.397587063	0.750005424
499	26	10	0.999684628	1.998163694	0.722222222	0.333467381	26.00820226	5.004594985	-21.00360727	0.999999616	2.13E-06	-2.163131287	0.384615385
500	14.00258079	8	0.999842363	2.000157637	0.636406289	0.333280788	14.00478846	3.99968475	-10.00510371	0.99912298	0.003480407	-1.030411233	0.571323252

501	6.000541876	0	0.768493637	2	1	0.277585481	7.808186802	0	-7.808186802	1	0.000457781	-0.804153922	0
502	26.00831542	18.00067823	0.768553054	2.231395449	0.590977281	0.256188749	33.84062465	8.067004996	-25.77361965	0.999999395	2.70E-06	-2.654387997	0.692112424
503	25	2	0.768493637	2.000049341	0.925925926	0.277580534	32.5311737	0.99997533	-31.53119837	1	2.29E-12	-3.247352743	0.08
504	38	2	1.000023301	1.999976699	0.95	0.3333411	37.99911458	1.000011651	-36.99910293	1	4.44E-15	-3.810484366	0.052631579
505	11	5	0.803963365	2.079620274	0.6875	0.278807021	13.68221548	2.404285081	-11.2779304	0.999877329	0.000797014	-1.161497822	0.454545455
506	19.00561408	1	0.768493637	2.000042319	0.950014031	0.277581238	24.73099731	0.49998942	-24.23100789	1	3.94E-10	-2.495516632	0.052616032
507	15	7	1.000329905	1.769472251	0.681818182	0.361155725	14.99505305	3.955981789	-11.03907127	0.999475269	0.002242714	-1.136898063	0.466666667
508	22.00524837	24.00190102	0.848732207	2.077860911	0.478300626	0.290006903	25.92719845	11.55125489	-14.37594355	0.997847025	0.005313768	-1.480557738	1.090735293
509	10.00574414	1	0.803548255	2.122800793	0.909138357	0.274590707	12.45195179	0.471075761	-11.98087603	0.999999324	2.02E-05	-1.233893181	0.099942592
510	13	8.000021781	1	1.982369856	0.619046977	0.335303818	13	4.035584862	-8.964415138	0.99805146	0.007199442	-0.923232214	0.615386291
511	13	3	0.805398455	2.078304956	0.8125	0.279293097	16.14107889	1.443484024	-14.69759486	0.999998847	1.43E-05	-1.513684144	0.230769231
512	31	2.18E-05	1.578392898	1.19879533	0.999999298	0.568342067	19.64023028	1.82E-05	-19.64021213	1	2.47E-08	-2.022717183	7.03E-07
513	8.003840396	13.00002172	0.768645588	2.231259014	0.381065175	0.256223344	10.41291399	5.82631673	-4.586597261	0.935575501	0.144905046	-0.472367051	1.624223008
514	12	4	0.885450177	2.002420775	0.75	0.306610022	13.55242827	1.997582152	-11.55484611	0.999956516	0.000333898	-1.190016973	0.333333333
515	11.99811345	9.000347547	0.806826891	2.118018599	0.57138061	0.275852825	14.87074065	4.249418561	-10.62132209	0.998954677	0.004180364	-1.093874677	0.750146895
516	14	59	0.804049248	2.108928399	0.191780822	0.276023144	17.41186878	27.97629356	10.56442478	0.065822947	0.963343267	1.088014905	4.214285714
517	4	3	0.212538645	2.787461355	0.571428571	0.070846215	18.82010681	1.076248105	-17.7438587	0.999966811	0.000740479	-1.827414473	0.75
518	5.5	24.5	0.76869801	2.231062857	0.183333333	0.256253096	7.154955433	10.9813132	3.826357767	0.250578014	0.866475259	0.39407108	4.454545455
519	49.01117258	5.000153553	0.999935912	1.993724681	0.907423981	0.334017796	49.01431382	2.507945857	-46.50636796	1	2.00E-15	-4.78962391	0.102020688
520	19.00609677	14	0.999072616	2.000927384	0.57583594	0.333024205	19.02373907	6.996755661	-12.02698341	0.998818089	0.003565234	-1.238641714	0.736605741
521	34.02942835	10	0.848686275	2.035058911	0.772879177	0.294300023	40.09659325	4.913862664	-35.18273058	1	7.53E-11	-3.623418792	0.293863297
522	33	2.19E-05	1.760600993	1.232101764	0.999999336	0.588297982	18.74359956	1.78E-05	-18.74358177	1	2.49E-08	-1.930374513	6.64E-07
523	9.997657673	4	0.803386534	2.12300281	0.714237904	0.274531663	12.4443929	1.884123742	-10.56026916	0.999896607	0.000783082	-1.087586923	0.400093715
524	7	3.000845877	1	1.768674354	0.699940794	0.361183683	7	1.696663872	-5.303336128	0.993970147	0.031128131	-0.546182956	0.428692268
525	7	11.00045694	0.941868707	2.058131293	0.388879017	0.313956236	7.432033728	5.344876188	-2.087157539	0.827229581	0.324380515	-0.214953351	1.571493849
526	31.00609916	25.00930254	0.933344948	2.062829005	0.553528105	0.31151227	33.22040712	12.12378848	-21.09661864	0.999948956	0.00014932	-2.172710394	0.806593
527	12	36	0.917569976	2.082430024	0.25	0.305856659	13.07802163	17.28749566	4.209474029	0.250934778	0.840565367	0.433527672	3
528	17	5	0.768493637	2.004707121	0.772727273	0.277114318	22.12119811	2.494129914	-19.6270682	0.999999799	1.94E-06	-2.021363509	0.294117647
529	17.01387504	5	0.852031568	1.37965227	0.77287052	0.381788654	19.96859702	3.624101602	-16.34449542	0.999963589	0.000221659	-1.683296062	0.29387779
530	7	4.000021774	0.913506586	2.086493414	0.636362377	0.304502195	7.662780003	1.917102516	-5.745677487	0.995243675	0.023499166	-0.591739056	0.571431682
531	7	0	1.627098455	1.233722544	1	0.568752276	4.302136713	0	-4.302136713	1	0.019251387	-0.443070869	0
532	4	3	1	2	0.571428571	0.333333333	4	1.5	-2.5	0.95473251	0.173296754	-0.257471402	0.75
533	14	7	0.913181651	2.086818349	0.666666667	0.304393884	15.33101327	3.354388753	-11.97662451	0.999869733	0.000667487	-1.233455323	0.5
534	13	0	0.768493637	2	1	0.277585481	16.91621032	0	-16.91621032	1	5.81E-08	-1.742176157	0
535	5	2.000021685	1.000000001	1.773327022	0.714283501	0.360577744	4.999999996	1.127835791	-3.872164205	0.989369854	0.062965535	-0.398788619	0.400004337
536	9	7	0.999992975	2.000007025	0.5625	0.333330992	9.000063225	3.499987706	-5.500075518	0.984055348	0.049960331	-0.566444863	0.777777778
537	7	36.09357526	0.999978988	2.000021012	0.162437207	0.333326329	7.000147089	18.04659803	11.04645094	0.010128819	0.996474941	1.137658085	5.156225037
538	8	0	1	2	1	0.333333333	8	0	-8	1	0.000152416	-0.823908487	0
539	10.00123548	0	0.768493637	2.231506363	1	0.256164546	13.01407714	0	-13.01407714	1	1.22E-06	-1.340301076	0
540	13	0	1	1.773294613	1	0.360581957	13	0	-13	1	1.74E-06	-1.338851292	0
541	22	2	1.629781023	1.23365548	0.916666667	0.569169605	13.49874596	1.621198165	-11.8775478	0.999974394	0.000236877	-1.223251555	0.090909091
542	20	12	0.998681148	2.001318852	0.625	0.332893716	20.02641187	5.996046052	-14.03036581	0.99981619	0.000673772	-1.444967184	0.6

543	27	12	1.737193929	1.258153112	0.692307692	0.579964159	15.54230622	9.537789862	-6.004516357	0.945533876	0.102720058	-0.618396499	0.444444444
544	12.00080998	34.01037466	1.059410857	1.922032437	0.260823756	0.355334901	11.32781479	17.69500556	6.367190767	0.116440209	0.935386842	0.655747814	2.834006598
545	2	7.000086742	0.999943861	2.000056139	0.22222008	0.33331462	2.000112285	3.499945129	1.499832844	0.377216934	0.856909995	0.154465626	3.500043371
546	5	3	0.999964858	2.000035142	0.625	0.333321619	5.000175716	1.499973644	-3.500202072	0.980341962	0.087931914	-0.360480774	0.6
547	8.000325239	1	1.768355417	1.231600577	0.888892904	0.589460452	4.524161353	0.811951552	-3.712209801	0.991405063	0.062452754	-0.382315145	0.124994918
548	5	2	0.999626929	1.999720357	0.714285714	0.333281489	5.00186605	1.000139841	-4.001726209	0.993147261	0.045237627	-0.412132023	0.4
549	1	4	0.997992431	2.001256373	0.2	0.332747463	1.002011607	1.998744416	0.996732809	0.462063132	0.867733102	0.102652078	4
550	1	9.00010845	0.913756251	2.086243749	0.099998916	0.304585417	1.094383758	4.314025364	3.219641606	0.142298321	0.973550295	0.331586256	9.00010845
551	30.22330413	8	0.768592056	2.231394733	0.790703599	0.25619848	39.32294629	3.585201616	-35.73774467	1	9.49E-12	-3.680578894	0.264696407
552	30.25	5.75	0.768624682	2.231318907	0.840277778	0.256213045	39.35600913	2.576951229	-36.7790579	1	4.90E-13	-3.787822245	0.190082645
553	0	2	0.000275119	1.29483203	0	0.000212429	0	1.544601889	1.544601889	0.999575187	0.000424813	0.159076326	0
554	0	2	0.000160011	2.999822295	0	5.33E-05	0	0.666706159	0.666706159	0.999893328	0.000106672	0.068663108	0
555	28.04224833	2	0.768629948	2.08644943	0.933427086	0.269214914	36.48341883	0.958566247	-35.52485259	1	2.58E-14	-3.658653444	0.071320957
556	58.16292318	6	0.998461394	2.001524504	0.906488051	0.332822029	58.25255092	2.997714985	-55.25483593	1	-2.22E-15	-5.690616036	0.103158502
557	1.333333333	14.68025869	0.768506249	2.231463322	0.083262602	0.256171348	1.734967457	6.578758677	4.843791219	0.098549395	0.97517533	0.498855087	11.01019402
558	14	7	0.999305079	1.999189948	0.666666667	0.33326888	14.00973567	3.501418165	-10.5083175	0.999596449	0.001823546	-1.082236497	0.5
559	9	4	0.999950714	1.994696681	0.692307692	0.333912672	9.000443595	2.005317419	-6.995126176	0.998327173	0.008936007	-0.720417978	0.444444444
560	29	0	1.749245331	1.2300149	1	0.587140832	16.5785779	0	-16.5785779	1	1.97E-07	-1.70740388	0
561	27.53365656	14.5	0.851410328	1.382835878	0.655038339	0.381072742	32.33888017	10.48569843	-21.85318174	0.999894923	0.000340457	-2.250627739	0.5266282
562	16	2	0.768493637	2.086499514	0.888888889	0.2691753	20.81995116	0.958543238	-19.86140793	0.999999997	6.48E-08	-2.04549782	0.125
563	11.00086847	15	0.883710247	1.99988704	0.423096193	0.306461048	12.44850165	7.500423623	-4.948078032	0.930215926	0.141241936	-0.509595436	1.363528711
564	1.0000869	16	0.199881209	2.800023317	0.05882834	0.06662919	5.003406295	5.714238129	0.710831834	0.685523387	0.690283142	0.073207548	15.99860972
565	23	3	0.768359521	2.086224603	0.884615385	0.269166886	29.93390383	1.438004324	-28.49589951	1	8.26E-11	-2.934751682	0.130434783
566	9	3	0.913263421	2.086736579	0.75	0.30442114	9.854768942	1.437651513	-8.417117429	0.999763733	0.001898665	-0.866866811	0.333333333

SUPPLEMENTARY TABLE 2. dN/dS analysis showing selection of codons across the H1 HA proteins of pandemic viruses circulating since 2009.

	Observed S Changes	Observed NS Changes	E[S Sites]	E[NS Sites]	Observed S. Prop.	P{S}	dS	dN	dN-dS	P{S leq. observed}	P{S geq. observed}	Scaled dN-dS	dN_dS
1	0	0	0	3	0	0	0	0	0	0	0	0	0
2	9	20	0.859372329	2.030147772	0.310344828	0.297410054	10.47275983	9.851499619	-0.621260209	0.647590436	0.509045209	-0.107127678	0.940678463
3	1	15	0.999874998	1.9998363	0.0625	0.333323743	1.000125017	7.500613927	6.500488909	0.013704578	0.998477211	1.120918854	7.499676338
4	0	14.00231096	0.19052405	2.809459008	0	0.063508375	0	4.983988347	4.983988347	0.399014533	0.600985467	0.859419435	0
5	9	2.000037905	1.777637647	1.222090562	0.818178999	0.59259957	5.062899077	1.636570944	-3.426328132	0.972899035	0.109375963	-0.590822607	0.323247791
6	2	36.00533618	0.997020072	2.002526741	0.052624189	0.332390236	2.005977668	17.97995275	15.97397508	4.16E-05	0.999995732	2.754489714	8.963186895
7	8	10	0.999765293	2.000234707	0.444444444	0.333255098	8.001878094	4.999413302	-3.002464791	0.892533286	0.223043047	-0.517733272	0.624779988
8	11.02306154	15.00019684	1.682669542	1.316524769	0.423584986	0.561040522	6.550936629	11.39378248	4.842845848	0.112885455	0.945503711	0.835081375	1.739260066
9	19.00102967	3	1.777159022	1.222378981	0.863642746	0.592477582	10.69180047	2.454230682	-8.237569791	0.998748677	0.00624961	-1.420454279	0.229543255
10	30	18	0.778513338	2.000946685	0.625	0.280095174	38.53498526	8.995741935	-29.53924332	0.999999853	6.64E-07	-5.093631452	0.233443503
11	5	6	0.999561012	2.000399423	0.454545455	0.333191398	5.002195903	2.999400984	-2.002794918	0.878128055	0.288641989	-0.345354113	0.599616857
12	7	17.01595017	0.781337719	2.218223077	0.291472956	0.260484042	8.95899408	7.670982395	-1.288011686	0.726540006	0.440193131	-0.222099692	0.856232555
13	5	24.00073941	0.999942631	2.000002135	0.172409397	0.333320347	5.000286861	12.0003569	7.000070038	0.045136631	0.983897128	1.207064667	2.39993369

14	27.00333448	19.00554515	0.999490476	2.000509524	0.586915715	0.333163492	27.01710034	9.500352244	-17.5167481	0.999881859	0.000362169	-3.020519453	0.351642187
15	7	29.01105922	1	1.999966214	0.194384729	0.333337087	7	14.50577465	7.505774652	0.051235857	0.978556767	1.294266391	2.072253522
16	5	26.00132959	0.778989385	2.220783714	0.161283405	0.259682769	6.418572701	11.70817735	5.289604649	0.146949882	0.933815943	0.912118713	1.824109174
17	5.000113856	13.00079645	1	1.999900449	0.277770056	0.333344395	5.000113856	6.500721802	1.500607946	0.41216847	0.768979528	0.258758958	1.300114755
18	2	5.000113636	0.778635407	2.221346716	0.285709648	0.259546682	2.568596267	2.250937956	-0.317658311	0.736333949	0.578541733	-0.054775755	0.876329996
19	7.006329114	3	0.999322292	2.000643801	0.700189753	0.333111196	7.011080578	1.499517304	-5.511563274	0.996608353	0.019577067	-0.950392389	0.213878201
20	13	1	1.590823197	1.188490904	0.928571429	0.572379781	8.171869774	0.841403158	-7.330466615	0.999594883	0.004642346	-1.264036959	0.102963359
21	10	2	0.77861491	2.082783105	0.833333333	0.272109964	12.84331942	0.960253612	-11.88306581	0.999994545	8.33E-05	-2.049069338	0.074766778
22	1	9.000189595	0.189494687	2.810505313	0.099998104	0.063164896	5.277192817	3.20233858	-2.074854237	0.871859404	0.479253707	-0.35777974	0.606826146
23	6	0.000151561	0.999999997	1.99998215	0.99997474	0.333335316	6.000000015	7.58E-05	-5.999924234	0.999999986	0.00137223	-1.034603441	1.26E-05
24	11	6.000945477	0.778513338	2.000180319	0.647022838	0.280172424	14.12949459	3.000202243	-11.12929235	0.999673047	0.001765426	-1.919091594	0.212336133
25	2	1.000113602	0.778524585	2.221475415	0.666641423	0.259508195	2.568961905	0.450202417	-2.118759488	0.982520343	0.167092872	-0.36535059	0.175246825
26	20	9.000421154	1	2	0.689645157	0.333333333	20	4.500210577	-15.49978942	0.999980567	9.42E-05	-2.672722996	0.225010529
27	3	2	0.778683774	2.221316226	0.6	0.259561258	3.852655085	0.900367078	-2.952288007	0.982017623	0.113855722	-0.509080984	0.233700411
28	9	3.000037994	0.778513338	2.221486662	0.749997625	0.259504446	11.56049558	1.350464104	-10.21003147	0.999946587	0.000530183	-1.760577848	0.116817146
29	4	3.000416793	1.000210819	1.778569212	0.57139455	0.359946022	3.999156901	1.686983432	-2.312173469	0.937473381	0.216598744	-0.398702139	0.42183477
30	7	3	1	2	0.7	0.333333333	7	1.5	-5.5	0.996596047	0.019661637	-0.948398464	0.214285714
31	4	6	0.778601279	2.22136294	0.4	0.259536855	5.13741771	2.701044431	-2.436373279	0.910236356	0.246812989	-0.420118668	0.525759162
32	9	0	1	2	1	0.333333333	9	0	-9	1	5.08E-05	-1.551924759	0
33	11	7	0.999215575	2.000766577	0.611111111	0.33307384	11.00863545	3.498659004	-7.509976443	0.99610928	0.014343813	-1.294990931	0.317810415
34	5	2.000151616	0.778524591	2.221475409	0.714270244	0.259508197	6.42240471	0.900370811	-5.522033899	0.998337241	0.015216756	-0.952197903	0.140192163
35	6	0	1	2	1	0.333333333	6	0	-6	1	0.001371742	-1.034616506	0
36	14	18	0.994613984	2.004946525	0.4375	0.331586571	14.07581255	8.977795554	-5.098016998	0.925430964	0.139521024	-0.879082089	0.637817215
37	14	1.000113895	1.777678072	1.221859919	0.933326247	0.592650627	7.875441689	0.818517638	-7.056924051	0.999609065	0.004420991	-1.216868351	0.103932918
38	4	11	0.812192649	2.117592448	0.266666667	0.277219189	4.924939918	5.194578406	0.269638487	0.594156063	0.632568493	0.046495405	1.054749599
39	9	10.00003815	0.859675372	2.02955699	0.473683259	0.29754456	10.46906808	4.927202439	-5.541865641	0.96921021	0.080190011	-0.955617611	0.470643844
40	3	4	0.77852871	2.221420409	0.428571429	0.259513972	3.853422436	1.800649703	-2.052772733	0.920327959	0.263587295	-0.353972092	0.46728583
41	8.000947257	4	0.999834831	2.000165169	0.666692977	0.333278277	8.002268984	1.999834845	-6.002434139	0.996149151	0.018736655	-1.035036239	0.249908476
42	5	3.79E-05	1	2	0.999992424	0.333333333	5	1.89E-05	-4.999981059	0.999999988	0.004115531	-0.862177156	3.79E-06
43	10.00132591	2.000037932	0.999955058	1.999999839	0.833349113	0.333323364	10.00177541	1.000019047	-9.001756359	0.999952921	0.00054392	-1.552227619	0.099984153
44	9	0	1	2	1	0.333333333	9	0	-9	1	5.08E-05	-1.551924759	0
45	10	2	0.778564288	2.221038157	0.833333333	0.259555825	12.84415449	0.900479802	-11.94367469	0.999996706	5.35E-05	-2.059520496	0.070108142
46	6.000018956	1.000018956	1	2	0.857140923	0.333333333	6.000018956	0.500009478	-5.500009478	0.999542722	0.006858916	-0.948400098	0.08333465
47	14	15	0.999871764	2.000128236	0.482758621	0.333290588	14.00179553	7.499519147	-6.502276384	0.968896961	0.068103092	-1.121227079	0.535611246
48	29	10.00877729	0.778513338	2.221486662	0.74342243	0.259504446	37.25048575	4.505441089	-32.74504466	1	3.68E-10	-5.646427282	0.120949861
49	7	11	0.998553432	2.001446568	0.388888889	0.332851144	7.010140646	5.496024813	-1.514115833	0.778076354	0.389786787	-0.261088205	0.784010634
50	16	2.000038515	1.777590055	1.221891848	0.888886987	0.592632365	9.000950448	1.636837595	-7.364112852	0.998912593	0.006965666	-1.269838785	0.181851639
51	3	0	0.812092816	2.117708296	1	0.277183599	3.694159017	0	-3.694159017	1	0.021296223	-0.637006316	0
52	3.81E-05	32	0.779035395	2.220629419	1.19E-06	0.259707482	4.88E-05	14.41032877	14.41027992	6.62E-05	0.999933793	2.484852244	0
53	24	17	0.856379596	2.032760619	0.585365854	0.296413304	28.02495542	8.363011288	-19.66194414	0.999968857	0.000112539	-3.390428657	0.298413009
54	14	7	0.778516753	2.221302687	0.666666667	0.259521204	17.98291424	3.151303981	-14.83161026	0.999983274	0.000105957	-2.557504798	0.175238782
55	16	13.00178762	0.778513338	2.221486662	0.551690131	0.259504446	20.55199214	5.852741697	-14.69925044	0.999799939	0.000778318	-2.534681188	0.284777342

56	19.99299915	24	0.999405248	1.998910496	0.454458653	0.333322216	20.0048971	12.00654059	-7.998356512	0.966445335	0.063634678	-1.379205278	0.600180072
57	4	2	0.812222305	2.076614638	0.666666667	0.28115893	4.924760102	0.963105992	-3.96165411	0.991928211	0.056507375	-0.683132122	0.195564042
58	12	1	1.77696941	1.221306568	0.923076923	0.592663725	6.753070666	0.818795236	-5.93427543	0.998886966	0.011057847	-1.023283218	0.121247841
59	1	0	0.778513338	2.082792833	1	0.2720832	1.284499509	0	-1.284499509	1	0.2720832	-0.221494066	0
60	7.001174242	6	0.812291248	2.076613626	0.538503224	0.281176184	8.619044294	2.889319383	-5.72972491	0.987890275	0.044990742	-0.988011328	0.33522503
61	13.0194436	1	1.778067562	1.2215938	0.928670493	0.592756097	7.322243472	0.818602714	-6.503640758	0.999320404	0.007093688	-1.121462346	0.111796708
62	4	20.00462829	0.888550297	2.00055258	0.166634532	0.307552321	4.501714775	9.999551366	5.49783659	0.096917997	0.964092502	0.948025414	2.221276084
63	19	3.000722269	0.999850094	1.999717676	0.863608011	0.33333139	19.00284864	1.500572958	-17.50227568	0.999999969	4.24E-07	-3.018023885	0.078965685
64	9	22	0.9968265	2.003055124	0.290322581	0.332288612	9.028652431	10.98322244	1.954570013	0.387898716	0.749849028	0.3370384	1.216485243
65	2	25	1	2	0.074074074	0.333333333	2	12.5	10.5	0.001799697	0.999744786	1.810578885	6.25
66	5.000568167	1.00005053	1	1.999988751	0.833342097	0.333334583	5.000568167	0.500028077	-4.50054009	0.998627214	0.01783603	-0.77605551	0.099994253
67	12.00405272	0	1.637937043	1.222955374	1	0.572526612	7.328763199	0	-7.328763199	1	0.001247131	-1.263743229	0
68	4	2	0.778529442	2.221153118	0.666666667	0.259537277	5.137891752	0.900433196	-4.237458556	0.994462544	0.042853589	-0.730690761	0.175253438
69	11	0	1.638639318	1.223029145	1	0.572616758	6.712886647	0	-6.712886647	1	0.002170224	-1.157543888	0
70	9	3	0.999842216	2.000022201	0.75	0.333295802	9.001420281	1.499983349	-7.501436931	0.999456755	0.0038522	-1.293518411	0.16663852
71	3	10	0.812139669	2.076876744	0.230769231	0.281112861	3.6939459	4.814922229	1.120976329	0.480885962	0.75332937	0.193296769	1.303463115
72	17	1	0.778581025	2.082767531	0.944444444	0.272102825	21.83459325	0.480130396	-21.35446285	1	3.29E-09	-3.682279957	0.021989436
73	4	10	0.778550586	2.221438094	0.285714286	0.259517841	5.137752216	4.501588421	-0.636163796	0.713086377	0.511948041	-0.109697594	0.876178576
74	6.995849516	2	0.910009804	2.089990196	0.777675249	0.303336601	7.687663895	0.956942288	-6.730721607	0.999514714	0.004672037	-1.160619279	0.124477644
75	12.00587277	3.81E-05	1	2	0.999996826	0.333333333	12.00587277	1.91E-05	-12.00585371	1	1.90E-06	-2.070242403	1.59E-06
76	12	1	0.999988742	1.999900549	0.923076923	0.333341882	12.0001351	0.500024864	-11.50011023	0.999999373	1.69E-05	-1.983033978	0.04166827
77	0	1	0.000232224	1.280913941	0	0.000181263	0	0.780692573	0.780692573	0.999818737	0.000181263	0.13461957	0
78	13.99859859	3	0.917125426	2.082874574	0.823514863	0.305708475	15.26355959	1.440317164	-13.82324243	0.999998665	1.56E-05	-2.383625796	0.094363124
79	20	1	1.7778249	1.221729571	0.952380952	0.592696321	11.24970181	0.818511743	-10.43119007	0.999983038	0.000261746	-1.79871357	0.072758528
80	11.00155303	0	1	1.929972421	1	0.341300141	11.00155303	0	-11.00155303	1	7.34E-06	-1.897064726	0
81	13	0	0.778513338	2.221486662	1	0.259504446	16.69849361	0	-16.69849361	1	2.42E-08	-2.879422852	0
82	7	3	1.000638643	1.999350086	0.7	0.333547467	6.995532355	1.500487594	-5.495044761	0.996580351	0.01973485	-0.947544002	0.214492267
83	20	6.000190767	0.859269007	2.070511503	0.769225125	0.293287843	23.27559802	2.897926797	-20.37767122	0.999999917	7.19E-07	-3.513845833	0.124504934
84	2	1	0.778394539	2.082670451	0.666666667	0.272064613	2.569391098	0.480152777	-2.089238321	0.979862008	0.181781476	-0.360260075	0.18687415
85	6	6	0.812346812	2.117536526	0.5	0.277262511	7.386007939	2.833481229	-4.55252671	0.974706841	0.085120869	-0.78501988	0.38362824
86	6	17	1.001562559	1.782709584	0.260869565	0.359721503	5.990639272	9.536045663	3.545406391	0.223421983	0.88830637	0.611355995	1.591824383
87	7	9.004405262	0.99912331	2.00087669	0.437379577	0.333041103	7.006142216	4.500229978	-2.505912238	0.873851118	0.262006205	-0.432109694	0.642326382
88	7.001402471	6	0.999887297	2.000045046	0.538511325	0.333303282	7.002191643	2.999932432	-4.00225921	0.965372988	0.103457562	-0.690133907	0.428427639
89	9	7	0.999631087	2.000346301	0.5625	0.333212874	9.003321442	3.499394077	-5.503927364	0.98409749	0.049851934	-0.949075683	0.388678123
90	12	22	0.99992347	1.998943397	0.352941176	0.333433765	12.00091844	11.00581439	-0.995104047	0.669687074	0.468265178	-0.171591845	0.917081009
91	13	22	0.778829167	2.221148238	0.371428571	0.259611678	16.69172207	9.904786911	-6.786935162	0.951274955	0.096976562	-1.170312524	0.593395149
92	6	2	1.000029994	1.778681774	0.75	0.35988979	5.999820038	1.124428231	-4.875391808	0.995714235	0.029213608	-0.840693473	0.187410326
93	0	0	0	1.280795552	0	0	0	0	0	0	0	0	0
94	3	3	1	1.999943625	0.5	0.333339597	3	1.500042282	-1.499957718	0.899856639	0.319628286	-0.258646835	0.500014094
95	3	1.000113628	0.778513338	2.000033813	0.749978695	0.280187197	3.853498526	0.50004836	-3.353450166	0.993835706	0.069501901	-0.578255816	0.129764773
96	5	1.000114478	0.915801131	2.084198869	0.833317434	0.305267044	5.459700617	0.47985559	-4.979845027	0.999190557	0.011860981	-0.858704977	0.087890458
97	7	4.00003791	1	2	0.636361443	0.333333333	7	2.000018955	-4.999981045	0.991176538	0.038629817	-0.862177153	0.285716994

98	6	0	0.812124229	2.117676883	1	0.277194321	7.388032257	0	-7.388032257	1	0.000453634	-1.273963353	0
99	4	6.00003789	0.999958751	2.00001872	0.399998484	0.333322087	4.000165003	2.999990865	-1.000174138	0.786892201	0.440708333	-0.172466112	0.74996678
100	8	33.0125726	0.999457507	2.000150889	0.195062136	0.333195996	8.004342296	16.50504108	8.500698787	0.0392656	0.983262144	1.465827213	2.062010903
101	4	53	0.784058248	2.215941752	0.070175439	0.261352749	5.101661783	23.91759619	18.81593441	0.000244268	0.999951924	3.244546052	4.688197141
102	1	6	1.000060089	1.779753729	0.142857143	0.359757939	0.999939914	3.371252945	2.37131303	0.217546772	0.955902962	0.408899934	3.371455521
103	3	32.00003813	0.779762215	2.220116304	0.085714192	0.259931264	3.847326716	14.41367647	10.56634976	0.009840883	0.997695927	1.822019978	3.746413429
104	2	6	0.778721754	2.220619008	0.25	0.259630971	2.568311452	2.701949312	0.133637859	0.65440597	0.656450911	0.023043989	1.052033354
105	16.00850741	1	0.995869263	1.927432501	0.941205893	0.340665913	16.07490862	0.518824913	-15.55608371	0.999999989	3.84E-07	-2.682430161	0.03227545
106	24.01201076	5.000038348	0.999549801	2.000416248	0.827656491	0.333187038	24.02282581	2.499498968	-21.52332684	0.999999994	6.11E-08	-3.711398202	0.104046834
107	5	0	0.778513338	2.082792833	1	0.2720832	6.422497543	0	-6.422497543	1	0.001491106	-1.107470328	0
108	11	2	0.778513338	2.000093174	0.846153846	0.280181211	14.12949459	0.999953415	-13.12954118	0.999997744	3.60E-05	-2.26400667	0.070770643
109	5	0	1	2	1	0.333333333	5	0	-5	1	0.004115226	-0.862180422	0
110	10.00155256	2	0.999966035	1.929980026	0.833354894	0.341291619	10.00189227	1.036280155	-8.965612118	0.999939587	0.000674705	-1.545995047	0.10360841
111	7	9.002245224	0.778799202	2.22119723	0.437438616	0.259600043	8.98819616	4.052879727	-4.935316433	0.966280816	0.094532922	-0.851026641	0.450911357
112	2	1	0.778592229	2.221407771	0.666666667	0.259530743	2.568738714	0.450164987	-2.118573727	0.982518994	0.167106607	-0.365318558	0.17524748
113	12	10.00080171	0.917090255	2.082909745	0.545434669	0.305696752	13.08486262	4.801361045	-8.28350158	0.994816423	0.016398251	-1.428374577	0.366940119
114	2	28.29545923	0.778513338	2.221486662	0.066016494	0.259504446	2.568999017	12.73717268	10.16817367	0.007369405	0.998705163	1.753360052	4.958029412
115	0	2	0.778513338	2.000147201	0	0.280175764	0	0.999926405	0.999926405	0.518146931	0.481853069	0.172423394	0
116	22.01715324	1.000038525	0.858222108	2.071572767	0.95655254	0.292929077	25.6543767	0.482743614	-25.17163309	1	3.15E-11	-4.340497845	0.018817203
117	14	1	0.8593209	2.070490929	0.933333333	0.29330242	16.29193472	0.482977243	-15.80895747	0.99999999	3.80E-07	-2.726034724	0.029645174
118	14	3	1.775697538	1.223828593	0.823529412	0.591992688	7.884225608	2.451323671	-5.432901937	0.98958502	0.040402569	-0.936828336	0.310914958
119	15.5	7.5	0.889982736	2.001619247	0.673913043	0.307781894	17.41606818	3.746966367	-13.66910181	0.999913594	0.000419233	-2.357046392	0.215144218
120	7	6.000190883	0.859356963	2.070395862	0.538453632	0.293320636	8.14562551	2.898088715	-5.247536795	0.984169744	0.055758648	-0.904864697	0.355784674
121	10	2	0.812378786	1.409187027	0.833333333	0.36567847	12.30952872	1.419258028	-10.89027069	0.999875273	0.001260148	-1.877875635	0.115297511
122	15	2	1.637683372	1.223364179	0.882352941	0.572406905	9.159279658	1.634836163	-7.524443495	0.998958459	0.006811507	-1.297485573	0.1784896
123	8	6	0.77861939	2.221296548	0.571428571	0.259547069	10.27459642	2.701125162	-7.573471258	0.997142689	0.013049538	-1.305939728	0.262893553
124	3	1	1.000120328	1.77845343	0.75	0.359940176	2.999639061	0.562286301	-2.43735276	0.983215002	0.136175983	-0.420287566	0.18745132
125	18	8	1.000934947	1.998955466	0.692307692	0.33365717	17.98318667	4.002090159	-13.98109652	0.999959532	0.000199963	-2.410845538	0.222546217
126	4	1	1	1.778618859	0.8	0.359891029	4	0.562234003	-3.437765997	0.993962528	0.059729276	-0.592794907	0.140558501
127	3.75	6.25	1.000246106	1.778953618	0.375	0.359904363	3.749077329	3.513301268	-0.235776061	0.668789102	0.574544752	-0.040656301	0.93711091
128	3.25	3.750037899	0.778517362	2.221376175	0.464283201	0.259514997	4.174601826	1.688159772	-2.486442054	0.936439731	0.217613077	-0.428752332	0.404388213
129	3	13	0.81283406	2.116914162	0.1875	0.277441609	3.690790221	6.141014235	2.450224013	0.312106687	0.862898591	0.422507035	1.663875177
130	28	13	0.932823387	2.059825218	0.682926829	0.311704951	30.01640008	6.311215089	-23.70518499	0.999999774	1.14E-06	-4.087629278	0.210258894
131	1	10	0.779046362	2.220886074	0.090909091	0.259687969	1.283620653	4.502707328	3.219086675	0.177858237	0.963393106	0.555086701	3.507817764
132	23	1	0.857531014	2.072277243	0.958333333	0.292691855	26.82118736	0.482560914	-26.33862644	1	9.22E-12	-4.54172961	0.01799178
133	3	23	0.187612538	2.812387462	0.115384615	0.062537513	15.9904025	8.178105013	-7.812297492	0.923918409	0.220072417	-1.347121989	0.511438346
134	15	2	0.778654814	2.221345186	0.882352941	0.259551605	19.26399185	0.90035534	-18.36363651	0.999999995	1.27E-07	-3.166553574	0.046737735
135	10	3	1	2	0.769230769	0.333333333	10	1.5	-8.5	0.999787371	0.001647721	-1.465706717	0.15
136	24.03869206	16.03268656	0.856410612	2.032767793	0.599896806	0.296420121	28.06911978	7.887121496	-20.18199828	0.999982814	6.57E-05	-3.480104757	0.280989271
137	8	18	0.999114279	2.000874429	0.307692308	0.333039347	8.00709205	8.996066791	0.988974742	0.483086404	0.678173242	0.170534932	1.123512348
138	7	36.00537429	0.778954556	2.221045444	0.162770354	0.259651519	8.986403572	16.21100297	7.224599398	0.097183113	0.953905917	1.245781631	1.803947802
139	25.02593715	0	1	1.779104575	1	0.359828129	25.02593715	0	-25.02593715	1	8.49E-12	-4.315374608	0

140	0	0	0	1.280795552	0	0	0	0	0	0	0	0	0
141	9	5.00003804	1	2	0.642855396	0.333333333	9	2.50001902	-6.49998098	0.995960334	0.017434144	-1.120831268	0.277779891
142	5	26.02626186	0.778513338	2.221486662	0.1611538	0.259504446	6.422497543	11.71569576	5.293198214	0.146899317	0.933837757	0.912738373	1.824165082
143	5.001022417	2	0.778538125	2.221178777	0.71432744	0.2595372	6.423606321	0.900422794	-5.523183527	0.998335856	0.01522176	-0.95239614	0.140174031
144	16	18.0359996	0.778668313	2.221063559	0.470090498	0.259579305	20.54790175	8.120433801	-12.42746795	0.997693045	0.006545092	-2.142943912	0.395195281
145	28	36	1.002445702	1.864290199	0.4375	0.349681916	27.93168742	19.31029837	-8.62138905	0.943847458	0.091156704	-1.486638569	0.6913402
146	5	22.00360339	0.778604456	2.221355967	0.185160474	0.259538243	6.421745936	9.905482829	3.483736892	0.261004207	0.866917258	0.600721949	1.542490613
147	2	3	0.812137889	2.076778275	0.4	0.281122	2.462635997	1.444545157	-1.018090839	0.860980804	0.432618652	-0.175555598	0.586584927
148	15.00973632	0	1	1.999929035	1	0.333341219	15.00973632	0	-15.00973632	1	7.10E-08	-2.588220158	0
149	8	12.00216401	0.997880551	2.002037351	0.399956725	0.332635953	8.016991608	5.994975071	-2.022016536	0.811202729	0.336184198	-0.348668614	0.747783628
150	27	4	0.999940226	2.000038585	0.870967742	0.333315763	27.00161399	1.999961416	-25.00165257	1	8.75E-10	-4.311187071	0.074068217
151	7	8.999574369	0.999875992	2.000124008	0.437511639	0.333291997	7.000868167	4.499508196	-2.501359971	0.873593048	0.262424262	-0.431324719	0.642707174
152	3	1	1	1.999931859	0.75	0.333340905	3	0.500017036	-2.499982964	0.987653199	0.111117841	-0.431087273	0.166672345
153	18	0	0.778513338	2.082792833	1	0.2720832	23.12099115	0	-23.12099115	1	6.68E-11	-3.98689318	0
154	8	15	0.999977451	2.000022549	0.347826087	0.333325817	8.000180399	7.499915441	-0.500264958	0.651382078	0.519277423	-0.08626373	0.93746829
155	13	20.00811387	0.784633901	2.206313023	0.393842558	0.262336284	16.56823644	9.068574433	-7.499662012	0.968111317	0.06830247	-1.293212351	0.547346995
156	5	18	0.999041505	2.000958495	0.217391304	0.333013835	5.004797075	8.995688837	3.990891762	0.170295285	0.923760693	0.688173748	1.797413302
157	8	8	0.999683552	1.931331271	0.5	0.341070794	8.002532386	4.142220508	-3.860311878	0.942553222	0.141208358	-0.665657065	0.517613714
158	3	25.00262617	0.999355759	2.000268445	0.107132809	0.33316032	3.001933969	12.49963535	9.497701384	0.006116302	0.998708775	1.637746437	4.163860858
159	3	11	0.812369594	2.076535279	0.214285714	0.281203304	3.692900401	5.297285392	1.604384991	0.4147485	0.799456417	0.276653866	1.434451195
160	11	26.07934279	0.802423323	2.197576677	0.296661137	0.267474441	13.70847492	11.8673187	-1.841156221	0.727505283	0.403920907	-0.317481769	0.865692119
161	5	0	0.778513338	2.221486662	1	0.259504446	6.422497543	0	-6.422497543	1	0.001176858	-1.107470328	0
162	2	3.79E-05	0.778513338	2	0.999981052	0.280190607	2.568999017	1.89E-05	-2.568980069	0.999999647	0.078510614	-0.442984864	7.38E-06
163	3	5	0.812080314	2.076533799	0.375	0.281131464	3.694215889	2.407858713	-1.286357176	0.838715718	0.400155469	-0.221814394	0.651791554
164	7	4	0.778558466	2.221441534	0.636363636	0.259519489	8.990975382	1.80063258	-7.190342802	0.998448888	0.009417191	-1.239874558	0.200271106
165	8	2.000115128	1.590921301	1.188676519	0.79999079	0.572356652	5.028532834	1.682640395	-3.34589244	0.968033542	0.126751282	-0.576952591	0.334618556
166	2	10.00003812	0.194316067	2.805683933	0.166666137	0.064772022	10.29250967	3.564206929	-6.728302745	0.961564819	0.180176193	-1.160202179	0.346291336
167	0	1	3.96E-05	1.280836328	0	3.09E-05	0	0.780739879	0.780739879	0.999969097	3.09E-05	0.134627728	0
168	17	4	1.777114021	1.222308769	0.80952381	0.592485337	9.566071618	3.272495544	-6.293576074	0.99078162	0.031768893	-1.085239615	0.342093983
169	5	11.00079636	0.999881883	2.000118117	0.312484447	0.333293961	5.000590655	5.500073353	0.499482698	0.54701488	0.660802908	0.086128841	1.09988474
170	6	4.001099712	0.812162882	2.076783652	0.599934025	0.281127696	7.387680636	1.926584749	-5.461095887	0.992817586	0.034883341	-0.941689991	0.260783437
171	11	15.00432851	0.812272145	2.076568919	0.423006501	0.281175782	13.54225929	7.225538419	-6.316720868	0.961945225	0.085527971	-1.089230612	0.533554872
172	35.01923811	50.08104595	0.99834699	1.932309433	0.411505537	0.340656442	35.0772211	25.9177154	-9.159505701	0.930614606	0.104185863	-1.579429297	0.73887596
173	6.884393064	30.49823638	0.778603376	2.221256997	0.184160215	0.259546539	8.841976894	13.73017009	4.888193194	0.197507459	0.894249093	0.842900894	1.552839399
174	11	3.000189768	1.000060022	1.779677088	0.785703636	0.359767842	10.9993398	1.685805693	-9.313534102	0.999808799	0.001439848	-1.605989352	0.153264262
175	18	2	0.778668695	2.000090764	0.9	0.280221698	23.11637814	0.99995462	-22.11642352	1	1.16E-08	-3.813669471	0.043257409
176	6	5	1.000050471	1.999683553	0.545454545	0.333379714	5.999697189	2.500395621	-3.499301568	0.961342026	0.122154734	-0.60340586	0.416753636
177	24	14.0004985	0.858443475	2.030743364	0.631570662	0.297122866	27.95757754	6.894272682	-21.06330485	0.99999536	2.02E-05	-3.632073812	0.246597641
178	8	6	0.999954492	2.000045508	0.571428571	0.333318164	8.000364078	2.99993174	-5.000432338	0.982571753	0.057601677	-0.862254972	0.374974402
179	5.992469174	30	0.779487638	2.220438852	0.166492306	0.259835579	7.687702644	13.51084268	5.823140034	0.136953456	0.934909933	1.004119466	1.757461664
180	9	45.00827764	0.805433106	1.97430176	0.166641122	0.289751773	11.17411234	22.79706099	11.62294866	0.0279902	0.987744337	2.004215755	2.040167515
181	19	7	0.999921129	1.998913145	0.730769231	0.333436608	19.00149866	3.501903031	-15.49959563	0.999993236	4.00E-05	-2.672689579	0.184296149

182	11	4	0.778899128	2.000104025	0.733333333	0.280280045	14.12249624	1.99989598	-12.12260026	0.999956346	0.000350769	-2.090373721	0.141610658
183	6	9	0.915759131	2.084240869	0.4	0.305253044	6.551941225	4.318118953	-2.233822272	0.858689864	0.293997808	-0.385191566	0.659059476
184	6	4	0.778790642	2.221209358	0.6	0.259596881	7.704252825	1.800820794	-5.903432031	0.995580426	0.023734323	-1.017964703	0.23374373
185	21	9.001258293	0.778521866	2.221460297	0.699970641	0.259508832	26.97419419	4.051955511	-22.92223868	0.999999922	5.55E-07	-3.952621081	0.150215998
186	7	6	0.812198272	2.076706601	0.538461538	0.281144	8.618585192	2.889190026	-5.729395165	0.987896533	0.044978821	-0.987954468	0.335227878
187	15	18.00072199	0.999630066	1.999940446	0.45453551	0.333257732	15.00555107	9.000629009	-6.00492206	0.949097109	0.099751411	-1.035465247	0.599819958
188	1	9	0.812292477	2.076612396	0.1	0.281176609	1.231083666	4.33398164	3.102897975	0.180903525	0.963168279	0.535051577	3.520460681
189	11	10.00080294	0.812485627	2.117313383	0.523789497	0.277317872	13.53870104	4.72334564	-8.815355399	0.995629759	0.014598255	-1.520085367	0.348877313
190	17.33716868	15.66666667	0.999528262	2.000458044	0.525307695	0.333177608	17.34535115	7.831539739	-9.513811409	0.992364906	0.01877902	-1.640524386	0.451506555
191	38.99494642	0	1.00469481	1.99530519	1	0.33489827	38.81272803	0	-38.81272803	1	-1.11E-15	-6.692714843	0
192	15	1	0.999949207	2.000050793	0.9375	0.333316402	15.00076193	0.499987302	-14.50077463	0.999999977	7.66E-07	-2.500456797	0.033330794
193	21.5049738	5	1.77699336	1.22267437	0.811356161	0.592396732	12.10188754	4.089396264	-8.012491279	0.994701325	0.01693634	-1.381642622	0.33791392
194	0	1	0.000238636	1.280995032	0	0.000186255	0	0.780643153	0.780643153	0.999813745	0.000186255	0.134611048	0
195	8	0	1	1.999300315	1	0.333411094	8	0	-8	1	0.0001527	-1.379488675	0
196	9	5	0.915832576	2.084167424	0.642857143	0.305277525	9.827123689	2.399039512	-7.428084178	0.998068109	0.0093925	-1.28086975	0.244124282
197	10	0	0.778513338	2.221486662	1	0.259504446	12.84499509	0	-12.84499509	1	1.38E-06	-2.214940656	0
198	10.05078007	2	0.778547135	2.221452865	0.83403564	0.259515712	12.90966161	0.900311698	-12.00934991	0.999996571	5.44E-05	-2.070845274	0.069739372
199	12	10	0.999617754	1.998787446	0.545454545	0.333383144	12.00458871	5.003033223	-7.001555482	0.98838111	0.032741771	-1.207320811	0.41676007
200	11	35.25403159	0.999694195	2.000069319	0.237817107	0.333257669	11.00336489	17.62640488	6.62303999	0.109020871	0.941012304	1.142051082	1.601910421
201	11.0046643	8.011873731	0.999701213	2.000298787	0.578689154	0.333233738	11.00795334	4.005338494	-7.002614843	0.992548598	0.024129247	-1.207503483	0.3638586
202	5	38.72198227	0.876382144	2.123617856	0.11435895	0.292127381	5.705273703	18.23396905	12.52869535	0.00481716	0.998643992	2.160399168	3.195984978
203	9.0030189	35.02078899	0.999988748	2.000011252	0.204503411	0.333329583	9.003120206	17.51029598	8.507175776	0.045134839	0.979553209	1.466944079	1.944914161
204	14	13.00003804	0.778839979	2.221160021	0.518517788	0.259613326	17.97545116	5.852814706	-12.12263646	0.998965964	0.00357993	-2.090379962	0.325600434
205	2	7	0.812219949	1.411072041	0.222222222	0.365323112	2.462387193	4.960767272	2.498380079	0.302583062	0.896725329	0.430810878	2.01461707
206	4	0	0.812114599	1.409372063	1	0.365572575	4.925413243	0	-4.925413243	1	0.017860534	-0.849318973	0
207	9	15.00214088	0.778945024	2.221026645	0.374966552	0.259650793	11.55408883	6.754597437	-4.799491392	0.931329322	0.145875634	-0.827605502	0.584606674
208	10.00019053	30.10153729	0.999737437	2.000262563	0.249370565	0.333245812	10.00281689	15.04879302	5.045976127	0.169068231	0.904973007	0.870108365	1.504455513
209	4	2.5	0.77853216	2.000080869	0.615384615	0.280187328	5.137873817	1.249949459	-3.887924358	0.986392997	0.077167611	-0.670418452	0.243281463
210	16.00651219	0	0.859321326	1.362165336	1	0.386822636	18.62692303	0	-18.62692303	1	2.54E-07	-3.21195367	0
211	1	5.000037925	0.778524591	2.221475408	0.166665613	0.259508197	1.284480942	2.250773476	0.966292533	0.51151863	0.835139511	0.166623701	1.752282499
212	4.000189631	10.00056868	0.999971271	2.000017997	0.285712355	0.333324949	4.000304557	5.000239343	0.999934786	0.475511734	0.738787246	0.172424839	1.249964664
213	2	7.00068189	0.778532126	2.22142858	0.222205387	0.259514108	2.568937022	3.151432349	0.582495328	0.5739709	0.721982212	0.100443213	1.226745663
214	5	33.70105507	1	1.999696088	0.129195444	0.333367105	5	16.85308847	11.85308847	0.003605417	0.999037043	2.043900162	3.370617693
215	2.000189337	2	0.778513338	2.000045019	0.500023666	0.280186067	2.56924222	0.999977491	-1.569264729	0.930506387	0.313524533	-0.270597865	0.389211061
216	9	13.00794556	0.999877921	2.000122079	0.408943214	0.33329264	9.001098845	6.503575805	-2.49752304	0.836632491	0.293287466	-0.430663094	0.722531317
217	5	3	0.778768704	2.22121013	0.625	0.2595914	6.420391539	1.350615126	-5.069776413	0.994811602	0.031983406	-0.874212393	0.210363358
218	7	1.000113748	0.999796312	2.000203688	0.874987559	0.333265437	7.001426106	0.500005952	-6.501420155	0.999847791	0.002587998	-1.121079434	0.071414872
219	18	19.04001973	0.999275288	1.999491976	0.485960864	0.33322869	18.01305427	9.522428674	-8.490625596	0.982036924	0.038690079	-1.464090231	0.528640426
220	2	33.10942353	1.000025037	1.987453456	0.056964763	0.334738824	1.999949927	16.65921958	14.65926965	0.000103868	0.999988613	2.527787058	8.32981834
221	5	0	1	1.778626789	1	0.359890002	5	0	-5	1	0.006037386	-0.862180422	0
222	2	31	0.885795992	2.003154647	0.060606061	0.306615136	2.257856231	15.47558999	13.21773376	0.0006716	0.99991188	2.279214252	6.854107793
223	6	4	0.778513338	2.000203619	0.6	0.280170075	7.706997051	1.999796401	-5.70720065	0.992970353	0.034298657	-0.984127333	0.259478029

224	8.000644037	7.001515783	0.778537793	2.221444765	0.53329948	0.259514106	10.27650052	3.151784773	-7.124715751	0.994497983	0.02166974	-1.228558086	0.306698254
225	6	4.000038156	0.859376753	2.029635455	0.599997711	0.29746387	6.981803944	1.970816062	-5.010987881	0.989929786	0.045511335	-0.864075129	0.282278918
226	22.00777516	15.00641592	0.858485056	2.030572197	0.594576689	0.297150586	25.63559495	7.390240023	-18.24535492	0.999956343	0.00016408	-3.14615756	0.288280418
227	3	11	0.779242388	2.220678675	0.214285714	0.259754297	3.849893236	4.953440641	1.103547405	0.487232	0.746073217	0.190291393	1.286643638
228	21	6.000114051	0.859314913	2.029725399	0.777774492	0.297439572	24.43807234	2.956121086	-21.48195125	0.999999961	3.51E-07	-3.704263558	0.120963759
229	23.00642048	1	1.000007047	1.999992953	0.958344477	0.333335682	23.00625836	0.500001762	-22.5062566	1	1.75E-10	-3.880890761	0.021733293
230	10	2	0.812240144	2.117185821	0.833333333	0.277269388	12.31162984	0.944650196	-11.36697965	0.999993336	9.92E-05	-1.960077461	0.076728281
231	2	2	0.188369774	2.811630226	0.5	0.062789925	10.61741467	0.711331092	-9.906083576	0.999056416	0.021721648	-1.708166263	0.066996639
232	6	44.02547145	0.999789442	2.000109364	0.1199389	0.333274389	6.001263616	22.01153209	16.01026848	0.000517915	0.999869828	2.760748005	3.66781623
233	2.00536897	17.5147898	0.226687636	2.773282483	0.10273323	0.075563298	8.846397647	6.315544812	-2.530852835	0.821164461	0.43864938	-0.436410353	0.713911477
234	9	0.000151642	0.889006489	2.00013593	0.999983151	0.307706011	10.12366064	7.58E-05	-10.12358483	1	2.47E-05	-1.745671327	7.49E-06
235	12.00359876	4	0.999977474	2.000022526	0.750056218	0.333325825	12.00386915	1.999977474	-10.00389168	0.999883923	0.000792507	-1.725031909	0.166611069
236	4	4	0.811787103	2.077180264	0.5	0.280995595	4.927400284	1.925687467	-3.001712817	0.955574605	0.161058622	-0.517603604	0.390812062
237	19	6.000114469	0.999560439	2.000425363	0.75999652	0.33318839	19.00835532	2.999419313	-16.00893601	0.999997748	1.55E-05	-2.76051824	0.157794784
238	13.0020842	11.00072098	0.936199593	2.06327849	0.541690194	0.312120831	13.88815408	5.331670463	-8.556483618	0.994578204	0.016285593	-1.475446531	0.383900584
239	5.5	154.0810187	0.783433667	2.214663239	0.034465252	0.261310322	7.020377388	69.57311429	62.5527369	2.59E-14	1	10.78634901	9.910167281
240	5	66.15400061	0.8169667	1.404806875	0.070270118	0.367709252	6.120200492	47.09117089	40.9709704	6.83E-09	0.999999999	7.064873706	7.694383698
241	5	4.001553337	0.812220193	2.117109121	0.555459687	0.277271725	6.155966133	1.890102545	-4.265863588	0.982965516	0.073408963	-0.735588813	0.307035891
242	13.99787742	5.000113817	0.99986405	1.999678841	0.736808289	0.333338807	13.9997807	2.500458431	-11.49932227	0.999938792	0.000383354	-1.982898104	0.178606971
243	11	0	0.889003976	2.000132567	1	0.307705767	12.37339798	0	-12.37339798	1	2.34E-06	-2.133620297	0
244	0	5	0.000290622	2.999709378	0	9.69E-05	0	1.666828139	1.666828139	0.999515724	0.000484276	0.287421318	0
245	7.001970443	7	0.778548694	2.221451306	0.500070363	0.259516231	8.993619149	3.151093153	-5.842525995	0.986966198	0.046224824	-1.007462305	0.350369868
246	14	1	0.778513338	2.000056462	0.933333333	0.280184913	17.98299312	0.499985885	-17.48300723	0.999999995	2.04E-07	-3.01470131	0.027803263
247	4	11	0.778513338	2.001115359	0.266666667	0.280078177	5.137998034	5.496934472	0.358936438	0.584283647	0.641862118	0.061893594	1.069859201
248	0	0	0	1.280795552	0	0	0	0	0	0	0	0	0
249	4.000265212	4	0.999933103	2.000027349	0.500016575	0.333315428	4.000532835	1.999972651	-2.000560183	0.912079239	0.258591314	-0.344968764	0.499926568
250	17	6	1.776517878	1.222811029	0.739130435	0.592305123	9.569281688	4.906727087	-4.662554601	0.953631258	0.109374212	-0.803992658	0.512758141
251	6	31	0.9495075	2.050023341	0.162162162	0.316552004	6.319065407	15.12177904	8.802713635	0.027733333	0.989842775	1.517905471	2.393040437
252	28.00611865	21.00225096	0.85904581	2.069834409	0.571455832	0.29330179	32.6014263	10.14682666	-22.45459964	0.999986685	4.57E-05	-3.871983237	0.311238734
253	27	3	1.000518557	1.999449029	0.9	0.333509789	26.98600621	1.500413342	-25.48559287	1	1.69E-10	-4.394635841	0.055599681
254	12	1	0.999977376	1.930183904	0.923076923	0.341270422	12.0002715	0.518085348	-11.48218615	0.999999148	2.22E-05	-1.979943219	0.043172802
255	3	6	0.778760769	2.22121778	0.333333333	0.259588779	3.852274175	2.701220949	-1.151053227	0.815904898	0.426184062	-0.198483111	0.701201635
256	3	1	0.81210296	2.076824551	0.75	0.281108805	3.694112875	0.481504323	-3.212608552	0.993755498	0.070121795	-0.553969639	0.130343695
257	2	1	0.188193896	2.811806104	0.666666667	0.062731299	10.62733726	0.355643299	-10.27169396	0.999753139	0.011311925	-1.771210687	0.033464949
258	5	7	0.999100795	2.000870912	0.416666667	0.333036739	5.004500073	3.498476568	-1.506023506	0.822871077	0.367630814	-0.259692796	0.699066144
259	21.01242048	2	0.778554609	2.221398834	0.913090411	0.259522231	26.98901302	0.900333596	-26.08867942	1	7.23E-11	-4.498629725	0.033359263
260	6.000643744	2	0.812162365	2.117494065	0.750020115	0.277221027	7.388477969	0.944512683	-6.443965286	0.999237302	0.007402066	-1.111172141	0.127835894
261	4	4	1	2	0.5	0.333333333	4	2	-2	0.912056089	0.258649596	-0.344872169	0.5
262	6	6	0.999926856	2.000073144	0.5	0.333308952	6.000438895	2.999890288	-3.000548607	0.933576775	0.177673626	-0.517402853	0.499945144
263	9	1	0.999971331	1.929961901	0.9	0.341294921	9.000258024	0.518144943	-8.48211308	0.999978556	0.000435309	-1.462622366	0.05757001
264	6.000643696	1	0.778513338	2.221486662	0.857155993	0.259504446	7.707823878	0.450149	-7.257674878	0.999920679	0.0016627	-1.251485037	0.058401568
265	16	2	1.776871108	1.222349267	0.888888889	0.592444331	9.004592355	1.63619356	-7.368398795	0.998918077	0.006935564	-1.270577836	0.181706567

266	19	20.0003522	1.036514091	1.955239576	0.487175088	0.34645703	18.33067217	10.22910565	-8.101566518	0.976042608	0.048916107	-1.397002407	0.558032218
267	11	17	0.998942254	2.001035221	0.392857143	0.332983251	11.01164753	8.495602586	-2.516044944	0.81002275	0.312689364	-0.433856938	0.771510581
268	25	1	1.000198174	1.999801826	0.961538462	0.333399391	24.99504663	0.500049548	-24.49499708	1	2.10E-11	-4.223821381	0.020005946
269	6.000492275	2	0.888535641	2.000499488	0.750015383	0.307554461	6.75323757	0.999750318	-5.753487252	0.998477722	0.012884324	-0.992108813	0.148040152
270	6	4	0.778513338	2.000169091	0.6	0.280173556	7.706997051	1.999830923	-5.707166128	0.992969825	0.03430069	-0.98412138	0.259482508
271	3	1	1	2	0.75	0.333333333	3	0.5	-2.5	0.987654321	0.111111111	-0.431090211	0.166666667
272	19	1	0.778513338	2.221408644	0.95	0.259511195	24.40549066	0.45016481	-23.95532585	1	1.11E-10	-4.130762589	0.018445227
273	6	27	0.999834841	1.999996231	0.181818182	0.333297048	6.00099112	13.50002544	7.499034321	0.043477578	0.983285337	1.293104115	2.249632631
274	0	16	0.00418112	2.995744725	0	0.001393741	0	5.340909013	5.340909013	0.977931737	0.022068263	0.920965437	0
275	7	13	0.812354781	2.117473346	0.35	0.277270456	8.616924726	6.13939251	-2.477532216	0.836393381	0.306965449	-0.427215954	0.712480694
276	10	13	0.888672884	2.000485236	0.434782609	0.307588871	11.25273447	6.498423366	-4.754311105	0.935597318	0.137329922	-0.819814791	0.577497264
277	3	20	0.779196109	2.220645248	0.130434783	0.259745772	3.850121897	9.006391281	5.156269384	0.115596862	0.960170463	0.889126902	2.339248346
278	5	23.00297213	0.999830882	2.000169118	0.178552476	0.333276961	5.000845733	11.50051359	6.499667861	0.057174161	0.978880627	1.120777275	2.29971373
279	10	8.002388716	0.999784126	1.930157602	0.55548184	0.341230038	10.00215921	4.14597684	-5.856182365	0.9825852	0.050663416	-1.009817156	0.414508183
280	7	10	0.999831047	1.999988233	0.411764706	0.333297094	7.001182872	5.000029419	-2.001153453	0.828227111	0.32594972	-0.345071065	0.714169235
281	4	4.000416825	0.999841817	2.000158183	0.49997395	0.333280606	4.00063283	2.000050226	-2.000582604	0.912089194	0.258578782	-0.344972631	0.499933463
282	9	2	0.917212723	2.082787277	0.818181818	0.305737574	9.812336629	0.960251689	-8.852084941	0.999943317	0.000675482	-1.526418865	0.097861674
283	10.5	17.49768325	0.916527116	2.083419541	0.37503103	0.305514471	11.45628953	8.398540433	-3.057749095	0.837677055	0.275215208	-0.527266281	0.73309429
284	8	9	0.917075416	2.082924584	0.470588235	0.305691805	8.723382897	4.320847749	-4.402535148	0.954805484	0.114701102	-0.759155922	0.495317906
285	2	7	1.00018101	1.781199097	0.222222222	0.359598822	1.999638045	3.929936868	1.930298823	0.315308611	0.890329628	0.332853171	1.965324114
286	6	14	0.778805368	2.221000328	0.3	0.259618604	7.704107141	6.303465977	-1.400641164	0.754574337	0.421938216	-0.241521078	0.818195524
287	4	10	0.999524834	2.000389505	0.285714286	0.333184458	4.001901566	4.999026428	0.997124862	0.475979096	0.738423556	0.171940307	1.249162766
288	5	16	1.000033353	1.996887428	0.238095238	0.333686949	4.99983324	8.012469696	3.012636456	0.247565669	0.879518746	0.519487234	1.602547387
289	26	30.00342967	0.999180126	2.000819874	0.464257281	0.333060042	26.02133422	14.9955676	-11.02576662	0.985335424	0.028188182	-1.901240023	0.576279735
290	26	23.99953906	0.778758849	2.219646512	0.520004794	0.259724339	33.38645849	10.81232481	-22.57413367	0.999976873	7.65E-05	-3.892595218	0.323853601
291	7	22	0.77859639	2.221332616	0.24137931	0.259538272	8.990537445	9.903964781	0.913427336	0.509333983	0.657317472	0.157507833	1.101598747
292	6	4	0.778525318	2.082427098	0.6	0.27212103	7.70687846	1.92083555	-5.78604291	0.994108161	0.029826574	-0.997722583	0.249236518
293	10	6	0.778513338	2.221328957	0.625	0.259518088	12.84499509	2.701085753	-10.14390933	0.99959329	0.002236019	-1.749176005	0.210283129
294	4	10	1	1.999830105	0.285714286	0.333352212	4	5.000424773	1.000424773	0.475439783	0.738855133	0.172509331	1.250106193
295	9	7	0.999943812	2.000056188	0.5625	0.333314604	9.000505718	3.499901674	-5.500604044	0.9840612	0.049945282	-0.948502623	0.388856114
296	9	0	0.778513338	2.082792833	1	0.2720832	11.56049558	0	-11.56049558	1	8.17E-06	-1.99344659	0
297	23.01147293	1	0.85891315	1.362790603	0.958353242	0.386601116	26.79138504	0.733788447	-26.05759659	1	4.93E-09	-4.493269923	0.02738897
298	5	1	1	1.999760965	0.833333333	0.333359895	5	0.500059766	-4.499940234	0.998627602	0.017839207	-0.775952074	0.100011953
299	13	9	1	2	0.590909091	0.333333333	13	4.5	-8.5	0.996512578	0.011603115	-1.465706717	0.346153846
300	18.03866158	41	0.857711789	2.035984893	0.305539812	0.296406944	21.03114567	20.13767398	-0.893471691	0.622816378	0.48786044	-0.15406676	0.957516737
301	7	3	0.999943629	1.999992538	0.7	0.333321635	7.000394617	1.500005596	-5.50038902	0.996596903	0.019657643	-0.948465545	0.214274434
302	7	7.000114207	0.999932278	2.000067722	0.499995921	0.333310759	7.000474087	3.499938592	-3.500535495	0.942402409	0.149424358	-0.603618634	0.499957367
303	0	20	0.193823864	2.806006479	0	0.064611609	0	7.127567292	7.127567292	0.262929622	0.737070378	1.229049795	0
304	8	2.000037896	0.778513338	2.221486662	0.799996968	0.259504446	10.27599607	0.90031506	-9.375681007	0.999959092	0.000548406	-1.616705721	0.08761341
305	8	3	1	2	0.727272727	0.333333333	8	1.5	-6.5	0.998628258	0.008823181	-1.120834548	0.1875
306	10.00094661	2	0.778895956	2.221104044	0.83334648	0.259631985	12.83990054	0.90045309	-11.93944745	0.999996693	5.37E-05	-2.058791567	0.070129289
307	16	4	1.006251191	1.993748809	0.8	0.335417064	15.90060229	2.006270791	-13.8943315	0.999996864	2.74E-05	-2.395884118	0.126175773

308	5	1	1	1.999977496	0.833333333	0.333335834	5	0.500005626	-4.499994374	0.998628196	0.017833265	-0.775961409	0.100001125
309	6	0	0.778513338	2.221486662	1	0.259504446	7.706997051	0	-7.706997051	1	0.0003054	-1.328964393	0
310	19.04541743	15.00082528	0.858072481	1.380726326	0.55939851	0.383273601	22.19558119	10.86444503	-11.33113616	0.987845156	0.028089358	-1.95389675	0.489486846
311	26	8	0.778577231	2.221422769	0.764705882	0.259525744	33.39424655	3.60129558	-29.79295097	1	1.07E-09	-5.137379805	0.107841798
312	2.5	18.5	0.202256788	2.797715029	0.119047619	0.067419563	12.36052458	6.612539093	-5.747985491	0.892856879	0.291864207	-0.991160111	0.534972367
313	8	12	0.778524609	2.221226875	0.4	0.259529702	10.27584729	5.402419778	-4.873427515	0.949003423	0.121442423	-0.840354758	0.525739593
314	10	6	1.004695477	1.993501878	0.625	0.335099848	9.953264678	3.009778955	-6.943485723	0.995767369	0.016586556	-1.19730749	0.30239113
315	10	8	0.917218096	2.082781904	0.555555556	0.305739365	10.90253239	3.841016663	-7.061515729	0.992882746	0.023973132	-1.217660122	0.352304999
316	6	3	0.999650706	2.000275608	0.666666667	0.333225087	6.002096499	1.499793322	-4.502303177	0.991735359	0.042355911	-0.77635953	0.249878242
317	6	1	0.91652565	2.08347435	0.857142857	0.30550855	6.546461629	0.479967512	-6.066494117	0.999751593	0.00420121	-1.046082491	0.073317089
318	31.96656063	0	1	1.932350611	1	0.34102334	31.96656063	0	-31.96656063	1	1.67E-15	-5.512188544	0
319	6	13	0.812739461	2.076318299	0.315789474	0.281316446	7.382439624	6.261082419	-1.121357205	0.729908864	0.453593951	-0.193362446	0.848104792
320	8	3	0.778195177	2.081573361	0.727272727	0.272118239	10.28019735	1.441217521	-8.83897983	0.999743171	0.002169885	-1.524159071	0.140193566
321	14	7	1	1.999166763	0.666666667	0.333425941	14	3.501458772	-10.49854123	0.999594101	0.001832936	-1.81032734	0.250104198
322	12.57140694	0	0.823403343	2.065501531	1	0.285022657	15.26761708	0	-15.26761708	1	5.10E-07	-2.632688106	0
323	6	6	0.778575032	2.000180613	0.5	0.280188376	7.706386349	2.999729105	-4.706657244	0.973222068	0.088962953	-0.811597545	0.389252364
324	16.75	4.25022883	0.999631019	2.000229077	0.797610356	0.33322588	16.75618271	2.124871036	-14.63131167	0.999996626	2.57E-05	-2.522966094	0.126811164
325	3	8	0.812156606	2.076800337	0.272727273	0.28112451	3.693868865	3.852079497	0.158210633	0.624804722	0.636657828	0.027281222	1.042830604
326	3	7	0.77866837	2.221325985	0.3	0.259556612	3.852731298	3.151270929	-0.701460369	0.75313919	0.502908317	-0.120957079	0.817931666
327	10	10	0.99963391	2.000213441	0.5	0.333228259	10.00366225	4.999466455	-5.00419579	0.962448874	0.091724845	-0.862903927	0.49976362
328	1	3	0.812091123	2.076853357	0.25	0.281103056	1.231388906	1.444492934	0.213104028	0.684854155	0.732904514	0.036746824	1.17305989
329	12	1	1.637917386	1.223145773	0.923076923	0.572485574	7.326376837	0.81756404	-6.508812796	0.999290522	0.007597091	-1.122354192	0.111591863
330	3	3.000265373	0.889339797	1.998444896	0.499977887	0.307966103	3.373288827	1.501300026	-1.871988802	0.923011957	0.270607721	-0.322798419	0.445055287
331	11	3	1.776283059	1.223660524	0.785714286	0.592105488	6.192706698	2.451660359	-3.741046339	0.964956146	0.112514098	-0.645091382	0.395894797
332	12.9926808	6.000496392	1	2	0.684070952	0.333333333	12.9926808	3.000248196	-9.992432604	0.999610641	0.001905921	-1.723055951	0.230918333
333	8.000038023	4	0.9999809	1.999985283	0.666667723	0.333330724	8.000190829	2.000014717	-6.000176112	0.99614467	0.018757415	-1.034646874	0.249995876
334	22.00541749	1	0.999988703	1.930218945	0.956531978	0.341268887	22.00566608	0.518075943	-21.48759013	1	8.35E-10	-3.705235904	0.023542843
335	12	0	1.638209391	1.223120132	1	0.572534333	7.325070939	0	-7.325070939	1	0.001240562	-1.26310655	0
336	14	2.000454959	0.936281177	2.063380725	0.87497512	0.312128902	14.9527731	0.96950356	-13.98326954	0.999999705	5.03E-06	-2.411220246	0.06483771
337	4	0	0.778513338	2.221486662	1	0.259504446	5.137998034	0	-5.137998034	1	0.00453502	-0.885976262	0
338	19.00330456	44.05218482	0.997083149	2.002916851	0.301374309	0.33236105	19.05889652	21.99401578	2.935119261	0.35370745	0.741120123	0.506120472	1.154002581
339	24	1	1.000119231	1.999880769	0.96	0.333373077	23.99713881	0.500029809	-23.497109	1	6.04E-11	-4.051749468	0.02083706
340	18	5	0.999872843	2.000063761	0.782608696	0.333297991	18.00228912	2.499920301	-15.50236882	0.999998336	1.31E-05	-2.673167777	0.138866801
341	5	9	0.917403369	2.082596631	0.357142857	0.305801123	5.450165292	4.321528166	-1.128637126	0.765654798	0.43479915	-0.194617767	0.7929169
342	3	4	0.812278095	1.409355558	0.428571429	0.36562198	3.693316389	2.838176623	-0.855139765	0.773624158	0.503425387	-0.147456953	0.7684629
343	7	6.000113809	0.999909837	1.999885736	0.538456825	0.333325992	7.000631201	3.000228313	-4.000402888	0.965347522	0.103533366	-0.68981381	0.4285654
344	9	3	0.890354284	1.992881884	0.75	0.3088038	10.10833571	1.505357655	-8.602978055	0.99973041	0.002124704	-1.483463849	0.148922404
345	10.00063488	8.53E-05	0.999999998	1.999964321	0.99999147	0.333337297	10.0006349	4.27E-05	-10.00059224	1	1.70E-05	-1.724462967	4.27E-06
346	14	0	1.778159674	1.221423494	1	0.592802258	7.873308681	0	-7.873308681	1	0.000661817	-1.35764252	0
347	16.78610112	3.000530879	0.778558368	2.221441632	0.848355653	0.259519456	21.56049155	1.350713355	-20.20977819	0.99999999	1.40E-07	-3.484895017	0.062647614
348	13	2.000536851	0.999967679	1.998094208	0.86663565	0.333538038	13.00042019	1.001222486	-11.99919771	0.999997819	3.17E-05	-2.069094667	0.077014625
349	11	0	1	2	1	0.333333333	11	0	-11	1	5.65E-06	-1.896796928	0

350	4	2.000075864	0.91711741	2.08288259	0.666658237	0.305705803	4.361491729	0.96024417	-3.401247559	0.988059927	0.075095742	-0.586497811	0.220164162
351	14	0	1	2	1	0.333333333	14	0	-14	1	2.09E-07	-2.41410518	0
352	44.36376383	1	1	1.999933335	0.977955974	0.333340741	44.36376383	0.500016667	-43.86374717	1	-6.66E-16	-7.563692805	0.011270835
353	13	0	0.778513338	2.221486662	1	0.259504446	16.69849361	0	-16.69849361	1	2.42E-08	-2.879422852	0
354	4	1	0.917215584	2.082784416	0.8	0.305738528	4.361024898	0.480126504	-3.880898394	0.997328527	0.033002964	-0.669206923	0.110094878
355	4	0	0.812183556	2.117617556	1	0.27721457	4.924995059	0	-4.924995059	1	0.005905602	-0.849246863	0
356	13.03215422	2.000234748	0.999983834	1.998130454	0.866938332	0.333537597	13.0323649	1.001053131	-12.03131177	0.999997753	3.22E-05	-2.074632291	0.076812853
357	34.00034291	1	0.999990291	1.99948177	0.971428851	0.333388767	34.00067302	0.500129591	-33.50054343	1	1.44E-15	-5.776702532	0.014709403
358	0	2	0.001499556	1.28187404	0	0.001168449	0	1.560215698	1.560215698	0.997664468	0.002335532	0.269037486	0
359	3.79E-05	0.000151452	1	2	0.200006058	0.333333333	3.79E-05	7.57E-05	3.79E-05	0.999938594	7.68E-05	6.53E-06	1.999924274
360	41.02909311	1	0.999985726	1.998421511	0.976206957	0.33350564	41.02967877	0.500394934	-40.52928384	1	-1.33E-15	-6.988711005	0.012195926
361	0	2	0.000343195	2.999628556	0	0.000114399	0	0.66674922	0.66674922	0.999771215	0.000228785	0.114971625	0
362	15	8.000267543	0.999197931	2.000745709	0.652166327	0.333072234	15.01204069	3.99864286	-11.01339783	0.999589021	0.001733042	-1.899107196	0.266362378
363	6.002159254	7	0.77857378	2.221347759	0.461627883	0.259531381	7.709172094	3.151240039	-4.557932055	0.970362174	0.09359108	-0.785951956	0.40876504
364	7	0	1	1.93003733	1	0.34129258	7	0	-7	1	0.000539371	-1.20705259	0
365	0	0	0	1.280795552	0	0	0	0	0	0	0	0	0
366	21	2	0.778513338	2.000101742	0.913043478	0.280180347	26.97448968	0.999949131	-25.97454055	1	3.38E-10	-4.478948064	0.037070178
367	4	3.79E-05	1	2	0.999990529	0.333333333	4	1.89E-05	-3.999981058	0.999999957	0.012346507	-0.689741071	4.74E-06
368	10	4	0.778513338	2.00019151	0.714285714	0.280171296	12.84499509	1.999808509	-10.84518658	0.999874934	0.000925982	-1.870101507	0.15568776
369	20.02166585	5.000038291	0.77866955	2.220321788	0.800171952	0.259643814	25.71266058	2.251943083	-23.46071749	0.999999998	2.51E-08	-4.04547426	0.087581099
370	8	1	0.778581247	2.221418753	0.888888889	0.259527082	10.27509978	0.450162761	-9.824937023	0.999994659	0.000142497	-1.694173669	0.043811035
371	2	3	0.812140202	1.409486384	0.4	0.365561075	2.462628984	2.128434892	-0.334194091	0.740187355	0.601075762	-0.057627121	0.864293772
372	5	4	0.778513338	2.221486662	0.555555556	0.259504446	6.422497543	1.800596001	-4.621901542	0.987876804	0.056707746	-0.796982604	0.280357601
373	10	11	0.860698623	2.069824322	0.476190476	0.293701376	11.61846869	5.314460692	-6.304008	0.977447977	0.059310452	-1.087038455	0.4574149
374	19	6	0.859603531	1.363328071	0.76	0.386698147	22.10321307	4.400994984	-17.70221809	0.999969877	0.000166372	-3.052501171	0.199111096
375	33.00685554	0	1	1.999119944	1	0.333431146	33.00685554	0	-33.00685554	1	3.33E-16	-5.691572925	0
376	1	0	1	1.778671117	1	0.35988426	1	0	-1	1	0.35988426	-0.172436084	0
377	18	2	1.000028315	1.930618066	0.9	0.341231314	17.99949034	1.03593768	-16.96355266	0.999999982	3.43E-07	-2.925128598	0.057553723
378	3.001022844	6.00003792	0.778513338	2.00013239	0.333407686	0.280177257	3.854812368	2.999820386	-0.854991981	0.773688595	0.483214642	-0.147431469	0.778201401
379	6	1	1	2	0.857142857	0.333333333	6	0.5	-5.5	0.999542753	0.006858711	-0.948398464	0.083333333
380	22.00147828	0	1	2	1	0.333333333	22.00147828	0	-22.00147828	1	3.20E-11	-3.793848764	0
381	19.00208278	2.000037946	0.778558366	2.221441634	0.904769715	0.259519455	24.40675435	0.900333331	-23.50642102	1	8.84E-10	-4.053355198	0.036888696
382	18.00459067	9	1.770842982	1.222979142	0.666723332	0.591499063	10.16724286	7.359078898	-2.808163962	0.838985228	0.277560518	-0.484228798	0.723802805
383	18	1	0.859242523	2.029667993	0.947368421	0.297427877	20.94868389	0.492691417	-20.45599248	1	4.53E-09	-3.527351244	0.023518968
384	3	0	0.778513338	2.221486662	1	0.259504446	3.853498526	0	-3.853498526	1	0.017475693	-0.664482197	0
385	8.000492312	1	0.999627586	2.000372414	0.888894966	0.333209195	8.003472917	0.499906914	-7.503566003	0.999949328	0.00096283	-1.29388554	0.062461249
386	18	0	0.848335393	1.373151269	1	0.381877329	21.21802313	0	-21.21802313	1	2.98E-08	-3.658752826	0
387	3	12	0.778579518	2.221375309	0.2	0.259530414	3.853170972	5.402058783	1.54888781	0.427413755	0.78869028	0.267084149	1.401977442
388	8	4	1	2	0.666666667	0.333333333	8	2	-6	0.996144445	0.018758432	-1.034616506	0.25
389	12	10.0002683	0.915557395	2.084442605	0.545447893	0.305185798	13.10676979	4.797574314	-8.309195475	0.994899869	0.016170743	-1.432805132	0.366037887
390	11	1	0.778513338	2.221486662	0.916666667	0.259504446	14.12949459	0.450149	-13.67934559	0.999999907	3.29E-06	-2.35881279	0.031858818
391	15	83.4298905	0.858425227	2.047001039	0.152392733	0.295455864	17.4738574	40.75713148	23.28327408	0.00076761	0.999690451	4.014876612	2.332463322

392	7	0	0.917207167	2.082792833	1	0.305735722	7.631863609	0	-7.631863609	1	0.000249703	-1.316008677	0
393	18	3	0.999974723	2.000025277	0.857142857	0.333324908	18.000455	1.499981042	-16.50047396	0.999999916	1.10E-06	-2.84527712	0.083330174
394	9.003029385	2	0.778516752	2.221471949	0.818231877	0.259506561	11.56433611	0.900303963	-10.66403215	0.999988331	0.000172778	-1.838863947	0.077851764
395	4	2.000151604	0.812154936	2.076759749	0.666649822	0.281128044	4.925168616	0.963111696	-3.96205692	0.991930923	0.056492093	-0.683201581	0.195548979
396	6	4	0.999091553	2.000908447	0.6	0.333030518	6.005455635	1.999091966	-4.00636367	0.980441537	0.076253801	-0.690841664	0.332879316
397	15.95138573	0	0.778513338	2.221486662	1	0.259504446	20.48954714	0	-20.48954714	1	1.21E-09	-3.533137278	0
398	9	1	0.999921031	1.99939948	0.9	0.33338252	9.000710776	0.500150175	-8.500560601	0.99998304	0.000356087	-1.465803384	0.055567853
399	15	10	0.999894749	2.000105251	0.6	0.33329825	15.00157894	4.999736885	-10.00184205	0.998352495	0.005593845	-1.724678479	0.33328071
400	3	0	0.916686487	2.083313513	1	0.305562162	3.272656512	0	-3.272656512	1	0.0285298	-0.564324074	0
401	6	4	0.812240864	2.117583185	0.6	0.27723196	7.386971357	1.888945865	-5.498025492	0.993404422	0.032613633	-0.948057987	0.25571317
402	13	2	0.858024697	2.030919788	0.866666667	0.297002833	15.15107904	0.984775476	-14.16630356	0.999999549	7.71E-06	-2.442781916	0.064997052
403	0	1	5.08E-05	2.999949201	0	1.69E-05	0	0.333338978	0.333338978	0.999983067	1.69E-05	0.057479668	0
404	18	0	0.778513338	2.221486662	1	0.259504446	23.12099115	0	-23.12099115	1	2.85E-11	-3.98689318	0
405	10.00254106	10.00026609	0.999141751	2.000562935	0.500056866	0.333080038	10.0111331	4.998726065	-5.012407038	0.962587586	0.091430646	-0.864319843	0.499316712
406	19.99511549	1	0.859335182	1.362270413	0.952369874	0.386808164	23.26812158	0.734068648	-22.53405294	0.999999998	7.93E-08	-3.885683852	0.031548256
407	6	1	0.778513338	2.221464148	0.857142857	0.259506394	7.706997051	0.450153562	-7.256843489	0.999920743	0.001662355	-1.251341676	0.058408425
408	3	1	0.999462842	2.000537158	0.75	0.333154281	3.00161234	0.499865747	-2.501746594	0.987680826	0.110952017	-0.431391387	0.166532413
409	9	3.000037954	1	2	0.749997628	0.333333333	9	1.500018977	-7.499981023	0.99945617	0.003855689	-1.29326736	0.166668775
410	20	8	0.999613038	2.00033057	0.714285714	0.33321061	20.00774223	3.999338969	-16.00840326	0.999992219	4.24E-05	-2.760426375	0.199889569
411	10.00030472	3.000609338	0.999909846	2.000086585	0.769200125	0.333303678	10.00120637	1.50023972	-8.500966647	0.999787366	0.001647498	-1.465873402	0.150005876
412	5	2	0.812116983	2.076792047	0.714285714	0.281115457	6.156748477	0.963023719	-5.193724758	0.997377755	0.021675151	-0.89558556	0.156417583
413	11	4	0.859535033	2.0702905	0.733333333	0.293374135	12.79761683	1.932096003	-10.86552082	0.999928107	0.000543446	-1.873607865	0.150973109
414	3	1.000265117	0.778524607	2.221475393	0.749950294	0.259508202	3.853442747	0.450270627	-3.403172121	0.995462494	0.056312928	-0.586829675	0.116848921
415	17	6	0.778539035	2.221375909	0.739130435	0.25952037	21.83577088	2.701028662	-19.13474222	0.999999765	2.06E-06	-3.299520023	0.123697426
416	14.00261364	6	0.778603592	2.22018813	0.700039199	0.259639103	17.9842654	2.702473687	-15.28179171	0.999993657	4.67E-05	-2.635132324	0.150268784
417	22.00107407	1	1.771796117	1.22183454	0.956523769	0.591855282	12.41738474	0.818441423	-11.59894332	0.999994221	9.73E-05	-2.000076368	0.065910934
418	18	12.0119403	0.813279508	2.116550165	0.599761289	0.277585935	22.13261224	5.675244791	-16.45736745	0.999948545	0.00021992	-2.837844002	0.256420016
419	2	4.000227356	0.812172479	2.076777554	0.333320703	0.281130677	2.462531114	1.926170353	-0.536360761	0.778408498	0.538191678	-0.092487949	0.782191276
420	5	3.001364463	0.888890277	2.000099365	0.624893419	0.307682057	5.624991215	1.500607678	-4.124383537	0.987071442	0.064205466	-0.711192547	0.266775115
421	3	4.999658522	0.190657423	2.809342577	0.375016007	0.063552474	15.73502856	1.779654273	-13.95537429	0.990072723	0.011277458	-2.406410097	0.113101433
422	12	6	0.859384768	2.070473487	0.666666667	0.293319572	13.96347765	2.897887869	-11.06558978	0.99979053	0.001147253	-1.908106972	0.207533391
423	2	3.79E-05	0.778513338	2.221486662	0.999981067	0.259504446	2.568999017	1.70E-05	-2.568981971	0.999999725	0.067345891	-0.442985192	6.64E-06
424	13	0	1.592519221	1.188323294	1	0.572675084	8.163166779	0	-8.163166779	1	0.000712538	-1.407624515	0
425	9	2	0.778513338	2.221486662	0.818181818	0.259504446	11.56049558	0.900298001	-10.66019758	0.999988359	0.000172598	-1.838202728	0.077877111
426	2.000189581	5	0.812273227	2.076688393	0.28573363	0.281164423	2.462459078	2.407679465	-0.054779612	0.68937047	0.629238126	-0.009445982	0.977754102
427	62	0	0.815198449	2.073706425	1	0.282182517	76.05510059	0	-76.05510059	1	1.11E-16	-13.11464374	0
428	4.003968254	9	0.999423454	2.000576546	0.30790357	0.333141151	4.006278059	4.498703144	0.492425085	0.553143094	0.67639682	0.084911853	1.122913357
429	3	1	0.778513338	2.221486662	0.75	0.259504446	3.853498526	0.450149	-3.403349525	0.99546498	0.056297712	-0.586860266	0.116815667
430	3	2	0.778533978	2.221448154	0.6	0.259512872	3.853396365	0.900313607	-2.953082759	0.98203015	0.113802111	-0.509218028	0.233641578
431	13	1.00003909	0.999954919	2.000016491	0.928568836	0.333321483	13.00058608	0.500015422	-12.50057066	0.999999791	6.06E-06	-2.155549456	0.038460991
432	13	4	0.778817573	2.221114811	0.764705882	0.259611709	16.69197056	1.80089745	-14.89107311	0.999998129	1.93E-05	-2.567758338	0.107890045
433	21.99530355	2	1.777680598	1.221854146	0.916650356	0.592652111	12.3730346	1.636856581	-10.73617802	0.999936736	0.00052864	-1.851304498	0.13229225

434	9	0	0.778513338	2.221486662	1	0.259504446	11.56049558	0	-11.56049558	1	5.34E-06	-1.99344659	0
435	12	8.001633739	0.914117314	2.085882686	0.599950992	0.304705771	13.12741791	3.836090012	-9.291327902	0.998496663	0.005914476	-1.602160202	0.292219691
436	0	2	0.000492535	1.281104903	0	0.000384313	0	1.561152404	1.561152404	0.999231521	0.000768479	0.269199008	0
437	4	1	1	2	0.8	0.333333333	4	0.5	-3.5	0.995884774	0.04526749	-0.603526295	0.125
438	5	1	0.778513338	2.000147763	0.833333333	0.280175707	6.422497543	0.499963062	-5.922534481	0.999516292	0.007940124	-1.021258655	0.077845582
439	6	1	0.778520378	2.221479622	0.857142857	0.259506793	7.706927356	0.450150427	-7.256776929	0.999920742	0.001662369	-1.251330198	0.058408547
440	25	2.000190186	1	2	0.925919404	0.333333333	25	1.000095093	-23.99990491	1	1.91E-10	-4.138449626	0.040003804
441	13	3	0.812247515	2.117534866	0.8125	0.277238173	16.00497357	1.416741726	-14.58823184	0.999998955	1.31E-05	-2.515537576	0.088518842
442	35	2	1.775172872	1.223265683	0.945945946	0.592032433	19.71638962	1.634967798	-18.08142183	0.9999999	1.29E-06	-3.117889579	0.0829243
443	23	1	1.644361041	1.223432567	0.958333333	0.573388906	13.9871959	0.817372389	-13.16982351	0.999998405	3.01E-05	-2.270952797	0.058437187
444	4	7	0.999932626	2.000067374	0.363636364	0.333310875	4.000269513	3.4998821	-0.500387413	0.711058952	0.527379049	-0.086284846	0.874911575
445	18	0	1.777559255	1.221782726	1	0.592649743	10.12624471	0	-10.12624471	1	8.14E-05	-1.746129987	0
446	15	1	1.637991928	1.223171147	0.9375	0.572491635	9.157554285	0.81754708	-8.340007205	0.999866861	0.001723889	-1.438118186	0.089275701
447	4	2	0.812129864	2.117662932	0.666666667	0.277197031	4.925320661	0.944437365	-3.980883295	0.992448698	0.053819801	-0.686447928	0.191751447
448	2	0	0.778513338	2.221486662	1	0.259504446	2.568999017	0	-2.568999017	1	0.067342557	-0.442988131	0
449	2	1	0.812107068	2.117694044	0.666666667	0.277188463	2.462729459	0.472211745	-1.990517714	0.978702656	0.187905644	-0.34323708	0.191743248
450	10	3	0.88909308	1.999806284	0.769230769	0.307761873	11.24741631	1.500145301	-9.747271005	0.999905586	0.000817651	-1.680781245	0.133376881
451	16	16.00909306	0.999753515	2.000246485	0.499857961	0.333251172	16.00394474	8.003560149	-8.000384588	0.98359118	0.037678012	-1.379554991	0.500099212
452	4	1	1.637733403	1.223166474	0.8	0.572453939	2.442399962	0.817550204	-1.624849758	0.938524405	0.29104558	-0.28018273	0.334732319
453	14	6.000189797	0.778587737	2.221397972	0.699993357	0.259530482	17.98127473	2.701087276	-15.28018745	0.999993694	4.65E-05	-2.634855691	0.150216674
454	15	7.001738526	0.778513338	2.000202952	0.681764306	0.280170142	19.26749263	3.500514045	-15.76697858	0.999982715	0.000105259	-2.718796048	0.181679792
455	19.01848205	3.00022932	0.778520167	2.221265065	0.86374183	0.259525301	24.42901656	1.350684962	-23.0783316	1	4.94E-09	-3.979537134	0.05529019
456	5	6.001140202	0.778513339	2.221486659	0.454498344	0.259504447	6.422497532	2.701407266	-3.721090266	0.959149045	0.130518263	-0.641650235	0.420616318
457	9	4	1.000210158	1.779071194	0.692307692	0.359880858	8.998108972	2.24836421	-6.749744762	0.996806278	0.015349368	-1.163899557	0.249870747
458	2	7	0.779011013	2.220289577	0.222222222	0.25973089	2.567357799	3.152741909	0.58538411	0.573418356	0.722435409	0.100941344	1.228010334
459	7	5.000265252	0.999796606	2.000203394	0.583320439	0.333265535	7.001424044	2.499878396	-4.501545648	0.981262441	0.066388369	-0.776228905	0.357052848
460	24	4.000116171	0.858079658	2.030895234	0.857139301	0.297018732	27.96943125	1.969631965	-25.99979929	1	1.19E-09	-4.483303583	0.07042088
461	12	16.00155929	0.778643735	2.221073978	0.428547563	0.259572336	15.41141276	7.204424279	-8.206988482	0.984513144	0.038723523	-1.415180958	0.467473319
462	26.00075903	0	1.60465836	1.198290851	1	0.572489274	16.20329889	0	-16.20329889	1	5.04E-07	-2.794033414	0
463	4	2.000416971	0.778513338	2.000117315	0.66662034	0.280178777	5.137998034	1.000149819	-4.137848215	0.992055542	0.055850907	-0.713514344	0.194657494
464	8	3	0.812170453	2.117667838	0.727272727	0.27720658	9.850149027	1.416652766	-8.433496261	0.999700224	0.002472251	-1.454239072	0.14382044
465	17	7	0.858633666	2.030448009	0.708333333	0.297199513	19.79889756	3.447515016	-16.35138254	0.999993902	3.84E-05	-2.819568378	0.174126615
466	10.00102521	15	0.997975981	2.002024019	0.400024604	0.33265866	10.02130853	7.492417604	-2.528890924	0.823974649	0.301770356	-0.436072049	0.747648631
467	5	16.00003809	0.888605277	2.000610416	0.238094806	0.307559342	5.626795305	7.99757812	2.370782815	0.334843657	0.821581627	0.408808505	1.421338024
468	13	43.36643357	0.778801345	2.221164724	0.230633715	0.259603384	16.69231838	19.52418616	2.831867781	0.373788209	0.7366377	0.488316192	1.169650957
469	18.00337045	3	0.859528386	1.362531753	0.857165782	0.386815987	20.94563801	2.20178355	-18.74385446	0.999998774	1.28E-05	-3.232116869	0.105118954
470	28.06797222	2.000203558	1.596750809	1.189276792	0.933477722	0.573128137	17.57817943	1.681865459	-15.89631397	0.999998585	1.56E-05	-2.741098136	0.095679161
471	4.000340857	3.00022729	0.812131732	2.076649583	0.571430886	0.281132991	4.925729041	1.444744127	-3.480984914	0.97831518	0.102903065	-0.600247408	0.293305644
472	9	25	0.779494787	2.219413949	0.264705882	0.259926145	11.54593995	11.26423487	-0.281705076	0.613299167	0.539909076	-0.04857612	0.975601374
473	2	2	0.778513338	2.221486662	0.5	0.259504446	2.568999017	0.900298001	-1.668701017	0.943702288	0.27785486	-0.287744269	0.350447001
474	12	1	1	2	0.923076923	0.333333333	12	0.5	-11.5	0.999999373	1.69E-05	-1.98301497	0.041666667
475	14	8.000114303	0.85931622	2.029578234	0.63636033	0.297455042	16.29202344	3.941761973	-12.35026147	0.999778681	0.001026995	-2.129630728	0.241944286

476	2	0	0.812087971	2.117713141	1	0.277181945	2.462787373	0	-2.462787373	1	0.076829831	-0.424673411	0
477	4	13	0.915754301	2.084245699	0.235294118	0.305251434	4.367983851	6.23726848	1.869284629	0.370660026	0.810877903	0.322332122	1.42795136
478	7.001477049	2.000075855	0.999704216	1.930305822	0.777807688	0.341194809	7.003548584	1.036144549	-5.967404035	0.998847341	0.009564468	-1.028995785	0.14794565
479	21	5.000083205	0.778518367	2.221464988	0.807689723	0.259507562	26.97431542	2.250804416	-24.72351101	0.999999999	7.91E-09	-4.263225429	0.083442504
480	23.00094905	1.000151979	0.999966146	1.999958842	0.958328913	0.333330383	23.00172776	0.500086281	-22.50164148	1	1.74E-10	-3.880094948	0.021741249
481	9.001022263	0	0.778513338	2.082792833	1	0.2720832	11.56180867	0	-11.56180867	1	8.18E-06	-1.993673015	0
482	3.998144783	0	0.778513338	2.221486662	1	0.259504446	5.135615008	0	-5.135615008	1	0.004615369	-0.885565343	0
483	7.003597122	1	0.812186983	2.117607887	0.87505618	0.27721633	8.623133921	0.472230957	-8.150902963	0.999964942	0.000763702	-1.405509791	0.054763264
484	3	2	0.778695472	2.221276278	0.6	0.259567602	3.852597205	0.900383271	-2.952213934	0.98201598	0.113862751	-0.509068211	0.233708125
485	21	7	0.778625937	2.000139485	0.75	0.28020571	26.97058882	3.499755918	-23.4708329	0.999999959	3.36E-07	-4.047218521	0.12976194
486	3	1	0.778513338	2.221486662	0.75	0.259504446	3.853498526	0.450149	-3.403349525	0.99546498	0.056297712	-0.586860266	0.116815667
487	3	2	0.812169608	2.076633385	0.6	0.281143993	3.693809729	0.963097297	-2.730712431	0.975787788	0.139046144	-0.470873359	0.260732785
488	24.01791124	2	0.778229305	2.08245274	0.923129878	0.272043272	30.86225498	0.960405949	-29.90184903	1	4.88E-12	-5.15615776	0.031119111
489	15	14.00756748	0.778649018	2.221350979	0.517106442	0.259549673	19.26413525	6.305877643	-12.95825761	0.99921778	0.002675552	-2.234471202	0.327337696
490	7.000720516	11	0.778513338	2.221486662	0.38891335	0.259504446	8.992422062	4.951639003	-4.040783059	0.930821525	0.16173519	-0.696776808	0.550645751
491	25	29.00553077	0.993783134	2.005767278	0.46291555	0.331310696	25.15639392	14.46106489	-10.69532903	0.984355656	0.030233642	-1.844260658	0.574846496
492	8.966719673	0	0.778513338	2.082792833	1	0.2720832	11.51774701	0	-11.51774701	1	1.37E-05	-1.986075195	0
493	0	7	0.000759769	2.99921906	0	0.000253258	0	2.333940889	2.333940889	0.99822854	0.00177146	0.402455628	0
494	3	5	0.812102772	2.117695978	0.375	0.27718722	3.69411373	2.361056569	-1.333057162	0.845202509	0.390105521	-0.229867157	0.639140195
495	5	10	0.778610537	2.221389463	0.333333333	0.259536846	6.421695784	4.501686969	-1.920008815	0.829842303	0.345378219	-0.331078802	0.701012181
496	19	4.00049607	0.999953658	2.000046342	0.82606914	0.333317886	19.00088054	2.000201688	-17.00067885	0.999999838	1.67E-06	-2.931530491	0.105268894
497	3	4	0.812150663	2.076429947	0.428571429	0.281159079	3.693895895	1.926383313	-1.767512582	0.897069137	0.310639975	-0.304782949	0.521504495
498	4	1	0.778520388	2.221479612	0.8	0.259506796	5.137951507	0.450150429	-4.687801078	0.998823089	0.017968277	-0.808346062	0.087612822
499	13	8	0.999582061	1.999881326	0.619047619	0.33325363	13.00543548	4.000237362	-9.005198115	0.998177364	0.006792013	-1.552821102	0.307581962
500	8	2	0.99989841	2.00010159	0.8	0.33329947	8.000812805	0.999949207	-7.000863597	0.999644673	0.003401476	-1.207201506	0.124980953
501	4.999129184	0	0.778513338	2	1	0.280190607	6.42137898	0	-6.42137898	1	0.001743173	-1.107277447	0
502	12	10.00095551	0.77857964	2.221352464	0.545430856	0.259532421	15.41268147	4.502192099	-10.91048938	0.998970919	0.004022848	-1.881362066	0.292109592
503	12	0	0.778513338	2	1	0.280190607	15.4139941	0	-15.4139941	1	2.34E-07	-2.657928787	0
504	24	1	1.000039524	1.999960476	0.96	0.333346508	23.99905146	0.500009881	-23.49904158	1	6.02E-11	-4.052082715	0.020834568
505	6	3	0.812590029	2.076121013	0.666666667	0.281298481	7.383797222	1.445002474	-5.938794748	0.997143763	0.018306266	-1.024062512	0.1956991
506	11	0	0.778513338	2	1	0.280190607	14.12949459	0	-14.12949459	1	8.36E-07	-2.436434721	0
507	8	3	1.000210411	1.778607001	0.727272727	0.359941034	7.998317063	1.686713253	-6.31160381	0.997444872	0.014744877	-1.088348247	0.21088352
508	13.00285377	19.00232231	0.86009136	2.069972624	0.406273465	0.293540129	15.11799138	9.179987256	-5.938004126	0.941019589	0.115599487	-1.02392618	0.607222681
509	9	1	0.812345948	2.117469458	0.9	0.277268645	11.07902368	0.472261829	-10.60676186	0.999997315	7.27E-05	-1.828988482	0.042626665
510	10	3.000037928	1	1.999943591	0.769228525	0.333339601	10	1.500061272	-8.499938728	0.999787319	0.001648053	-1.465696151	0.150006127
511	8	2	0.81227635	2.076645479	0.8	0.281169377	9.848864855	0.963091688	-8.885773167	0.999917964	0.000990295	-1.532227931	0.097787075
512	19	0	1.591040208	1.188065882	1	0.57250071	11.94187293	0	-11.94187293	1	2.50E-05	-2.059209808	0
513	6.00318411	9.000037965	0.778735115	2.221185772	0.400126325	0.259585217	7.708890989	4.051906904	-3.656984085	0.932211439	0.170085724	-0.630596016	0.525614762
514	9	3	0.8889849	2.000180546	0.75	0.307696086	10.12390649	1.499864603	-8.624041889	0.9997392	0.002065524	-1.487096014	0.148150776
515	6.998825935	4	0.81225587	2.117541085	0.63632482	0.277239646	8.616528597	1.888983419	-6.727545178	0.997515009	0.013848592	-1.160071547	0.219227894
516	5	39	0.812097627	2.106622044	0.113636364	0.278237624	6.156895221	18.51305036	12.35615514	0.007738109	0.997697993	2.13064701	3.006880855
517	1	1	0.188236161	2.811763839	0.5	0.062745387	5.31247554	0.355648645	-4.956826895	0.996063016	0.12155379	-0.85473582	0.066945936

518	4.5	16.5	0.778688605	2.221122673	0.214285714	0.259579198	5.778946771	7.428675687	1.649728916	0.428112903	0.751810376	0.284472794	1.285472246
519	26	4	0.999904218	1.999494051	0.866666667	0.333368272	26.00249056	2.000506077	-24.00198448	1	2.30E-09	-4.13880822	0.076935172
520	13	7	0.999297718	2.000702282	0.65	0.333099239	13.00913609	3.498771437	-9.510364649	0.999128164	0.003698666	-1.639930041	0.268947255
521	23	6	0.858400989	2.030562451	0.793103448	0.297131136	26.79400453	2.954846327	-23.8391582	0.999999995	4.82E-08	-4.110731094	0.110280131
522	18	3.81E-05	1.77777857	1.222083257	0.999997883	0.592620151	10.12499549	3.12E-05	-10.12496431	1	8.13E-05	-1.745909199	3.08E-06
523	7.997121648	4	0.812151767	2.117692291	0.666586693	0.277199657	9.846831556	1.888848543	-7.957983013	0.999087206	0.005656316	-1.37224343	0.191822977
524	4	3.79E-05	1	1.778601725	0.999990529	0.359893248	4	2.13E-05	-3.9999787	0.999999934	0.016777353	-0.689740664	5.33E-06
525	3	2.000113636	0.999958756	2.000041244	0.599986364	0.333319585	3.000123738	1.000036195	-2.000087542	0.95473428	0.209868263	-0.344887264	0.33333165
526	25	15.00386323	0.931908179	2.066226959	0.624939643	0.310829278	26.82667732	7.261478788	-19.56519853	0.999989511	4.16E-05	-3.373746224	0.270681259
527	6	26	0.917666324	2.082333676	0.1875	0.305888775	6.538324271	12.48599122	5.947666951	0.100102971	0.955841507	1.0255924	1.909662278
528	11	2	0.778513338	2.000045045	0.846153846	0.280186064	14.12949459	0.999977478	-13.12951712	0.999997744	3.60E-05	-2.26400252	0.070772346
529	10.00594517	2	0.859114171	1.362487953	0.833415864	0.386709286	11.64681658	1.467902887	-10.17891369	0.999775012	0.002084103	-1.75521202	0.126034687
530	6	1.000038008	0.917219812	2.082780188	0.857138203	0.305739937	6.541507195	0.480145727	-6.061361468	0.999750252	0.00421941	-1.045197437	0.073399862
531	3	0	1.638134431	1.22318309	1	0.572510537	1.831351532	0	-1.831351532	1	0.187650814	-0.315791087	0
532	3	2	1	2	0.6	0.333333333	3	1	-2	0.95473251	0.209876543	-0.344872169	0.333333333
533	9	4	0.916764017	2.083235983	0.692307692	0.305588006	9.817139231	1.920089723	-7.897049508	0.999232072	0.004631612	-1.361736295	0.195585463
534	7	0	0.778513338	2	1	0.280190607	8.99149656	0	-8.99149656	1	0.000135574	-1.550458459	0
535	3	1	1	1.778600607	0.75	0.359893393	3	0.562239772	-2.437760228	0.983223727	0.136129434	-0.420357828	0.187413257
536	5	5	0.999988746	2.000011254	0.5	0.333329582	5.000056268	2.499985933	-2.500070335	0.92344031	0.213120396	-0.431102339	0.49999156
537	5	27.13964127	0.999966325	2.000033675	0.15557112	0.333322108	5.000168378	13.56959216	8.56942378	0.020815306	0.993296405	1.477677882	2.713827042
538	7	0	1	2	1	0.333333333	7	0	-7	1	0.000457247	-1.20705259	0
539	3.001173709	0	0.778513338	2.221486662	1	0.259504446	3.855006154	0	-3.855006154	1	0.017488286	-0.664742166	0
540	3	0	1	1.778618733	1	0.359891045	3	0	-3	1	0.046613651	-0.517308253	0
541	11	0	1.637812156	1.222922387	1	0.572514552	6.71627693	0	-6.71627693	1	0.002165967	-1.158128495	0
542	14	6	0.999338318	2.000661682	0.7	0.333112773	14.00926968	2.999007805	-11.01026188	0.999834041	0.000872238	-1.898566446	0.214073101
543	15	8	1.776064848	1.223723188	0.652173913	0.592063448	8.445637565	6.537426175	-1.90821139	0.785990741	0.358799407	-0.3290445	0.774059522
544	6.001027085	14.00864455	0.995864071	2.004135929	0.299906325	0.33195469	6.025949984	6.989867478	0.963917494	0.484204649	0.698561717	0.166214158	1.159961084
545	1	3	0.999952163	2.000047837	0.25	0.333317388	1.000047839	1.499964123	0.499916285	0.59262094	0.802450237	0.086203607	1.49989237
546	3	3	0.999943692	2.000056308	0.5	0.333314564	3.000168934	1.49995777	-1.500211164	0.899881362	0.319578837	-0.258690539	0.49995777
547	4.000340935	1	1.778304782	1.22164589	0.800013636	0.592778007	2.249524928	0.818567809	-1.430957118	0.926793831	0.324580881	-0.246748642	0.363884747
548	3	2	0.999375719	1.99952843	0.6	0.333246969	3.001874013	1.000235841	-2.001638172	0.954775145	0.209748613	-0.345154649	0.333203804
549	1	2	0.997937332	2.00129215	0.333333333	0.332731236	1.002066931	0.999354342	-0.002712589	0.741543174	0.702900182	-0.000467748	0.997293006
550	1	7.000189394	0.917266113	2.082733887	0.124997041	0.305755371	1.090196166	3.361058001	2.270861835	0.244081641	0.946040215	0.391578523	3.082984609
551	20.18182163	5	0.778659711	2.221333236	0.80144407	0.259553847	25.91866683	2.250900459	-23.66776637	0.999999998	2.59E-08	-4.081176958	0.086844762
552	21.25	5.75	0.778732766	2.221176891	0.787037037	0.259585406	27.28792331	2.588717731	-24.69920558	0.999999998	2.09E-08	-4.259034296	0.094866791
553	0	2	0.000402851	1.281094558	0	0.000314359	0	1.561165011	1.561165011	0.99937138	0.00062862	0.269201182	0
554	0	1	5.07E-05	2.999949276	0	1.69E-05	0	0.333338969	0.333338969	0.999983092	1.69E-05	0.057479667	0
555	17	0	0.778513338	2.082792833	1	0.2720832	21.83649165	0	-21.83649165	1	2.45E-10	-3.765399115	0
556	37	4	0.99981955	2.000157885	0.902439024	0.33327569	37.00667785	1.999842127	-35.00683572	1	4.77E-14	-6.036441677	0.054040034
557	0.333333333	14.66666667	0.778508827	2.221445127	0.022222222	0.259506925	0.428168984	6.60230878	6.174139796	0.030374148	0.988963916	1.064644491	15.419867
558	9	2	0.999824898	2.000005829	0.818181818	0.333293772	9.001576198	0.999997086	-8.001579112	0.999870312	0.001370416	-1.37976097	0.111091332
559	4	2	0.999966184	1.999960547	0.666666667	0.333330202	4.00013527	1.000019727	-3.000115543	0.982168126	0.100134082	-0.517328177	0.249996477

560	23	0	1.775525981	1.221265505	1	0.592475649	12.95390788	0	-12.95390788	1	5.91E-06	-2.233721152	0
561	15.01076865	14	0.859976625	1.365653853	0.517420577	0.386396859	17.45485658	10.25149966	-7.203356921	0.947730575	0.105296007	-1.242118661	0.587315032
562	9	1	0.778513338	2.082806945	0.9	0.272081858	11.56049558	0.480121311	-11.08037427	0.999997777	6.17E-05	-1.910656351	0.041531205
563	8.000644917	6	0.889044291	1.999619074	0.571448313	0.307770127	8.999152235	3.000571498	-5.998580737	0.990047021	0.036547578	-1.034371774	0.333428241
564	0	12	0.189084235	2.810797024	0	0.063030573	0	4.26925171	4.26925171	0.457830837	0.542169163	0.736173048	0
565	12	2	0.778278522	2.082377087	0.857142857	0.272062991	15.4186447	0.960440841	-14.45820386	0.999999532	8.40E-06	-2.493116059	0.062290873
566	4	1	0.917211372	2.082788628	0.8	0.305737124	4.361044927	0.480125533	-3.880919394	0.997328588	0.033002407	-0.669210544	0.11009415

SUPPLEMENTARY TABLE 3. Infection and vaccination histories of subjects and HAI titers specific for A/California/04/2009/E3

2009/2010 season	Vaccination pH1N1	Infection pH1N1	HAI titers
FAM195	October 2010	November 2009	2560
FAM196	-	November 2009	640
FAM297	October 2009	-	320
FAM298	November 2009/ May 2010	-	40
FAM300	February 2010	-	640
FAM203	-	-	<10
FAM256	-	-	<10

SUPPLEMENTARY TABLE 4. Frequency of the HA protein mutations found in human subjects infected with pandemic A/California/04/2009-like viruses since 2009 (3786 sequences) and with seasonal H1N1 viruses (1041 sequences)

**PANDEMIC H1N1**		**SEASONAL H1N1**	
**POSITION**	**AMINOACID (FREQUENCY)**	**POSITION**	**AMINOACID (FREQUENCY)**
HEAD DOMAIN	T (99.52%)	**89**	**T (2.18%)**
89	A (0.26%)		**A (0.095%)**
	K (0.10%)		**-**
	L (0.079%)		**-**
	R (0.026%)		**-**
	-		**S (91.24%)**
	-		**P (6.37%)**
	-		**F ((0.095%)**
152	A (99.97%)	**152**	**A (9.89%)**
	S (0.03%)		**S (90.00%)**
	-		**T (0.095%)**
160	S (91.12%)	**160**	**S (99.81%)**
	G (8.66%)		**G (0.095%)**
	N (0.21%)		**-**
	-		**T (0.095%)**
237	V (99.84%)	**237**	**V (98.95%)**
	M (0.079%)		**M (0.095%)**
	L (0.026%)		**L (0.095%)**
	G (0.026%)		**-**
	E(0.026%)		**-**
	-		**I (0.85%)**
STALK DOMAIN			
41	V (99.86%)	**41**	**V (99.81%)**
	L (0.10%)		**L (0.095%)**
	A (0.026%)		**-**
	-		**G (0.095%)**
388	A (99.89%)	**388**	**A (99.81%)**
	V (0.052%)		**V (0.095%)**
	P (0.026%)		**P (0.095%)**
	T (0.026%)		**-**
466	V (99.39%)	**466**	**V (100%)**
	L (0.50%)		**-**
	A (0.10%)		**-**
526	R (98.91%)		**R (1.43%)**
	M (0.55%)		**M (0.095%)**
	K (0.39%)		**-**

G (0.10%)<br>E (0.026%)	**G (98.00 %)**<br>**E (0.047%)**
